# Molecular biomarkers for the prognosis of breast cancer: role of amino acid metabolism genes

**DOI:** 10.1007/s13105-025-01088-5

**Published:** 2025-06-10

**Authors:** Yudong Zhou, Shibo Yu, Lizhe Zhu, Yalong Wang, Chenglong Duan, Danni Li, Jinsui Du, Jiaqi Zhang, Jianing Zhang, Ruichao Ma, Jianjun He, Yu Ren, Bin Wang

**Affiliations:** 1 Department of Breast Surgery, the First Affiliated Hospital of Xi’an Jiaotong University, Xi’an, 710061 Shaan’xi Province China; 2 School of Medicine, Shaan’xi Province, Xi’an Jiaotong University, Xi’an, 710061 China; 3 Department of Pathology and Medical Biology, University of Groningen, University Medical Center Groningen, Groningen, The Netherlands; 4 Beijing university of post and telecommunication, Beijing, 100876 China

**Keywords:** Amino acid metabolism-related genes, Breast cancer, Prognosis, Immune microenvironment, Machine learning

## Abstract

The development of precise molecular biomarkers for breast cancer prognosis holds immense potential to improve treatment outcomes. This study aimed to investigate the role of amino acid metabolism genes as predictive markers for breast cancer prognosis and their association with the immune-tumour microenvironment. By employing advanced machine learning algorithms and bioinformatics analysis techniques, the impact of amino acid metabolism-related genes (AAMRGs) on the immune status and overall survival of patients with breast cancer was examined. An AAMRG-based risk model was established to assess the prognostic significance. Validated risk models (AIMP2, IYD, and QARS1) accurately predicted patient outcomes [1 y: 0.87 (0.96–0.78); 3 y: 0.82 (0.87–0.76); 5 y: 0.80 (0.86–0.75)]. Furthermore, this study revealed evidence suggesting that QARS1 may influence breast cancer cell proliferation through methionine metabolism. This analysis provides valuable insights into the mechanisms of breast cancer, emphasizing the significance of AAMRGs as prognostic biomarkers and potential therapeutic targets for optimizing personalized treatment strategies.

**Supplementary Information:**

The online version contains supplementary material available at 10.1007/s13105-025-01088-5.

## Key points

The development of precise molecular biomarkers for the prognosis of patients with breast cancer holds immense potential in enhancing the outcomes of breast cancer treatment.

This study employed machine learning algorithms and bioinformatics analysis techniques to conduct a comprehensive examination of AAMRGs and their impact on the immune status and overall survival of patients with breast cancer.

A risk model predicated on AAMRGs was developed to assess their prognostic significance and could markedly bolster the personalized treatment approach for patients with breast cancer.

**Supplementary Information:**

The online version contains supplementary material available at 10.1007/s13105-025-01088-5.

## Introduction

According to the latest estimates by the International Agency for Research on Cancer (IARC), female breast cancer (BRCA) is the second most common cancer in the world (11.6% of total cases) and the main cause of cancer death (6.9% of total cancer deaths), seriously endangering women’s health [1, 2]. Despite advancements in early detection and treatment, breast cancer remains a substantial global health challenge [3–7]. The development of precise molecular biomarkers for prognosis could significantly improve breast cancer treatment outcomes and effectively address this critical issue [8–10]. Indeed, several prognostic models based on multigene detection, such as OncotypeDX and EndoPredict, and breast cancer indicators have already been utilized in clinical practice [11–14]. However, the high costs and limited reproducibility of these models limit their broad clinical implementation. Consequently, identifying reliable prognostic biomarkers and conducting thorough analyses to assess their role in tumorigenesis remain imperative.

In recent years, significant progress has been made in understanding the reprogramming of amino acid metabolism within the tumour immune microenvironment (TIME) [15, 16]. Research has increasingly shown that disruptions in amino acid metabolism are critical for tumour initiation, progression, and treatment [17–19]. Cancer cell metabolism is highly dependent on the sulfur-containing amino acids (SAAs) methionine and cysteine because they participate in a variety of redox reactions to generate the energy required for tumour growth [20]. Additionally, SAA limitation in cancer may damage the glutamine (GSH)/GPX4 system, thereby increasing susceptibility to iron-mediated lipid peroxidation, leading to ferroptosis [21]. Furthermore, in some types of cancer, certain nonessential amino acids, such as leucine, serine, and arginine, may become essential amino acids, whereas in other types of cancer, increased amino acid synthesis may also be observed [22–25]. For example, in breast cancer, some scholars believe that elevated branched-chain amino acids may increase tumour proliferation by regulating the translation of a subset of mRNAs encoding mitochondrial subunits through the mTOR pathway [17, 26]. The study of AAMRGs not only deepens our understanding of tumour biology but also facilitates the exploration of these metabolic alterations in cancer and opens new avenues for therapeutic intervention.

The tumour immune microenvironment has emerged as a prominent research area in recent years. The complex composition of the TIME, which includes immune cells, fibroblasts, endothelial cells, and other cells, plays a crucial role in regulating tumour behaviour [27, 28]. Immune cells such as myeloid cells and lymphocytes are particularly significant, as they can both promote and suppress inflammation, affecting immune surveillance and evasion [19]. Given the notable acceleration in malignant tumour growth kinetics, scholars have speculated that there are significant alterations in amino acid metabolism within the TIME. The rewiring of amino acid metabolism in infiltrating immune cells directly influences their biological functions [29]. Hence, the key regulator of the amino acid metabolism pathway could be considered potential therapeutic targets and prognostic markers.

**Fig. 1:**
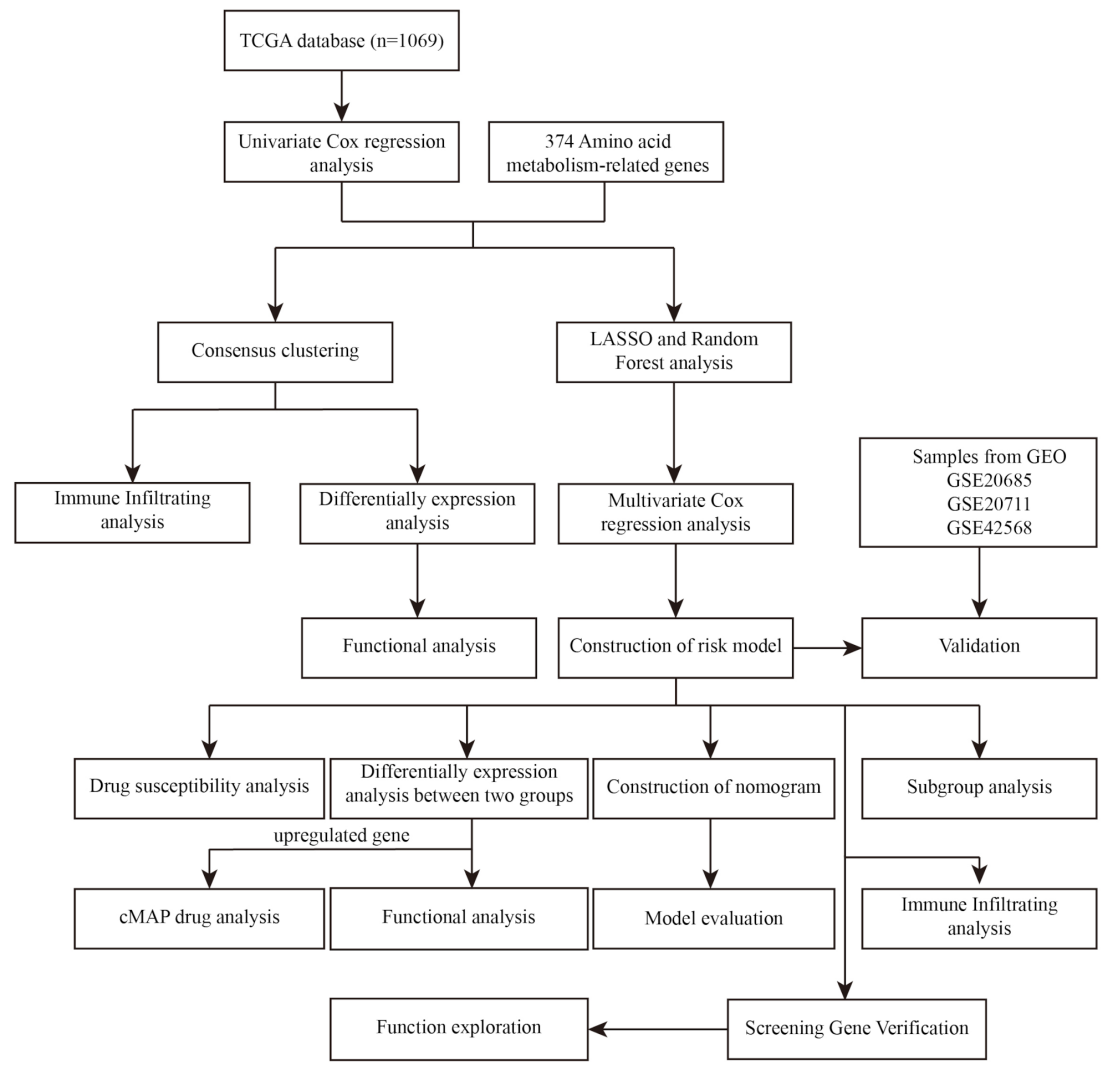
Flow chart of the data analysis process

In this study, we utilized a combination of machine learning algorithms and bioinformatics analysis techniques to perform a comprehensive analysis of AAMRGs. Our goal was to explore the influence of these genes on the immune status and overall survival rates of patients with breast cancer. Additionally, we developed a risk model based on AAMRGs to evaluate their prognostic value in breast cancer. This research has the potential to illuminate the molecular mechanisms of breast cancer, provide novel insights for targeted therapy and enhance personalized treatment strategies for patients with breast cancer. The flowchart of the data analysis process is depicted in Fig. 1.

## Materials and methods

### Data collection

Clinical information and sequenced RNA data were downloaded from The Cancer Genome Atlas (TCGA, https://portal.gdc.cancer.gov/) and Gene Expression Omnibus (GEO, https://www.ncbi.nlm.nih.gov/geo/) databases. The inclusion criteria were as follows: (a) samples from patients diagnosed with breast cancer; (b) samples with mapped clinical information and a gene expression matrix; and (c) samples with minimally complete clinical information, including survival time, survival status, age and sex. The exclusion criteria were as follows: (a) normal tissue samples; (b) samples without complete clinical information; (c) samples with no expression value for more than half of the genes; and (d) samples with biased expression values. A total of 1069 samples obtained from the TCGA database were included in the training cohort. Data obtained from the GEO database (GSE42568, GSE20711, and GSE20685) were included and defined as the validation cohort. The dataset of 374 amino acid metabolism genes was obtained from the REACTOME METABOLISM OF AMINO ACIDS AND DERIVATIVES database.

#### Unsupervised cluster analysis

Initially, 50 AAMRGs were found to be associated with the prognosis of patients with breast cancer through univariate Cox regression analysis. Subsequently, consensus clustering was conducted on the expression matrix of these 50 genes using the R package “ConsensusClusterPlus” [30]. Then, we identified the optimal number of clusters through the cumulative distribution function (CDF) and consensus matrices. The Kaplan-Meier test was then used to compare the OS of the two subclusters.

#### Immune infiltration analyses of invasive breast carcinoma subgroups

Using the Estimation of Stromal and Immune cells in Malignant Tumour tissues using Expression data (ESTIMATE) algorithm, we computed the stromal score, immune score, and ESTIMATE score [31]. The CIBERSORT and Microenvironment Cell Populations counter (MCP-counter) algorithms were used to calculate the abundances of ten types of infiltrating immune cells (T cells, CD8 + T cells, cytotoxic lymphocytes, B cells, NK cells, monocytic lineage cells, myeloid dendritic cells, neutrophils, endothelial cells and fibroblasts) [32, 33]. Based on 26 immune gene sets, the single-sample gene set enrichment analysis (ssGSEA) algorithm was used to assess the enrichment of the 26 infiltrating immune cells in the breast tumour samples [34].

#### Functional analyses

Differentially expressed genes (DEGs) between the two clusters were identified using the R package “Limma.” The fold change (FC) was set to 2.0, and the false discovery rate (FDR) was set to less than 0.05. Gene Ontology (GO) analysis and Kyoto Encyclopedia of Genes and Genomes (KEGG) analysis were subsequently conducted using the R package “clusterProfiler” to enrich the associated pathways [35]. Moreover, according to the “KEGG biological process” gene set, gene set enrichment analysis (GSEA) was conducted to analyse the differences between clusters.

#### Machine learning

To downsize the previously filtered prognostic genes, we performed least absolute shrinkage and selection operator (LASSO) analysis using the R package “glmnet” [36]. The minimum lambda value was determined as the optimal value. Next, the random forest (RF) algorithm, which integrates multiple trees through the idea of ensemble learning to gain better accuracy, was employed to narrow down the candidate biomarkers using the R package “randomForest” [37]. Genes that were identified by the LASSO model and had MeanDecreaseGini > 5 according to the RF model were defined as hub genes for developing a prognostic model of amino acid metabolism-related genes, followed by AIMP2, IYD, KYAT3, PSMD10, QARS1, SLC5A5, and TH.

The hub genes used to establish the risk model were determined by multivariate Cox regression analysis. The risk score of each patient in the training and verification cohorts was calculated as follows: risk score = 1.585837 × expression value of AIMP2 + 1.538501 × expression value of IYD + 0.627543 × expression value of QARS1. Patients were divided into high-risk and low-risk groups according to the average value. Survival differences between groups were assessed using both the log-rank and Kaplan-Meier tests. To evaluate the prediction accuracy of the prognostic signature, time-dependent receiver operating characteristic (ROC) curves were generated. Additionally, chi-square tests were used to test for differences in clinicopathological features between subgroups.

#### The construction of nomogram and the assessment of prognostic marker prediction model

Using the R package “rms”, we constructed a nomogram based on the risk score and clinical features. The performance of the nomogram in predicting the prognosis of patients with breast cancer was evaluated by determining the area under the ROC curve. Additionally, calibration curves were generated to assess the predictive efficiency of the nomogram, specifically for amino acid metabolism-related genes.

#### Connectivity map (cMAP) and drug susceptibility analysis

Connectivity map (CMap) (https://clue.io) is a gene expression profile database that focuses on gene expression signature perturbations [38]. It aims to uncover connections and relationships between diseases, genes, and small molecule compounds. In this study, the genes upregulated in the high-risk group were included in the CMap online database to discover potential small molecule drugs for breast cancer treatment. The top 10 compounds with the highest enrichment scores were subsequently identified. Finally, we employed the R package “oncoPredict” to determine the IC50 values, which represent the half maximal inhibitory concentration, for widely utilized chemotherapy medications in the context of breast cancer [39]. Furthermore, we conducted a comprehensive evaluation to discern the differences in the effectiveness of chemotherapy drugs between groups classified as high risk and low risk.

### Cell culture

MCF10A cells were cultivated in DMEM/F-12 medium supplemented with 5% donor horse serum (DHS; Biological Industries, 04-004-1B), 1% penicillin/streptomycin, 20 ng/mL EGF (Sigma, E9644), 100 ng/mL cholera toxin (Sigma, C8052), and 10 µg/mL insulin (Sigma, I1882). SUM159 cells were cultured in DMEM/F12 medium supplemented with 10% FBS and 1% penicillin/streptomycin. MDA-MB-231 cells were grown in L15 medium supplemented with 10% FBS and 1% penicillin/streptomycin. HCC1954 cells were cultured in RPMI 1640 medium supplemented with 10% FBS and 1% penicillin/streptomycin. MCF-7 cells were grown in high-glucose DMEM supplemented with 10% foetal bovine serum (FBS) and 1% penicillin/streptomycin. The medium, FBS, and penicillin/streptomycin were acquired from Gibco (Thermo Fisher Scientific, Inc., Waltham, MA, USA). All cells were incubated at 37 °C with 5% CO2. The cell culture medium was changed every other day by replacing the old medium with fresh medium.

#### Cellular transfection

The lentivirus expressing OE-QARS1 was obtained from Bio-protocol (Xi’an, China). shRNAs targeting QARS1 were inserted into the pLKO.1 plasmid. MCF-7 cells and MDA-MB-231 cells were subsequently cotransfected with 4 µg of the respective plasmids, 30 µg of polyethylenimine, or 300 µl of Opti-MEM. The cells were harvested for downstream assays 48 h after transfection. All analyses were performed in triplicate. The sequences of the shRNAs can be found in Table S1.

#### Quantitative real-time PCR

Total RNA was extracted from breast cancer cells (SUM159, MDA-MB-231, HCC1954, and MCF-7) and a normal mammary epithelial cell line (MCF-10 A) using the RNAfast200 reagent (Fastagen Biotech; 220010). cDNA was subsequently synthesized from the RNA templates through reverse transcription with the StarScript II First-strand cDNA Synthesis Kit-II for qRT-PCR (GeneStar). The mRNA expression levels were quantified using SYBR-Green assays (GeneStar) on a Bio-Rad CFX96 instrument (Hercules). Data analysis was performed using the 2-ΔΔCT method, with β-actin used as an endogenous control. The primer sequences utilized in this study can be found in Table S1.

#### Western blot

Whole-cell lysates were obtained by treating the cells with RIPA lysis buffer, which consisted of a phosphatase inhibitor and a protease inhibitor (Roche, NJ, USA), using an ultrasonic crusher (Sonics & Materials, Inc., USA). The protein samples were subsequently incubated on ice for 20 min and then centrifuged at 4 °C for 20 min. The proteins located in the upper part of the supernatant were carefully collected and subjected to detection using a BCA Protein Assay Kit (Pierce; Thermo Fisher Scientific, Inc.). Next, 30 µg of proteins were separated via 10% SDS-PAGE gels and then transferred onto polyvinylidene fluoride (PVDF) membranes (Merck Millipore, Billerica, MA, USA). To minimize nonspecific binding, the membranes were blocked with 5% nonfat milk in TBST and subsequently incubated overnight at 4 °C with the primary antibody. Next, samples were incubated with a horseradish peroxidase-linked IgG secondary antibody (1:10000; Proteintech) at room temperature for 2 h. Following incubation, protein detection was performed using a chemiluminescence detection system (version 3.0; Bio-Rad Laboratories, Inc., Hercules, CA, USA). In this study, β-actin was utilized as a control. The primary antibodies against QARS1 (1:2000) and β-actin (1:10000) were purchased from Proteintech.

#### CCK-8 and colony assay

The CCK-8 assay was performed as follows: 10 µl of CCK-8 solution (YOBIBIO, U22-001 A) was added to each well of a 96-well plate at the specified time according to the manufacturer’s instructions. The plates were then incubated with 2 × 10^3 cells for the designated duration. After an additional 2 h of incubation at 37 °C, the absorbance was measured at 450 nm. For the cloning experiments, the cell colonies were cultivated in 6-well plates for 14 d. Crystal violet was used to stain the cell colonies.

All experiments were repeated three times.

#### ELISA assay

The intracellular content of S-adenosylmethionine (SAM) was measured using a Human SAM ELISA Kit (YOBIBIO, U96-3732E). The experimental approach employed the “sandwich method,” wherein the capture antibody was immobilized on the enzyme plate. This allowed for the capture of the target protein from the sample and standard solutions. Subsequently, the biotinylated detection antibody bound specifically to the target protein, whereas the SABC interacted with the biotinylated detection antibody. As a result, immune complexes were formed. The optical density (OD) value at 450 nm was then measured using a microplate reader. By constructing a standard curve, the concentration of the target protein within the cells could be determined.

### The human protein atlas (HPA)

The Human Protein Atlas (HPA) database is a comprehensive resource that utilizes proteomic, transcriptomic, and systems biology data to map human tissues, cells, and organs. It provides a wealth of information on protein expression patterns, subcellular localization, and functional annotations. Forty-four major normal tissues and some cancer tissues were subjected to immunohistochemical analysis. In our study, the expression of three hub genes in normal human breast and tumour tissues was confirmed using this database (http://www.proteinatlas.org/).

### Tumor immunotherapy gene expression resource (TIGER)

TIGER (http://tiger.canceromics.org/), an invaluable resource for studying tumour immunotherapy gene expression, comprises a comprehensive collection of data [40]. This dataset includes bulk transcriptome data from 1508 tumour samples, providing insights into clinical immunotherapy outcomes. Additionally, TIGER includes 11,057 tumour/normal samples, which, although lacking clinical immunotherapy outcome annotations, contribute to a broader understanding of tumour biology. Furthermore, this resource offers single-cell transcriptome data, detailing the gene expression profiles of 2,116,945 immune cells across 655 samples.

### Statistics analysis

Statistical analyses were performed using R (version 4.3.2) and GraphPad Prism (version 9.0.1). The data used for the initial analysis were FPKM data. However, to eliminate technical variations between samples and make the data more comparable, the FPKM expression data were standardized through log2 transformation. Comparisons of continuous variables among different groups were performed via Student’s t test or the Wilcoxon rank-sum test to compare two groups based on the distribution of the data. The Kruskal-Wallis test was used for multigroup comparisons. A two-tailed *P* < 0.05 was considered statistically significant.

## Result

### Identification of two molecular subtypes based on AAMRGs

**Fig. 2:**
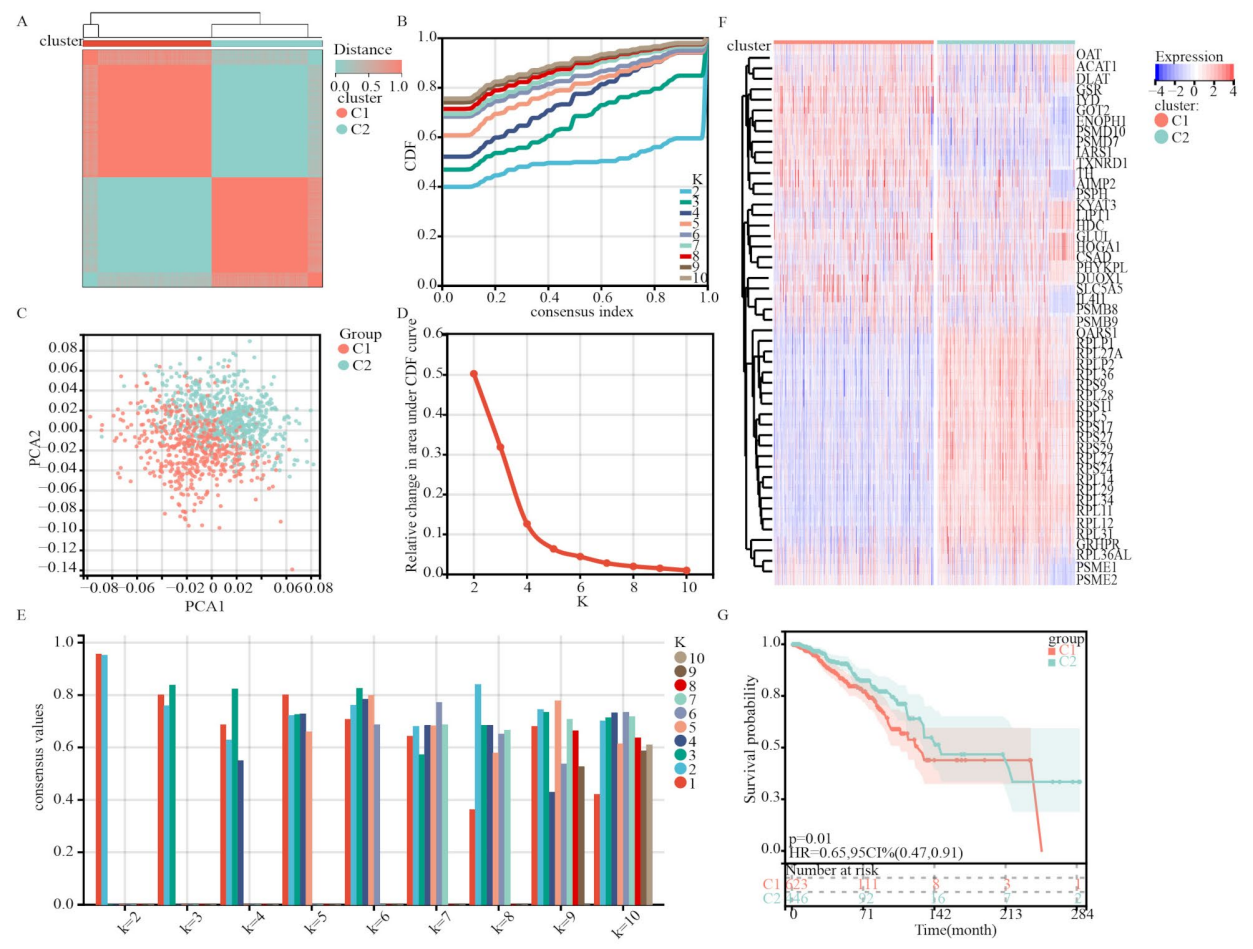
Consensus cluster. **A–E**. K = 2 was identified as the optimal value for consensus clustering. **C**. Principal component analysis between the two subgroups. **F**. Heatmap visualizing the expression of amino acid metabolism genes in the two subgroups. **G**. Survival curves of the patients in the two subgroups

A consensus clustering approach was employed to categorize the patients with breast cancer in the training cohort into subgroups based on 50 prognostic genes derived from univariate Cox analysis. With the aim of maximizing the separation between groups, considering the gradual decrease in delta, as determined by CDF trend evaluation and intragroup consistency assessment, we ultimately selected K = 2 to determine the optimal clustering stability (Fig. 2A-E). Cluster 1 included 651 patients, whereas Cluster 2 included 560 patients. By visualizing AAMRGs through a heatmap displaying their expression levels (Fig. 2F), noticeable disparities in gene expression were observed between Cluster 1 and Cluster 2. The results of the principal component analysis further confirmed the distinctions between the two clusters (Fig. 2C). Importantly, patients in Cluster 2 exhibited significantly improved overall survival compared with those in Cluster 1 (*P* = 0.01, HR = 0.65, 95%CI = 0.47–0.91; Fig. 2G). These findings demonstrated that AAMRGs could be used to stratify patients with breast cancer effectively into two molecular subtypes characterized by distinct overall survival rates.

### Immune infiltration status in the two molecular subtypes

**Fig. 3:**
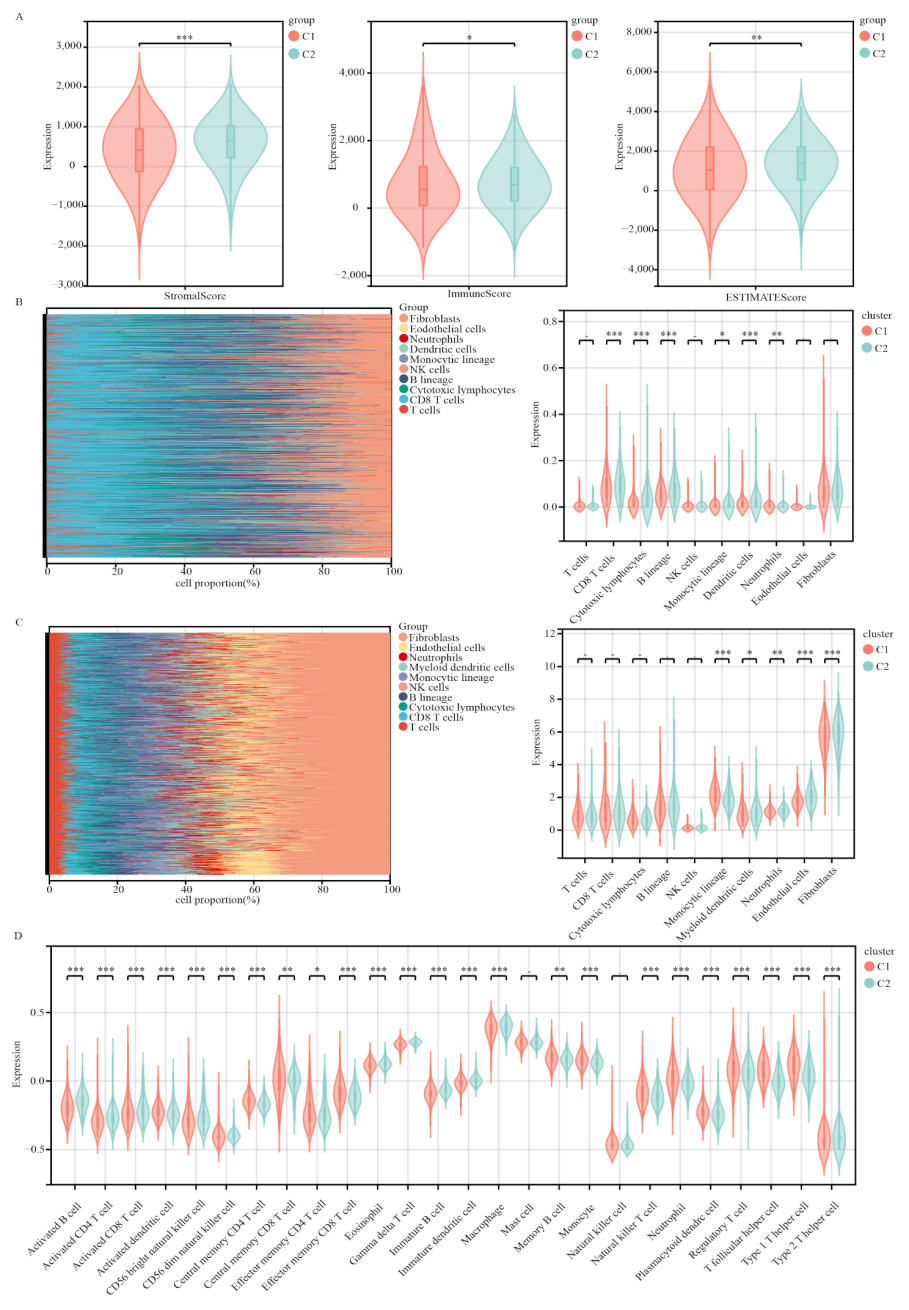
Immune analyses of the two clustered subgroups. **(A)** Stromal score, immune score and ESTIMATE score calculated via the ESTIMATE algorithm. **(B)** Abundance of ten immune-filtrating cells evaluated by the CIBERSORT algorithm. **(C)** Abundance of ten immune-filtrating cells evaluated by the MCP-counter algorithm. **(D)** Violin plot depicting the enrichment levels of 26 immune-related cell types evaluated via the ssGSEA algorithm. *p < 0.05; **p < 0.01; ***p < 0.001

We conducted immune analyses to explore the immune differences between the two molecular subtypes. According to the ESTIMATE algorithm, patients with breast cancer in Cluster 2 presented higher immune scores (*P* < 0.05), ESTIMATE scores (*P* < 0.01), and stromal scores (*P* < 0.001) than did those in Cluster 1 (Fig. 3A). Moreover, the CIBERSORT algorithm revealed that patients in Cluster 2 had significantly more B cells (*P* < 0.001), cytotoxic lymphocytes (*P* < 0.001), dendritic cells (*P* < 0.001), monocytic lineage cells (*P* < 0.05), neutrophils (*P* < 0.01), and CD8 + T cells (*P* < 0.001) than did those in Cluster 1. However, no statistical difference in the amount of fibroblasts was observed (*P* > 0.05; Fig. 3B). The MCP-counter algorithm also revealed differences in the amounts of dendritic cells (*P* < 0.001), monocytic lineage cells (*P* < 0.01), and neutrophils (*P* < 0.05) between the two clusters (Fig. 3C). The ssGSEA algorithm (Fig. 3D) revealed significant differences in the amounts of the 26 immune cells between the two clusters, with the exception of mast cells and natural killer cells. These findings underscore the substantial disparities in the immune profiles exhibited by the two molecular subtypes.

### DEG and functional analyses

**Fig. 4:**
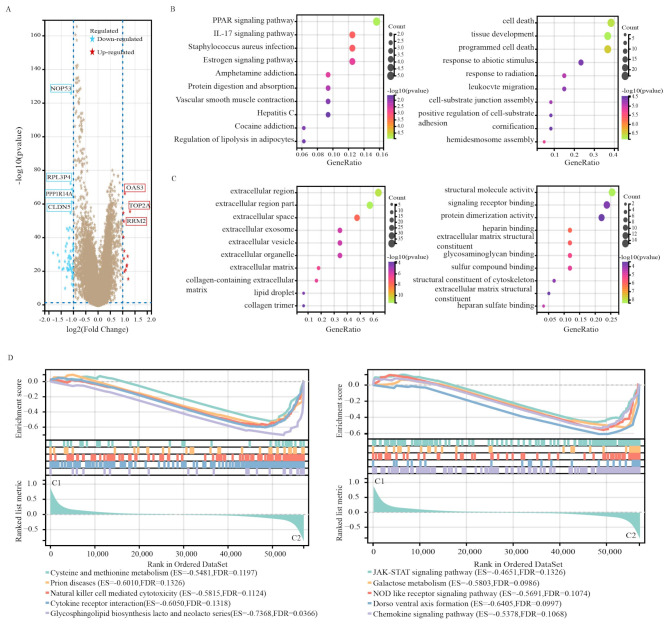
Differentially expressed gene analysis and functional analyses. **(A)** Volcano plot showing the DEGs between the two subgroups. **(B)** Bubble diagram showing the signalling pathways enriched by Kyoto Encyclopedia of Genes and Genomes (KEGG) analysis. **(C)** Gene Ontology (GO) analysis. **(D)** GSEA plots visualizing the results of GSEA

Differential expression analysis was subsequently conducted to identify DEGs between the two clusters, followed by functional analyses to explore the underlying signalling mechanisms. A total of 65 DEGs were detected, with 52 genes downregulated and 13 genes upregulated in Cluster 2 compared with Cluster 1 (Fig. 4A). KEGG enrichment analysis revealed enrichment in pathways related to PPAR signalling, IL-17 signalling, and *Staphylococcus aureus* infection (Fig. 4B, Supplementary Fig. 1). Similarly, GO enrichment analysis revealed significant enrichment of DEGs in pathways related to cell death, tissue development, and response to abiotic stimuli (Fig. 4C, Supplementary Fig. 1). Additionally, key molecular functions and cellular components were enriched. To further investigate the relationship between the enriched pathways and the prognosis of patients with breast cancer, GSEA was performed to assess the relative expression differences in pathways between the two clusters. The analysis revealed lower expression of genes involved in pathways related to cysteine and methionine metabolism, prion diseases, natural killer cell-mediated cytotoxicity, and cytokine receptor interaction in Cluster 1 (Fig. 4D). These findings collectively suggest an association between the expression of AAMRGs and immune and metabolic pathways, which may contribute to the unfavourable prognosis observed in patients with breast cancer.

### Development of risk model based on AAMRGs in the training cohort

**Fig. 5:**
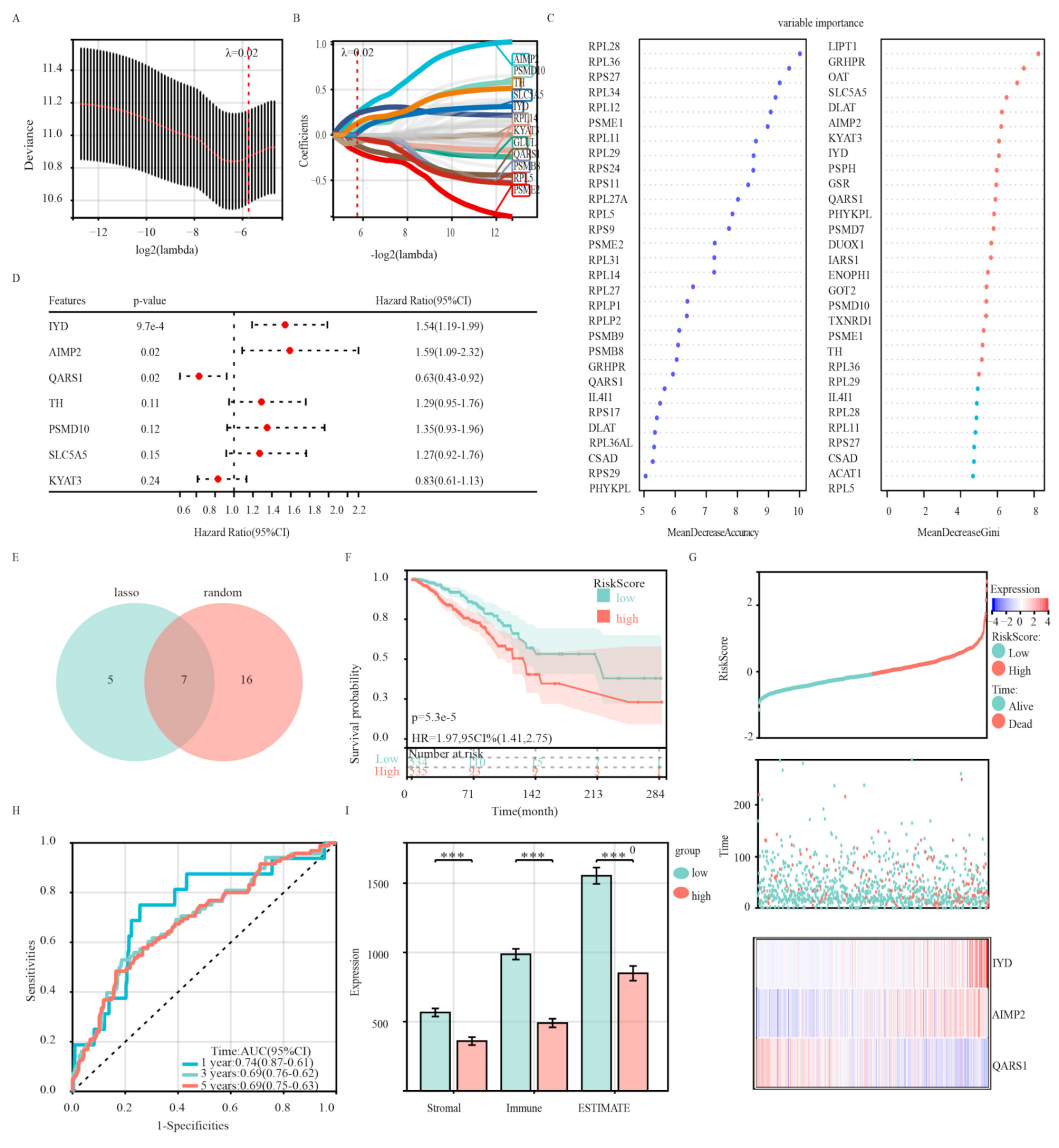
Construction of the risk model in the training cohort. **A–B**. LASSO analysis with minimal lambda. **C**. Hub genes were screened via the random forest (RF) algorithm. **D**. Multivariate Cox regression analysis. **E**. Venn diagram showing two genes common to the LASSO and RF algorithms that were identified as hub genes for amino acid metabolism in breast cancer. **F**. Survival curves of the patients with BRCA in the two groups. **G**. Distribution of the survival status and risk score of patients with BRCA in the high- and low-risk groups. **H**. ROC curve of the risk model. **I**. Stromal score, immune score and ESTIMATE score calculated via the ESTIMATE algorithm in the two groups. *p < 0.05; **p < 0.01; ***p < 0.001

A risk model was developed to assess the prognostic value of AAMRGs in breast cancer. LASSO analysis was employed to identify potential genes for constructing the risk model, resulting in the selection of 12 genes with the best lambda values (Fig. 5A, B). To further refine the pool of diagnostic biomarkers, a random forest (RF) machine learning algorithm was employed to rank candidate genes based on their variable importance, and genes with MeanDecreaseGini > 5 were extracted (Fig. 5C). Interestingly, the overlap of the 12 candidate genes from LASSO and the 23 potential genes from RF led to the identification of 7 core genes shared by both subsets (Fig. 5E). Based on the genes identified by the machine learning algorithm, multivariate Cox analysis identified three genes, namely, AIMP2, IYD, and QARS1, for constructing the risk model. Kaplan-Meier analysis in combination with the risk model demonstrated that all three genes serve as independent prognostic markers for patients with breast cancer (Fig. 5D, Supplementary Fig. 2). The established risk model successfully stratified patients with breast cancer into high-risk and low-risk groups (Fig. 5G), with the low-risk group exhibiting higher overall survival rates than the high-risk group (Fig. 5F). ROC curve analysis of the risk model (Fig. 5H) demonstrated favourable diagnostic performance. Time-dependent ROC analysis revealed that the risk model exhibited accurate predictive ability over a 5-y period, with area under the ROC curve (AUC) values of 0.74, 0.69, and 0.69 at 1, 3, and 5 y, respectively (Fig. 5H). Finally, the ESTIMATE algorithm was employed to assess the differences in the tumour immune microenvironment between the two risk groups. Compared with the high-risk group, the low-risk group presented significantly higher stromal scores (*P* < 0.001), immune scores (*P* < 0.001), and ESTIMATE scores (*P* < 0.001) (Fig. 5I). These findings suggest that the constructed risk model has potential for prognostic prediction in patients with breast cancer and is significantly associated with the TIME.

### Construction and calibration of an integrated nomogram

**Fig. 6:**
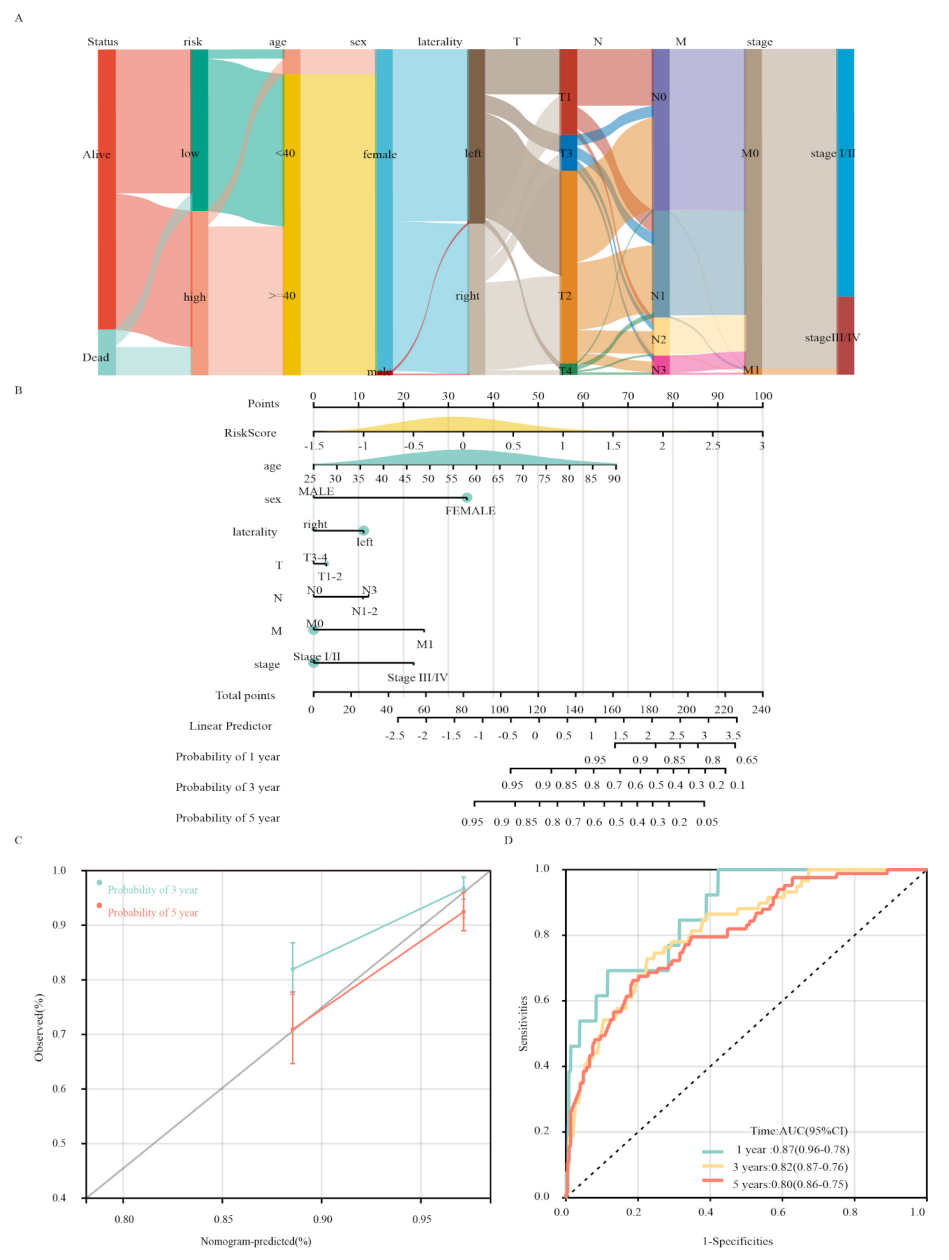
Construction and calibration of the nomogram. **(A)** Sankey plot of the distribution of clinical characteristics. **(B)** Nomogram combining clinical features and the risk score. **(C)** Calibration curve of the model. **(D)** ROC curve of the model

To increase the accuracy of prognosis prediction in patients with breast cancer, a nomogram that integrated the risk models and clinical characteristics was developed. The distribution of clinical features is displayed in Fig. 6A, while the constructed nomogram is depicted in Fig. 6B. Each risk score and clinical feature were assigned a specific score based on its contribution to breast cancer prognosis. The nomogram exhibited acceptable accuracy, as evidenced by the C-index values and model diagnostics of the calibration curve (Fig. 6C and D). In the training cohort, the 1-, 3-, and 5-y C-index values of the nomogram were 0.87 (95%CI = 0.96–0.78), 0.82 (95%CI = 0.87–0.76), and 0.80 (95%CI = 0.86–0.75), respectively. Similar results were observed in the validation cohort (Supplementary Fig. 3). These findings indicated that the comprehensive nomogram could accurately predict the prognosis of patients with breast cancer. Furthermore, we explored the associations between risk scores and clinical characteristics and assessed the independence of the constructed risk model through subgroup analysis and regression analysis. The results showed that the constructed risk model had good independence in predicting breast cancer prognosis (Supplementary Fig. 4A, 4B).

### Drug susceptibility analysis

**Fig. 7:**
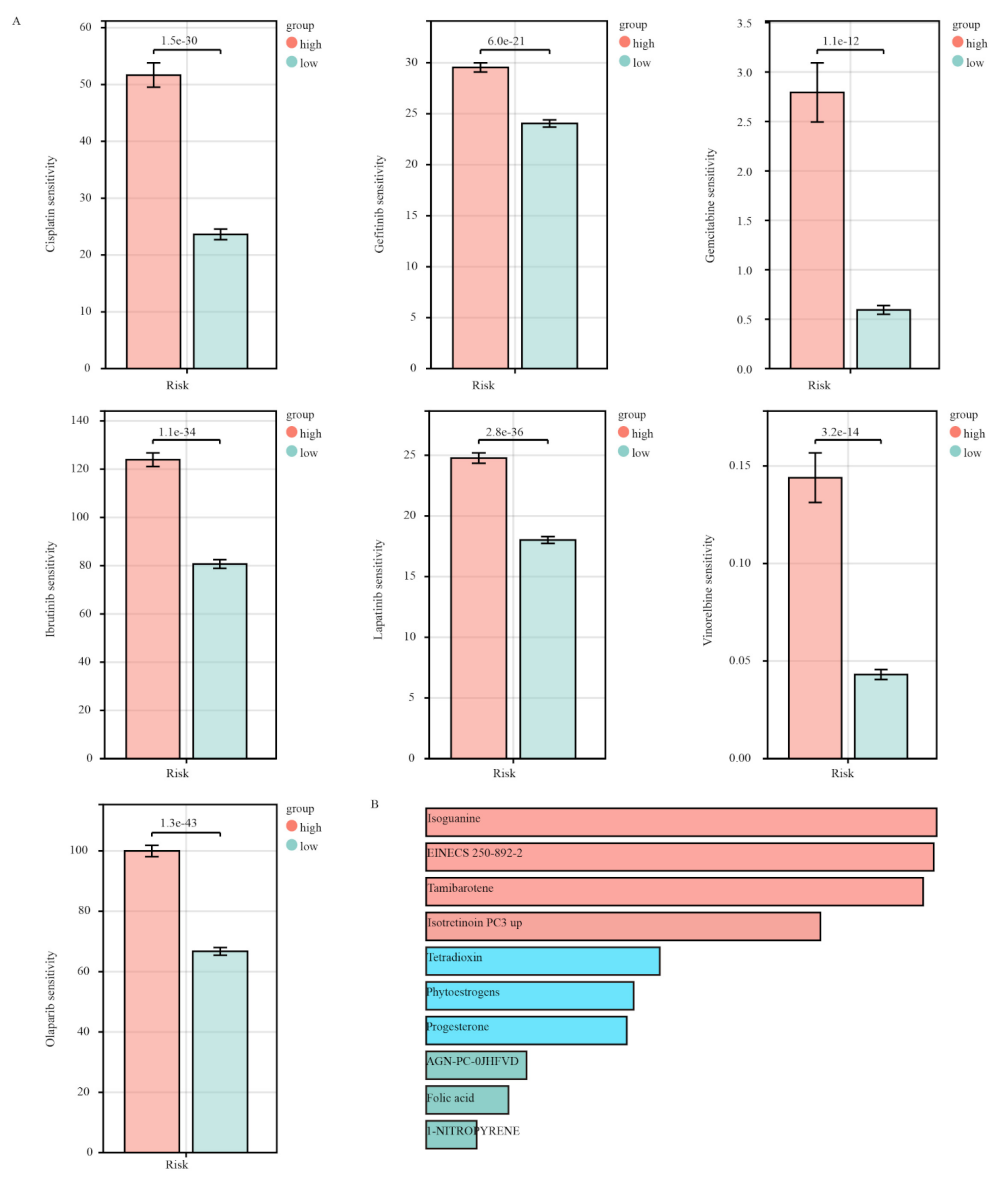
Drug sensitivity analysis. **(A)** Model-based drug sensitivity analysis. **(B)** Screening for small molecule compounds using the CMap database

We evaluated the sensitivity to common treatments of patients with breast cancer in the high-risk and low-risk groups. Figure 7A shows that most therapeutic drugs presented lower IC50 values in the low-risk group. These drugs include cisplatin, gefitinib, gemcitabine, ibrutinib, lapatinib, and vinorelbine, among others, suggesting that patients in the low-risk group may have a better response to these treatments. Furthermore, we identified 231 genes that were highly expressed in the high-risk group and utilized the CMap database to screen for small molecule compounds that could reverse the expression of these disease-associated genes. After an extensive investigation, we identified the top 10 compounds with the highest scores. These compounds include isoguanine, EINECS 250-892-2, tamibarotene, isotretinoin PC3 UP, tetradioxin, phytoestrogens, progesterone, AGN-PC-0JHFVD, folic acid, and 1-nitropyrene (Fig. 7B). The scores of these specific compounds can be found in Supplementary Table 2.

### Validation of the expression levels of the three AAMRGs in the risk model

Analysis of breast cancer gene expression data from the TCGA database revealed that the expression of AIMP2 and IYD was elevated in breast cancer tissues, whereas the expression of QARS1 was elevated in normal tissues (Supplementary Fig. 5A). Additionally, data on the immunohistochemical expression of these three genes were extracted from the HPA database, and the analysis results were consistent with the findings from the TCGA data (Supplementary Fig. 5B).

**Fig. 8:**
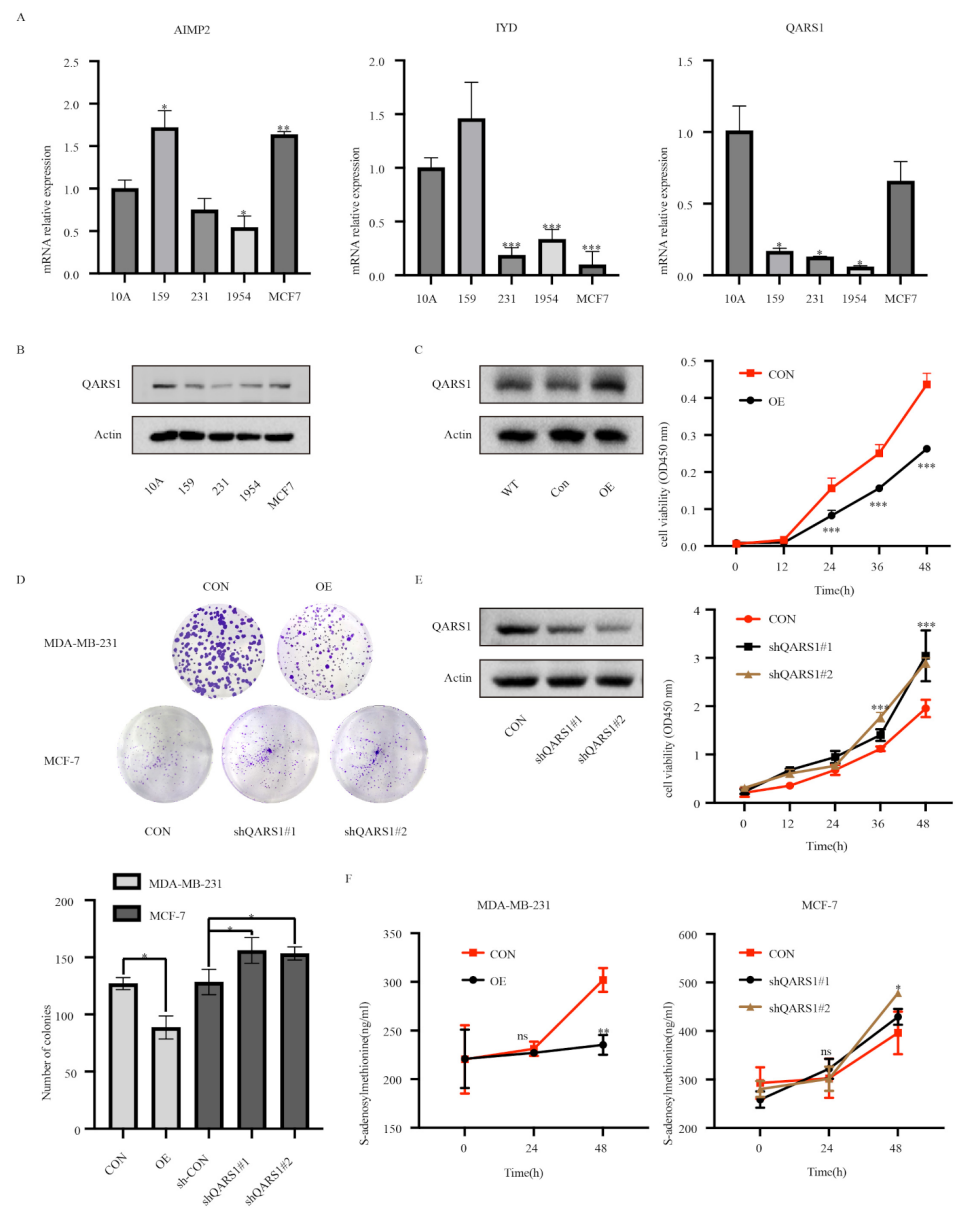
Validation of the expression levels of the three AAMRGs in the risk model and mechanism exploration. **(A)** mRNA expression of three prognosis-related AAMRGs in BRCA cells and normal mammary epithelial cells measured via qRT-PCR. **(B)** Protein expression of QARS1 in BRCA cells and normal breast epithelial cells measured via Western blotting. **C-E**. Overexpression or knockdown of the QARS1 gene in MDA-MB-231 and MCF-7 cell lines, and cell proliferation evaluated through CCK8 and colony-formation assay. **F**. Determination of SAM contents under different treatment conditions. *p < 0.05; **p < 0.01; ***p < 0.001

To gain further insight into the expression of the AIMP2, QARS1, and IYD genes in breast cancer, qRT-PCR was used to measure their mRNA expression levels. Compared with that in normal breast cells, AIMP2 expression was high in breast cancer cells, whereas QARS1 expression was low in breast cancer cells; the expression of IYD was not clear (Fig. 8A). Furthermore, we examined single-cell transcriptome data for three genes in breast cancer using the TIGER database. The findings revealed notable enrichment of AIMP2 and IYD in tumour cells, whereas QARS1 was enriched in immune cells (Supplementary Fig. 6).

### QARS1 regulates breast cancer cell proliferation by inhibiting methionine metabolism

Finally, we measured the protein-level expression of the QARS1 gene, which exhibited consistent results in informatics analysis and qRT-PCR verification. The results were consistent with the conclusions drawn from the qRT-PCR analysis (Fig. 8B). Additionally, we conducted experiments in which we either overexpressed or knocked down the QARS1 gene in the MDA-MB-231 and MCF-7 cell lines. We observed changes in their respective cell proliferation capacities. The results demonstrated that the proliferation ability of MDA-MB-231 cells overexpressing the QARS1 gene was inhibited compared to that of the control cells. Conversely, the proliferation ability of MCF-7 cells with a knocked down QARS1 gene was greater than that of the control cells (Fig. 8C-E). Moreover, we assessed the cellular content of S-adenosylmethionine (SAM), a crucial intermediate product of methionine metabolism. Notably, we discovered that the overexpression of the QARS1 gene led to a decrease in SAM production, whereas its knockdown resulted in an increase (Fig. 8F). These findings suggest that the QARS1 gene may promote the proliferation of breast cancer cells by participating in the regulation of methionine metabolism.

## Discussion

Breast cancer encompasses distinct subtypes characterized by unique biological and clinical features. Key molecular biomarkers, such as the oestrogen receptor (ER), progesterone receptor (PR), and human epidermal growth factor receptor 2 (HER-2), have been thoroughly investigated and are well established in the field [41]. Advancements in the field of metabolomics have led to increased research on the phenotype of malignant invasive breast cancer [42, 43]. Notably, genes involved in glucose metabolism and fatty acid metabolism have shown promising potential as prognostic markers for OS in invasive breast cancer [44, 45]. Additionally, amino acid metabolism is a source of energy for tumour cells. The exploration of the functional role of genes involved in amino acid metabolism in breast cancer is crucial for determining patient prognosis and guiding therapeutic decisions.

In this study, we utilized the expression matrix of AAMRGs from patients with breast cancer to identify two distinct molecular subgroups through consensus clustering. These subgroups exhibited significant differences in overall survival. Next, we conducted comprehensive immunological and functional analyses to explore the role of amino acid metabolism in breast cancer. We discovered two molecular isoforms with markedly different amino acid metabolic profiles. Immune analysis indicated that patients with poor prognoses had lower immune statuses and scores, including lower ESTIMATE scores, than did those with more favourable outcomes. Further functional analysis revealed a correlation between AAMRG expression and various immune and metabolic pathways. Additionally, we developed a prognostic risk model that integrates AAMRG expression with clinical characteristics, enabling accurate predictions of breast cancer prognosis. Our findings have the potential to advance the development of targeted therapies for breast cancer and assist clinicians in making more informed treatment decisions.

Increasing evidence suggests that immune infiltration within the TIME is critical for predicting the prognosis of patients with BRCA, and abnormalities in the metabolic status of tumour cells may also lead to changes in the TIME [46]. The ESTIMATE algorithm is a computational approach used to estimate the relative proportions of immune cells and stromal cells within tumours using gene expression data [31]. The immune score derived from this algorithm provides a quantitative assessment of the immune components present in tumour samples. Consequently, we utilized the ESTIMATE algorithm to evaluate the TIME between two groups. Our results consistently demonstrated that patients with a more favourable prognosis had higher immune scores, which aligns with the findings of previous studies. Additionally, we applied the CIBERSORT and MCP-counter algorithms to evaluate the immune status across molecular subpopulations [32, 33]. This allowed us to further validate the observed immune differences between the various subtypes. Moreover, we conducted ssGSEA to explore the associations between different immune-related cell types and the expression groups of amino acid metabolism genes. These findings revealed that the expression of AAMRGs was not only correlated with the immune microenvironment but also significantly associated with the prognosis of patients with breast cancer. These findings demonstrated the potential involvement of AAMRGs in modulating the immune microenvironment, consequently resulting in divergent patient prognoses.

To better understand the underlying mechanisms, we conducted functional analyses of the two subgroups. Results from KEGG and GO analyses indicated that amino acid metabolism genes potentially impact cellular immunity through pathways such as the PPAR signalling pathway and the IL-17 signalling pathway. Previous studies have shown that arginine protein methyltransferase (PRMT1) modulates macrophage differentiation by regulating PPARγ activity, thereby modulating immune responses. Additionally, the tryptophan catabolite 3-HKA has been shown to diminish IL-17 production, thereby inhibiting the expression of effector CD8 + T cells [47, 48]. GSEA provided insights into the expression patterns of gene sets, revealing lower expression in Cluster 1 for gene sets related to cysteine and methionine metabolism, prion diseases, natural killer cell-mediated cytotoxicity, and cytokine receptor interactions. These findings underscore the connection between AAMRG expression and both immune and metabolic pathways.

To further substantiate the impact of amino acid metabolism disturbances on breast cancer and explore the prognostic significance of amino acid metabolism-related genes (AAMRGs) in patients with breast cancer, we formulated a prognostic risk model based on AAMRGs and subsequently validated it within the validation cohort. This model incorporated three genes previously linked to tumour progression. The protein encoded by AIMP2 is an integral component of the aminoacyl-tRNA synthetase complex, which is capable of eliciting apoptosis by facilitating the degradation of TNF receptor-associated factor 2 (TRAF2) and the activation of p53 following DNA damage [49]. Furthermore, compelling evidence suggests that AIMP2 plays a pivotal role in governing the intestinal Wnt/β-catenin signalling pathway and tumorigenesis [50]. The expression pattern and functional role of IYD in cancers are not known. Studies have shown that mutations in QARS1 (encoding human glutaminyl-tRNA synthetase) are associated with epilepsy, developmental decline, progressive microcephaly, and brain atrophy [51]. One of the key functions of this gene is its capacity to inhibit apoptosis by negatively regulating apoptosis signal-regulated kinase 1 (ASK1) [52]. Notably, certain scholars have reported that QARS1 gene expression is lower in tumours than in normal breast tissue [53, 54]. Their analysis revealed that among the tRNA synthetases exhibiting greater than 50% depletion in human cell lines and TCGA analysis, only QARS1 and LARS displayed significant reductions [53]. Consequently, inhibition of QARS1 may occur during breast tumorigenesis, which aligns with the findings of our study, and it could impact the proliferation of breast tumour cells by modulating methionine metabolism.

Survival analysis demonstrated that the established risk model accurately predicted outcomes for patients with breast cancer in both the training and validation cohorts, revealing a correlation between lower stromal and immune scores and reduced survival rates. Additionally, independent and subgroup analyses confirmed the prognostic ability of the risk model based on amino acid metabolism-related genes (AAMRGs) in breast cancer. We also developed a nomogram that integrated risk scores with clinical characteristics. These findings underscore the predictive significance of AAMRGs in breast cancer and their association with altered amino acid metabolism and the tumour microenvironment. Compared with prior studies, our research presents distinct advantages. First, we concentrated specifically on amino acid metabolism genes in patients with breast cancer, facilitating the identification of two molecular subgroups characterized by markedly different prognoses and immune statuses through consensus clustering. Second, we investigated the biological mechanisms underlying breast cancer based on these clustering outcomes, thereby partially elucidating the underlying processes. Third, we employed machine learning algorithms and bioinformatics techniques to create a nomogram that integrates risk scores and clinical characteristics, demonstrating significant improvements in survival prediction. Moreover, we examined the impact of QARS1 expression on breast tumours. Finally, we incorporated three GEO datasets as validation cohorts, encompassing a substantially larger sample size than did previous studies. This work not only enhances the theoretical understanding of breast cancer but also supports the development of targeted therapies and the optimization of treatment strategies.

This study had several limitations. Primarily, as a retrospective study, it was subject to inherent selection bias. Additionally, the number of breast cancer cases in the database was limited, and some important clinical information, such as information concerning surgery and neoadjuvant treatment, which are important factors affecting the prognosis of patients with breast cancer, was lacking. Moreover, the results require further validation through external clinical datasets and experimental studies.

## Conclusion

In conclusion, we identified two distinct molecular subtypes of breast cancer based on AAMRGs, revealing unique biological and immunological characteristics. To enhance clinical applicability, we developed a robust nomogram that integrates risk scores from AAMRGs with clinical features and uses machine learning and bioinformatics to improve risk stratification and prognostic accuracy. These findings establish a foundation for personalized treatment strategies, as the identified subtypes and nomogram can guide targeted therapy development and optimize decision-making for patients with breast cancer. Additionally, our study underscores the potential of AAMRGs as biomarkers for prognostic evaluation and immune modulation, paving the way for innovative therapeutic interventions to improve patient outcomes in clinical practice.

## Electronic supplementary material

Below is the link to the electronic supplementary material.

Supplementary Material 1

Supplementary Material 2

Supplementary Material 3

## Data Availability

The TCGA datasets presented in this study can be found in online repositories [https://portal.gdc.cancer.gov/]. The GEO datasets generated and/or analysed during the current study are available in this repository, [www.ncbi.nlm.nih.gov/geo/].
