# Supplementary material for: Molecular biomarkers for the prognosis of breast cancer: role of amino acid metabolism genes
Source: J Physiol Biochem. 2025 Jun 10;81(2):441–57. doi: 10.1007/s13105-025-01088-5 (PMC12279611; doi:10.1007/s13105-025-01088-5)
Supplement: Supplementary file 1 — Supplementary Material 1 [file 13105_2025_1088_MOESM1_ESM.docx]

Supplementary Material

# Supplementary Figures and Tables


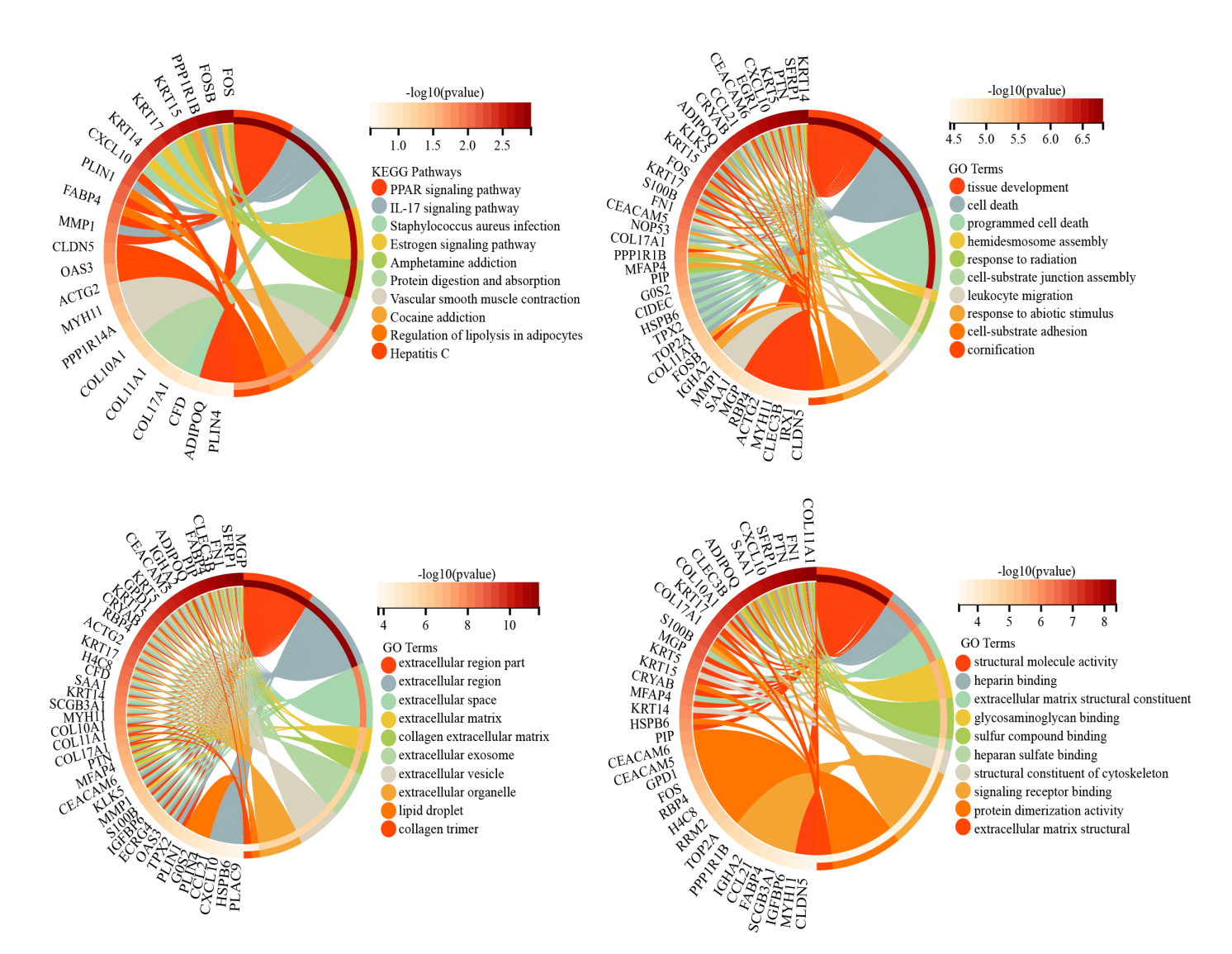


**Supplementary Figure 1.** Circle plot visualizing the biological processes enriched by Kyoto Encyclopedia of Genes and Genomes (KEGG) analysis and gene ontology (GO) analysis.


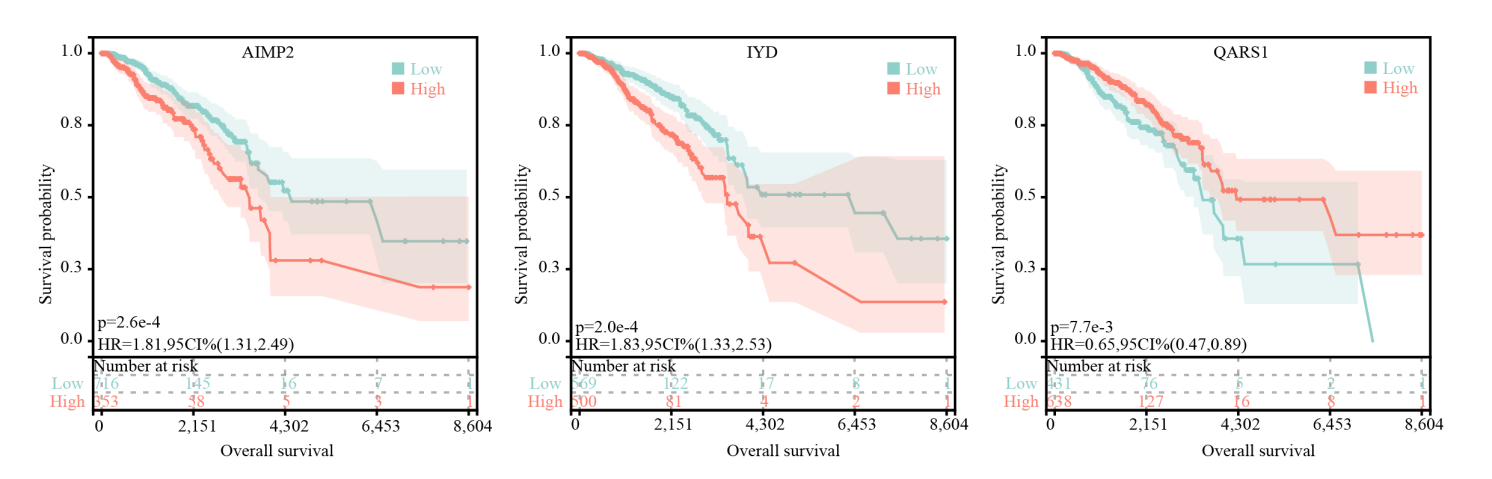


**Supplementary Figure 2.** Survival curves regarding expression of three AAMRGs associated with breast cancer prognosis.


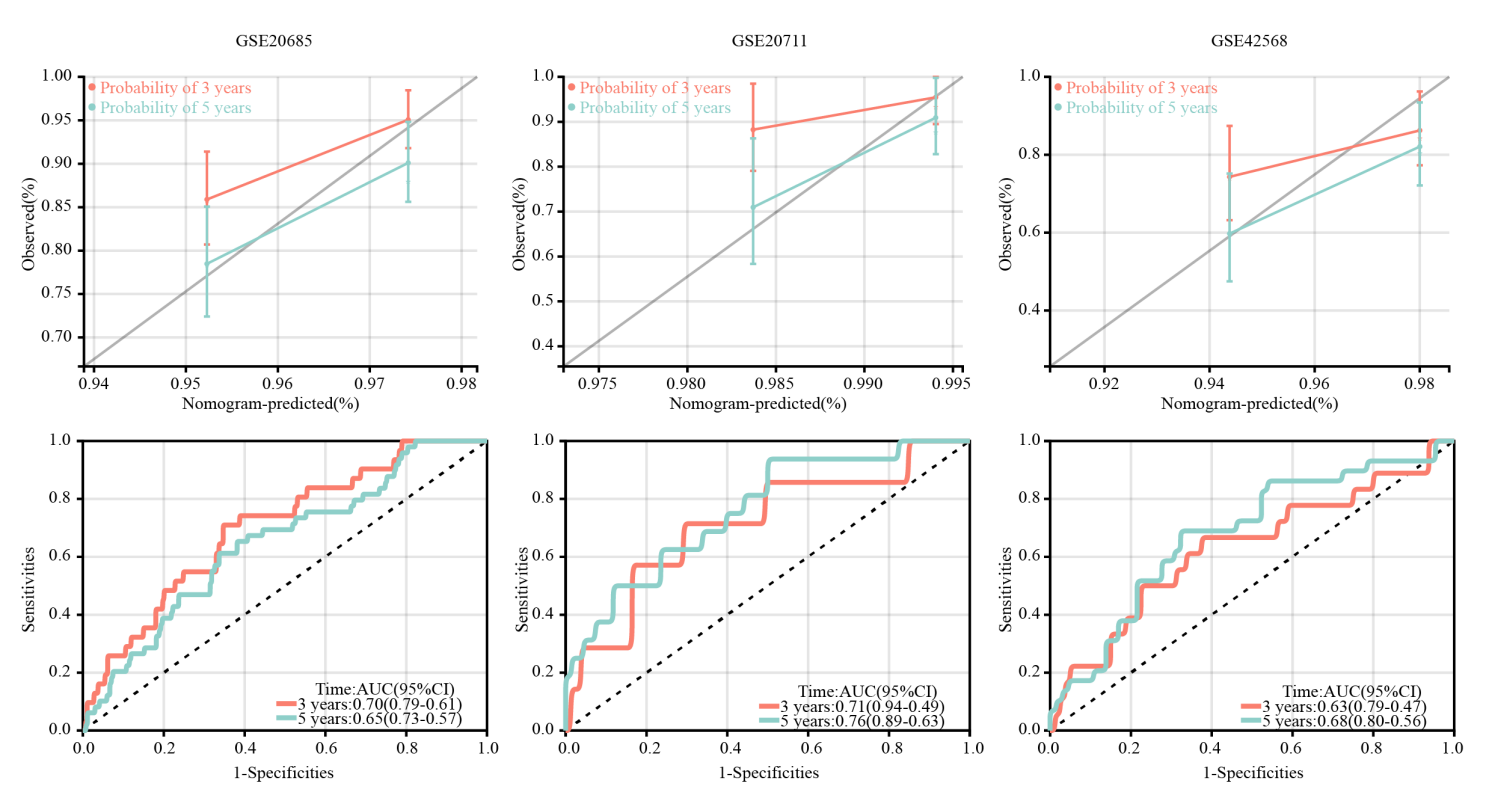


**Supplementary Figure 3.** Model validation in GEO datasets included GSE20685, GSE20711 and GSE42568.


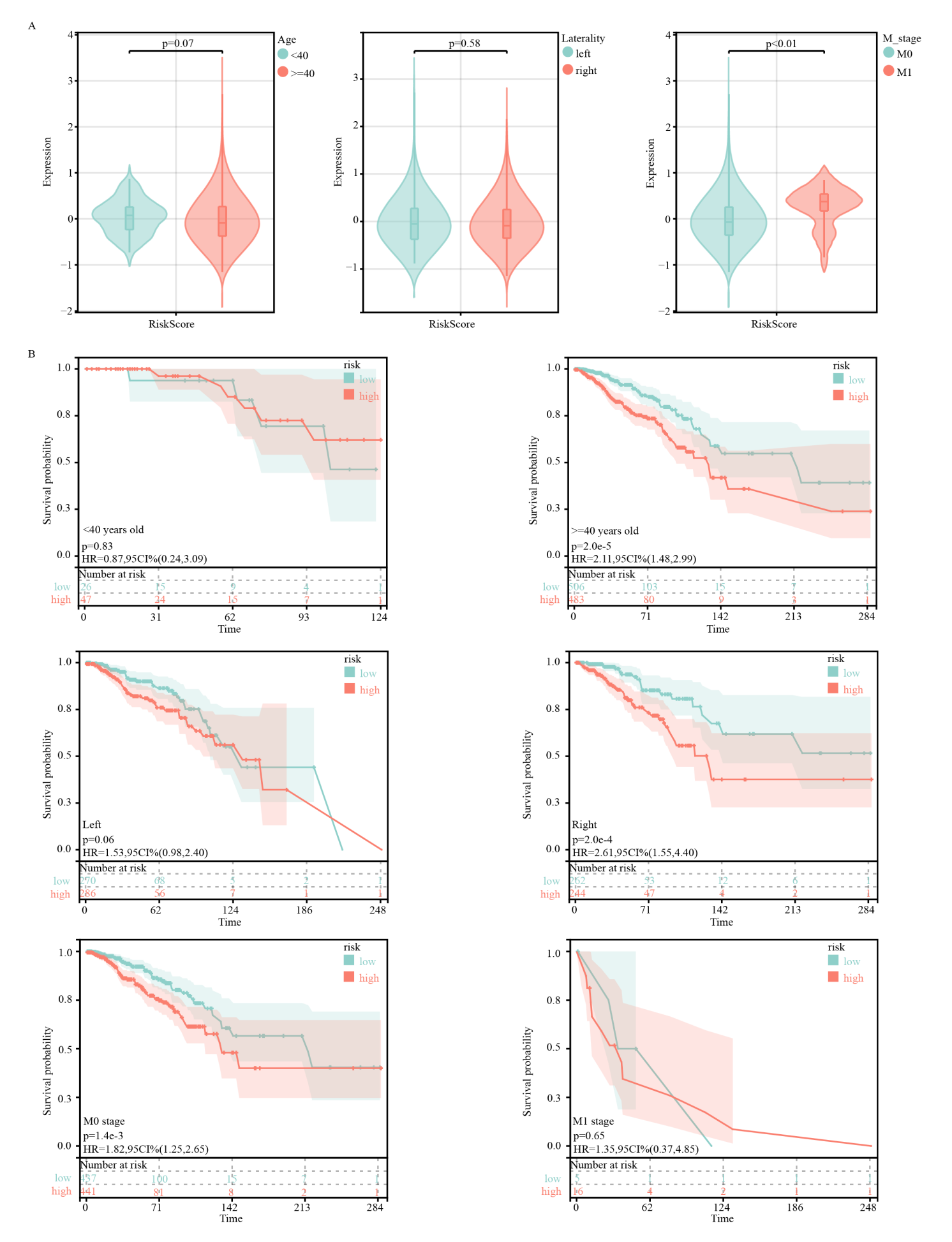


**Supplementary Figure 4.** Association of risk score and clinical characteristics. (A) No significant difference was identified in patients with different age and laterality, while significant difference was identified in patients with different metastasis status. (B) Survival curve of patients regrouped according to age, laterality, and metastasis.


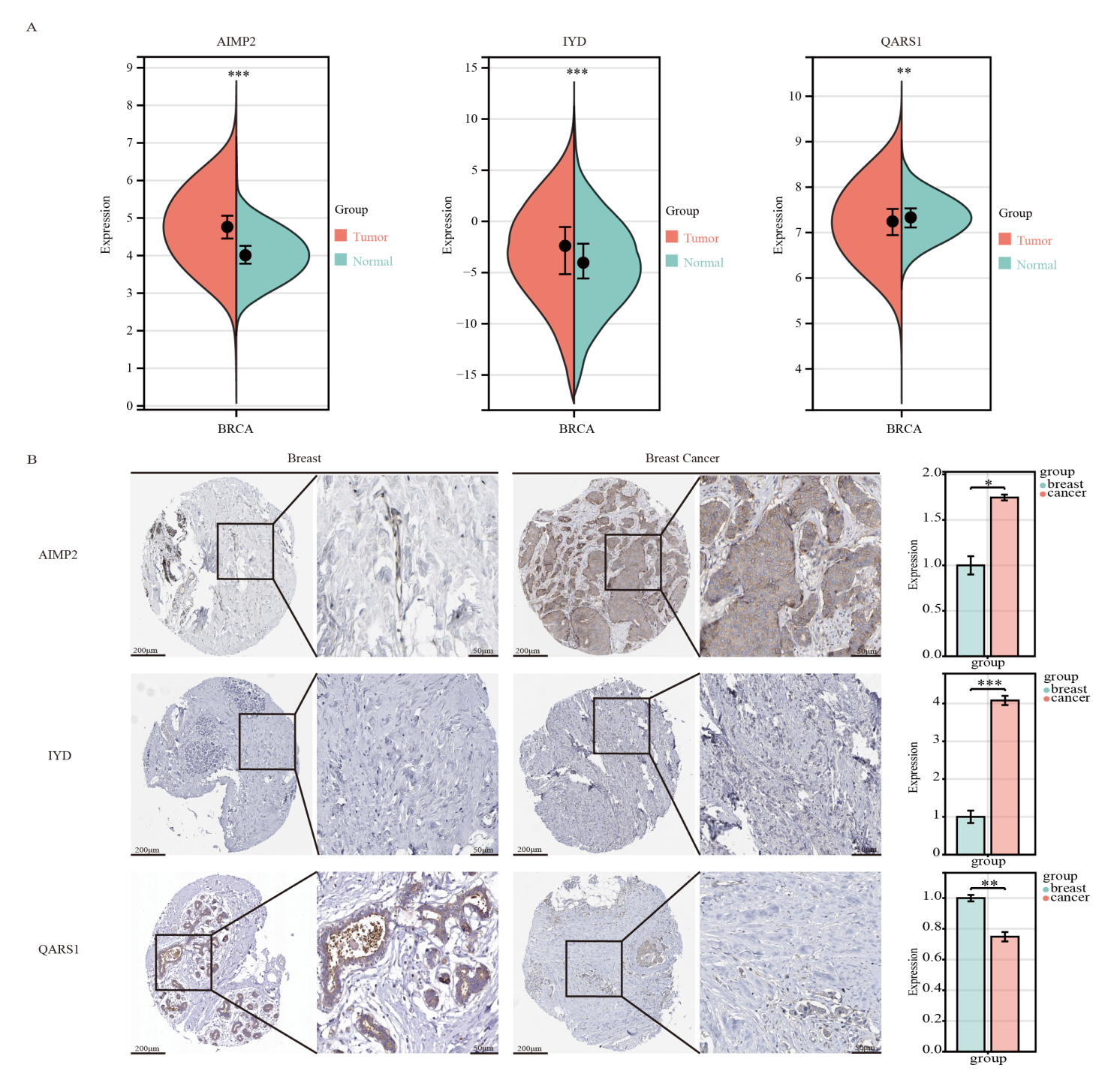


**Supplementary Figure 5.** Validation of the expression levels of the three AAMRGs. (A) Expression of three AAMRGs in breast cancer and normal tissues. (B) Immunohistochemical expression of three genes in breast cancer and normal tissues based on HPA database.


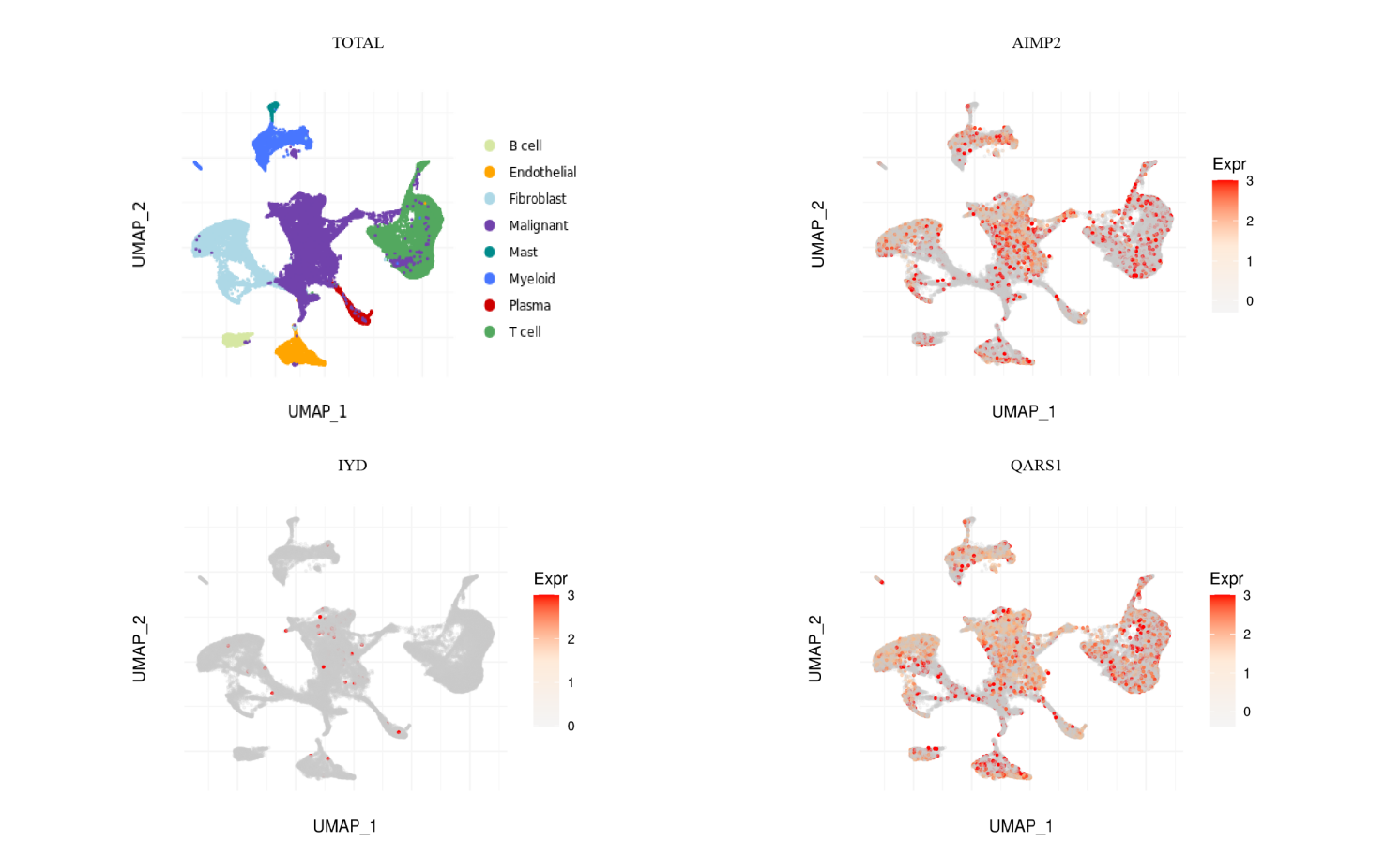


**Supplementary Figure 6.** The single-cell transcriptome data for three AAMRGs in breast cancer using the TIGER database.

| Table S1 The sequences of shRNA and primers for real-time PCR assays. | |
| --- | --- |
| Gene | Primer sequence (5′-3′) |
| shQARS1#1 | F: GCTAGGCTACACACCTTACAA |
|  | R: TTGTAAGGTGTGTAGCCTAGC |
| shQARS1#2 | F: CCCTTCGACAAGTTCCAGTTT |
|  | R: AAACTGGAACTTGTCGAAGGG |
| AIMP2 | F: CATGCCGATGTACCAGGAAGA |
|  | R: TGGTGGTTAAAGTCGTGGGC |
| IYD | F: CACCAAGCAGAAGAAGATGCTG |
|  | R: GAACCTGACTGACCGTCTCT |
| QARS1 | F: AGTTTCCGGTGTCTCTGCAA |
|  | R: AGGGTCTGCTGAGCCTGA |
| β-actin | F: AGCCTCGCCTTTGCCG |
|  | R: CTCGTCGCCCACATAGGAAT |

| Table S2 Identification of candidate small-molecular compounds for high risk patients. | | | | |  |
| --- | --- | --- | --- | --- | --- |
| Term | P-value | Adjusted P-value | Odds Ratio | Combined Score | |
| Isoguanine BOSS | 1.43E-07 | 9.33E-05 | 6.52275757 | 102.7842949 | |
| EINECS 250-892-2 | 1.47E-07 | 9.33E-05 | 5.99693126 | 94.34488007 | |
| Tamibarotene | 1.62E-07 | 9.33E-05 | 3.814076586 | 59.64689924 | |
| isotretinoin PC3 UP | 4.00E-07 | 1.73E-04 | 26.95628415 | 397.090714 | |
| Tetradioxin | 1.65E-06 | 5.48E-04 | 1.97371904 | 26.27465804 | |
| Phytoestrogens | 2.09E-06 | 5.48E-04 | 13.84753588 | 181.1344061 | |
| progesterone | 2.21E-06 | 5.48E-04 | 2.276071635 | 29.63590994 | |
| AGN-PC-0JHFVD | 5.37E-06 | 0.001161625 | 5.512698934 | 66.89950185 | |
| folic acid | 6.29E-06 | 0.001211242 | 5.420999169 | 64.92143439 | |
| 1-NITROPYRENE | 8.34E-06 | 0.001334591 | 4.847108464 | 56.68435752 | |
