# Supplementary material for: Molecular biomarkers for the prognosis of breast cancer: role of amino acid metabolism genes
Source: J Physiol Biochem. 2025 Jun 10;81(2):441–57. doi: 10.1007/s13105-025-01088-5 (PMC12279611; doi:10.1007/s13105-025-01088-5)
Supplement: Supplementary file 2 — Supplementary Material 2 [file 13105_2025_1088_MOESM2_ESM.docx]

Figure 2

Data cleaning and standardization

#setwd('/pub1/data/mg_projects/projects/web_script/R/')

library(optparse)

option_list <- list(

make_option(c("-i", "--infile"), type = "character", default = '/pub1/data/mg_projects/projects/web_script/tool_runing/8bd5d9dd2ec97a191034afa8256e06d2/input.json',

action = "store", help = "Input a exp file path!"

),

make_option(c("-o", "--outfile"), type = "character", default = '/pub1/data/mg_projects/projects/web_script/web_file_catche/runing/dae42f7681eca28355c6ba9105a3b5ff',

action = "store", help = "Input a outfolder path!"

)

)

logs=c()

tryCatch({

Args <- commandArgs()

opt = parse_args(OptionParser(option_list = option_list, usage = "GEO Data press"))

logs=c(logs,paste0('run knn_norm.R-',basename(opt$outfile)))

#logs=c(logs,paste0('geting data:',paste0(paste0(names(opt),'=',opt),collapse = ',')))

#library("rjson")

library(jsonlite)

data<-jsonlite::stream_in(file(opt$infile),pagesize = 100)

exp_path=unlist(data$exp_path)

mutiRowName=unlist(data$mutiRowName)#'',mean,max,min,med

if(mutiRowName==''){

mutiRowName='med'

}

isKNN=as.numeric(unlist(data$isKNN))#0,1=KNN

cutRow_P=as.numeric(unlist(data$cutRow_P))#0-100

cutRow_Z=as.numeric(unlist(data$cutRow_Z))#0,1=NA

cutCol_P=as.numeric(unlist(data$cutCol_P))#0-100

cutCol_Z=as.numeric(unlist(data$cutCol_Z))#0,1=NA

normMethod=unlist(data$normMethod)#Q=quan,L=log2,X=log2X+1,ZC=col zscore,ZR=row zscore,Z=col and row zscore,N= none

#compare=rep(0,length(types))

dat=data.table::fread(exp_path, sep = "\t",header = T,stringsAsFactors = F,check.names = F

,na.strings="NA",data.table = F)

#head(dat)

rNames=dat[,1]

dat=dat[,-1]

logs=c(logs,paste0('read data row:',nrow(dat),',col:',ncol(dat)))

c_cnt=apply(dat, 2, function(x){

if(cutCol_Z==1) return (sum(is.na(x))/nrow(dat))

else return(sum(x==0)/nrow(dat))

})

c_cnt_sd=apply(dat, 2, function(x){

return(sd(x,na.rm = T))

})

c_inds=which(c_cnt<cutCol_P/100&c_cnt_sd>0)

logs=c(logs,paste0('Delete column:',(ncol(dat)-length(c_inds)),'/',ncol(dat)))

if(length(c_inds)>1){

dat=dat[,c_inds]

#head(dat)

r_cnt=apply(dat, 1, function(x){

if(cutRow_Z==1) return (sum(is.na(x))/ncol(dat))

else return(sum(x==0)/ncol(dat))

})

r_cnt_sd=apply(dat, 1, function(x){

return(sd(x,na.rm = T))

})

r_inds=which(r_cnt<cutRow_P/100&r_cnt_sd>0)

logs=c(logs,paste0('Delete row:',(nrow(dat)-length(r_inds)),'/',nrow(dat)))

if(length(r_inds)>1){

dat1=dat[r_inds,]

rNames1=rNames[r_inds]

row.names(dat1)=paste0('R',1:nrow(dat1))

if(isKNN>0){

logs=c(logs,paste0('start KNN K=',isKNN))

knn=impute::impute.knn(as.matrix(dat1) ,k = isKNN, rowmax = cutRow_P/100, colmax = cutCol_P/100,rng.seed=362436069)

ndat=knn$data

logs=c(logs,paste0('filter KNN column:',(ncol(dat1)-ncol(ndat)),',row:',(nrow(dat1)-nrow(ndat))))

ndat.rnames=rNames1[match(row.names(ndat),row.names(dat1))]

rNames1=ndat.rnames

dat1=ndat

}

if(normMethod=='Q'){#Q=quan,L=log2,X=log2X+1,ZC=col zscore,ZR=row zscore,Z=col and row zscore,N= none

#compare=rep(0,length(types))

dat1=limma::normalizeQuantiles(dat1)

logs=c(logs,paste0('normalize by normalizeQuantiles'))

}else if(normMethod=='M'){

dat1=limma::normalizeMedianAbsValues(dat1)

logs=c(logs,paste0('normalize by normalizeMedianAbsValues'))

}else if(normMethod=='X'){

dat1=log2(dat1+1)

logs=c(logs,paste0('normalize by log2(X+1)'))

}else if(normMethod=='L'){

dat1=log2(dat1)

logs=c(logs,paste0('normalize by log2'))

}else if(normMethod=='ZC'){

dat1=scale(dat1)

logs=c(logs,paste0('normalize by column z-score'))

}else if(normMethod=='ZR'){

dat1=t(scale(t(dat1)))

logs=c(logs,paste0('normalize by row z-score'))

}else if(normMethod=='Z'){

dat1=t(scale(t(scale(dat1))))

logs=c(logs,paste0('normalize by column z-score and row z-score'))

}

if(mutiRowName==''){

write.table(cbind(Tag=rNames1,dat1)

,file = paste0(opt$outfile,'/normalize.txt')

,row.names = F,col.names = T,quote = F,sep = '\t')

logs=c(logs,paste0('output row:',nrow(dat1),',col:',ncol(dat1)))

}else{

rns=table(rNames1)

muti=names(rns)[which(rns>1)]

sigO=names(rns)[which(rns==1)]

dat2=rbind()

dat2.name=unique(muti)

logs=c(logs,paste0('merge muti row name:',length(dat2.name),' by ',mutiRowName))

if(length(dat2.name)>0){

for(g in dat2.name){

dt=apply(dat1[which(rNames1%in%g),],2,function(x){#mean,max,min,med

if(mutiRowName=='mean') return(mean(x,na.rm = T))

else if(mutiRowName=='med') return(median(x,na.rm = T))

else if(mutiRowName=='max') return(max(x,na.rm = T))

else return(min(x,na.rm = T))

})

dat2=rbind(dat2,dt)

}

colnames(dat2)=colnames(dat1)

dat3=rbind(dat2,dat1[match(sigO,rNames1),])

}else{

dat3=dat1[match(sigO,rNames1),]

}

write.table(cbind(Tag=c(dat2.name,sigO),dat3)

,file = paste0(opt$outfile,'/normalize.txt')

,row.names = F,col.names = T,quote = F,sep = '\t')

logs=c(logs,paste0('output row:',nrow(dat3),',col:',ncol(dat3)))

}

}else{

logs=c(logs,paste0('Too few rows:<2!'))

}

}else{

logs=c(logs,paste0('Too few columns:<2!'))

}

},error = function(e) {

print(conditionMessage(e))

logs=c(logs,paste0('error:',conditionMessage(e)))

}, finally = {

write.table(logs,file = paste0(opt$outfile,'/run.log'),quote = F,row.names = T,col.names = T,sep = '\t')

})

**Consistency Clustering**

library(optparse)

option_list <- list(

make_option(c("-i", "--infile"), type = "character", default = '/pub1/data/mg_projects/projects/web_script/tool_runing/732700f6bf741bdd6ab5a9a518e5d002/input.json',

action = "store", help = "Input a exp file path!"

),

make_option(c("-o", "--outfile"), type = "character", default = '/pub1/data/mg_projects/projects/web_script/web_file_catche/runing/dae42f7681eca28355c6ba9105a3b5ff',

action = "store", help = "Input a outfolder path!"

)

)

logs=c()

tryCatch({

Args <- commandArgs()

opt = parse_args(OptionParser(option_list = option_list, usage = "Data press"))

#logs=c(logs,paste0('geting data:',paste0(paste0(names(opt),'=',opt),collapse = ',')))

logs=c(logs,paste0('run ConsensusClusterPlus.R-',basename(opt$outfile)))

library(jsonlite)

data<-jsonlite::stream_in(file(opt$infile),pagesize = 1000)

outFolder=opt$outfile

exp_path=data$exp_path

maxK=data$maxK

reps=data$reps#10

pItem=data$pItem#0.8

clusterAlg=data$clusterAlg#'hc'#pam,km,kmdist

innerLinkage='average'

finalLinkage='average'

distance=data$distance#'pearson'# 'spearman','euclidean','binary','maximum','canberra','minkowski"

logs=c(logs,'开始读取表达谱')

dat=data.table::fread(exp_path, sep = "\t",header = T,stringsAsFactors = F,check.names = F

,na.strings="NA",data.table = F,fill = T)

dat=dat[match(unique(dat[,1]),dat[,1]),]

row.names(dat)=dat[,1]

exp=dat[,-1]

logs=c(logs,paste0('表达谱行=',nrow(exp),',列=',ncol(exp)))

triangle=function (m, mode = 1)

{

n = dim(m)[1]

nm = matrix(0, ncol = n, nrow = n)

fm = m

nm[upper.tri(nm)] = m[upper.tri(m)]

fm = t(nm) + nm

diag(fm) = diag(m)

nm = fm

nm[upper.tri(nm)] = NA

diag(nm) = NA

vm = m[lower.tri(nm)]

if (mode == 1) {

return(vm)

}

else if (mode == 3) {

return(fm)

}

else if (mode == 2) {

return(nm)

}

}

run_cdf=function (ml, breaks = 100)

{

k = length(ml)

this_colors = rainbow(k - 1)

areaK = c()

all_line=rbind()

line_gap=c()

for (i in 1:length(ml)) {

v = triangle(ml[[i]], mode = 1)

h = hist(v, plot = FALSE, breaks = seq(0, 1, by = 1/breaks))

h$counts = cumsum(h$counts)/sum(h$counts)

thisArea = 0

for (bi in 1:(length(h$breaks) - 1)) {

thisArea = thisArea + h$counts[bi] * (h$breaks[bi + 1] - h$breaks[bi])

bi = bi + 1

}

areaK = c(areaK, thisArea)

h_lower=quantile(h$counts,seq(0,1,0.01))['5%']

h_upper=quantile(h$counts,seq(0,1,0.01))['95%']

line_gap=c(line_gap,h_upper-h_lower)

all_line=rbind(all_line,cbind(K=i+1,X=h$mids,Y=h$counts))

}

deltaK = areaK[1]

for (i in 2:(length(areaK))) {

deltaK = c(deltaK, (areaK[i] - areaK[i - 1])/areaK[i - 1])

}

return(list(CDF=all_line,Delta=cbind(K=1 + (1:length(deltaK)),Y=deltaK,lineGap=line_gap)))

}

getTreeNode=function(hc_obj){

dend=as.dendrogram(hc_obj)

capture.output(str(dend))->stx2

gsub('^ \\s+','',stx2)->stx2

nodeMap=rbind()

leafs=rbind()

all_node_obj=rbind()

for(i in length(stx2):1){

if(length(grep('--leaf',stx2[i]))==0){

h=unlist(stringr::str_split(stx2[i],'members at h = '))[2]

h=gsub(']','',h)

lefNode=unlist(stringr::str_split(stx2[i],' members at h = '))[1]

lefNode=unlist(stringr::str_split(lefNode,'branches and '))[2]

sub1=paste0('C',i+1)

all_node_obj[which(all_node_obj[,1]==paste0('C',i+1)),4]=paste0('C',i)

for(j in 1:nrow(all_node_obj)){

sub_mx=all_node_obj[which(all_node_obj[,1]==paste0('C',i+j+1)),]

if(sub_mx[4]==''){

sub2=sub_mx[1]

break()

}

}

all_node_obj[which(all_node_obj[,1]==sub2),4]=paste0('C',i)

all_node_obj=rbind(all_node_obj,c(paste0('C',i),sub1,sub2,'',lefNode))

nodeMap=rbind(nodeMap,c('',paste0('C',i),paste0('C',i+1),sub2,h))

}else{

node=gsub(' ','',unlist(stringr::str_split(stx2[i],'--leaf'))[2])

node=gsub('^ \\s+','',node)

node=gsub('\\s+ $','',node)

node=gsub('^"','',node)

node=gsub('"$','',node)

leafs=rbind(leafs,c(node,paste0('C',i)))

all_node_obj=rbind(all_node_obj,c(paste0('C',i),'','','',1))

nodeMap=rbind(nodeMap,c(node,paste0('C',i),'','',0))

}

}

od=rep(nrow(nodeMap),nrow(nodeMap))

od[match(leafs[,1],nodeMap[,1])]=nrow(leafs):1

nodeMap=nodeMap[order(od),]

return(nodeMap)

}

library(ConsensusClusterPlus)

Kvec = 2:maxK

logs=c(logs,'开始对行进行中心化处理')

#print(head(exp))

exp=as.matrix(exp)

d = sweep(exp,1, apply(exp,1,median,na.rm=T))

colnames(d)=paste0('C',1:ncol(d))

logs=c(logs,'开始一致性聚类')

rcc = ConsensusClusterPlus(d,maxK=maxK,reps=reps,pItem=pItem,pFeature=1,title="t",distance=distance,clusterAlg=clusterAlg

,innerLinkage=innerLinkage,seed = 123456)

logs=c(logs,'聚类完成，开始计算样本聚类一致性')

resICL = calcICL(rcc,title="t",plot = NULL)

logs=c(logs,'保存一致性聚类结果')

save(exp,d,rcc,resICL,file = paste0(outFolder,'/AllCluster.RData'))

#cmt=rcc[[2]]$consensusMatrix

zip_consensusMatrix=function(cmt){

m_cmt=data.table::melt(cmt)

cmt_dt=c()

for(i in 1:(ncol(cmt)-1)){

t_m_cmt=m_cmt[m_cmt[,2]==i,]

cmt_dt=c(cmt_dt,t_m_cmt[match((i+1):ncol(cmt),t_m_cmt[,1]),3])

}

#which(cmt_dt-cmt_dt2!=0)

str_mt=rep('',length(cmt_dt))

str_mt[which(cmt_dt==0)]='-'

str_mt[which(cmt_dt==1)]='+'

str_mt[which(cmt_dt!=0&cmt_dt!=1)]=paste0('.',floor(cmt_dt[which(cmt_dt!=0&cmt_dt!=1)]*1000))

return(paste0(str_mt,collapse = ''))

}

#length(cmt_dt)

#dim(rcc[[i]]$consensusMatrix)

all_ml=list()

clusterConsensus=c()

all_cluster_tree=rbind()

all_consensusMatrix=rbind()

all_consensusMatrix_zip=rbind()

for(i in Kvec){

#all_class=rbind(all_class,rcc[[i]]$consensusClass)

all_ml=c(all_ml,list(rcc[[i]]$ml))

all_consensusMatrix=rbind(all_consensusMatrix,cbind(K=i,rcc[[i]]$consensusMatrix))

#i=2

all_consensusMatrix_zip=rbind(all_consensusMatrix_zip,c(i,zip_consensusMatrix(rcc[[i]]$consensusMatrix)))

hc=rcc[[i]]$consensusTree

treeNode=getTreeNode(hc)

all_cluster_tree=rbind(all_cluster_tree,cbind(K=i,treeNode))

clusterConsensus=c(clusterConsensus,mean(resICL$clusterConsensus[resICL$clusterConsensus[,1]==i,3]))

}

#dim(rcc[[i]]$consensusMatrix)

#length(all_ml)

cdf_value=run_cdf(all_ml)

delta_vl0=cbind(1,cdf_value$CDF)

delta_vl=cbind(cdf_value$Delta,clusterConsensus)

colnames(delta_vl0)=paste0('C',1:4)

colnames(delta_vl)=paste0('C',1:4)

delta_v=rbind(delta_vl0,delta_vl)

clusterConsensus=cbind(0,resICL$clusterConsensus)

colnames(clusterConsensus)=paste0('C',1:4)

delta_v=rbind(delta_v,clusterConsensus)

#head(delta_v)

itemConsensus=resICL$itemConsensus[which(resICL$itemConsensus[,4]!=0),]

itemConsensus[,3]=match(itemConsensus[,3],colnames(d))

#exp=d

itemConsensus=cbind(0,'',itemConsensus)

colnames(itemConsensus)=paste0('C',1:6)

colnames(all_cluster_tree)=paste0('C',1:6)

all_cluster_tree_out=rbind(all_cluster_tree,as.matrix(itemConsensus))

#head(all_cluster_tree)

#all_cluster_tree[nrow(all_cluster_tree):(nrow(all_cluster_tree)-10),]

#length(rcc)

#rcc[[1]][,1:10]

cor_clust=unique(as.character(t(rcc[[1]])))

#setdiff(cor_clust,rcc[[1]][6,])

consensusClusterAll=apply(rcc[[1]], 2,function(x){

return(match(x,cor_clust))

})

tmp1=rbind(colnames(exp),as.matrix(consensusClusterAll))

#dim(tmp1)

#length(cor_clust)

#unique(rcc[[1]][9,])

#head(tmp1[,1:10])

consensusClusterAll_out=cbind(cor_clust,tmp1)

#consensusClusterAll_out[,1:10]

#head(all_cluster_tree_out)

#head(delta_v)

#head(all_consensusMatrix)

#head(consensusClusterAll_out)

logs=c(logs,'输出聚类结果')

write.table(all_cluster_tree_out,file = paste0(outFolder,'/cluster_tree.txt'),quote = F,row.names = F,col.names = F,sep = '\t')

write.table(delta_v,file = paste0(outFolder,'/cdf_delta.txt'),quote = F,row.names = F,col.names = F,sep = '\t')

#dim(all_consensusMatrix)

#511*9

#all_consensusMatrix[1,]

logs=c(logs,'zipConsensusMatrix')

#all_consensusMatrix_zip[1,]

#dim(all_consensusMatrix_zip)

#apply(array, margin, ...)

write.table(all_consensusMatrix,file = paste0(outFolder,'/consensusMatrix.txt'),quote = F,row.names = F,col.names = F,sep = '\t')

write.table(all_consensusMatrix_zip,file = paste0(outFolder,'/consensusMatrixZip.txt'),quote = F,row.names = F,col.names = F,sep = '\t')

write.table(consensusClusterAll_out,file = paste0(outFolder,'/cluster_all.txt'),quote = F,row.names = F,col.names = F,sep = '\t')

logs=c(logs,'run succ')

},error = function(e) {

print(conditionMessage(e))

logs=c(logs,paste0('error:',conditionMessage(e)))

}, finally = {

write.table(logs,file = paste0(opt$outfile,'/run.log'),quote = F,row.names = T,col.names = T,sep = '\t')

})

Heatmap

install.packages("ggplot2")

install.packages("reshape2")

library(ggplot2)

library(reshape2)

data_matrix <- as.matrix(data)

data_melted <- melt(data_matrix)

ggplot(data_melted, aes(x=Var2, y=Var1, fill=value)) +

geom_tile() +

scale_fill_gradient(low="blue", high="red") +

labs(x="Samples", y="Genes", fill="Expression Level") +

theme_minimal() +

theme(axis.text.x = element_text(angle=45, hjust=1))

PCA analysis

library(optparse)

option_list <- list(

make_option(c("-i", "--infile"), type = "character", default = '/pub1/data/mg_projects/projects/web_script/tool_runing/a7f2e171cab47364d8d8ab6c7fc99169/input.json',

action = "store", help = "Input a exp file path!"

),

make_option(c("-o", "--outfile"), type = "character", default = '/pub1/data/mg_projects/projects/web_script/web_file_catche/runing/dae42f7681eca28355c6ba9105a3b5ff',

action = "store", help = "Input a outfolder path!"

)

)

logs=c()

tryCatch({

Args <- commandArgs()

opt = parse_args(OptionParser(option_list = option_list, usage = "Data press"))

#logs=c(logs,paste0('geting data:',paste0(paste0(names(opt),'=',opt),collapse = ',')))

logs=c(logs,paste0('run pcg.R-',basename(opt$outfile)))

library(jsonlite)

data<-jsonlite::stream_in(file(opt$infile),pagesize = 1000)

method=unlist(data$method)

exp_path=unlist(data$exp_path)

groups=unlist(data$groups)

samples=unlist(data$samples)

outfolder=opt$outfile

logs=c(logs,paste0('reading data:',basename(exp_path)))

#exp_path='/pub1/data/mg_projects/projects/web_script/tool_runing/test_data/Merge_RNAseqCount.txt'

dat=data.table::fread(exp_path, sep = "\t",header = T,stringsAsFactors = F,check.names = F

,na.strings="NA",data.table = F)

dat=dat[match(unique(dat[,1]),dat[,1]),]

row.names(dat)=as.character(dat[,1])

dat=dat[,-1]

#which((dat[,1]==''))

logs=c(logs,paste0('readed data,ncol=',ncol(dat),'nrow=',nrow(dat)))

exp=apply(dat, 2, as.numeric)

row.names(exp)=row.names(dat)

logs=c(logs,'converted numeric')

dat=(exp[apply(t(exp), 2, function(x){

return(sd(x,na.rm=T))

})>0,])

nnames=row.names(dat)

if(sum(is.na(dat))>0){

dat=t(impute::impute.knn(as.matrix(t(dat)))$data)

}

colnames(dat)=colnames(exp)

row.names(dat)=nnames

if(method=='MVT'){

lmfit=limma::lmFit(dat)

logs=c(logs,'outping')

write.table(cbind(lmfit$Amean,sqrt(lmfit$sigma)),file = paste0(outfolder,'/stat.mtx'),quote = F,row.names = F,col.names = F,sep = '\t')

logs=c(logs,'outputed')

}else if(method=='boxplot'){

bx=apply(dat, 2, function(x){

q1=quantile(x, c(0,0.25, 0.5, 0.75,1), na.rm=T)

otl=1.5*(q1[4]-q1[2])

otl.u=ifelse(q1[4]+otl>q1[5],q1[5],q1[4]+otl)

otl.d=ifelse(q1[2]-otl<q1[1],q1[1],q1[2]-otl)

return(c(otl.d,q1[2:4],otl.u,sd(x,na.rm = T),mean(x,na.rm = T)))

})

logs=c(logs,'outping')

write.table(cbind(Tag=colnames(bx),t(bx)),file = paste0(outfolder,'/stat.mtx'),quote = F,row.names = F,col.names = F,sep = '\t')

logs=c(logs,'outputed')

}else if(method=='PCAplot'){

nn=ceiling(ncol(dat)/3)

if(nn>15) nn=15

if(nn<2) nn=2

library(umap)

p_input=t(dat)

p_input=as.data.frame(scale(p_input))

#colnames(p_input)=row.names(dat)

#colnames(p_input)

#class(p_input)

#head(p_input[,1:10])

logs=c(logs,paste0('runing umap,N=',nn))

iris.umap = umap(scale(p_input), n_neighbors = nn, random_state = 123)

umaply=iris.umap$layout

logs=c(logs,'outping umap')

write.table(cbind(Tag=row.names(umaply),umaply),file = paste0(outfolder,'/stat_UMAP.mtx'),quote = F,row.names = F,col.names = F,sep = '\t')

logs=c(logs,'outputed umap')

logs=c(logs,paste0('runing PCA',''))

pca1=stats::prcomp(p_input)

logs=c(logs,'pca runed')

as.matrix(p_input)

head(p_input)

unscale=function (data, center = NULL, scale = NULL)

{

if (is.null(scale)) {

scale <- attr(data, "scaled:scale")

}

if (is.null(center)) {

center <- attr(data, "scaled:center")

}

if (!is.null(scale) && !is.logical(scale)) {

data <- base::scale(data, center = FALSE, scale = 1/scale)

}

if (!is.null(center) && !is.logical(center)) {

data <- base::scale(data, center = -center, scale = FALSE)

}

as.data.frame(data)

}

fortify=function (model)

{

if (is(model, "prcomp")) {

d <- as.data.frame(model$x)

values <- model$x %*% t(model$rotation)

}

else if (is(model, "princomp")) {

d <- as.data.frame(model$scores)

values <- model$scores %*% t(model$loadings[, ])

}

else {

stop(paste0("Unsupported class for fortify.pca_common: ",

class(model)))

}

values <- unscale(values, center = model$center,

scale = model$scale)

#values <- cbind_wraps(data, values)

#cbind(data,values[match(row.names(data),row.names(values)),])

return(d);

#d <- cbind_wraps(values, d)

#post_fortify(d)

}

plot.data <- fortify(pca1)

#dim(plot.data)

#dim(plot.data2)

logs=c(logs,'fortify PCA')

plot.data$rownames <- rownames(plot.data)

ve <- pca1$sdev^2/sum(pca1$sdev^2)

PC <- paste0("PC", 1:length(pca1$sdev))

lam <- pca1$sdev

lam <- lam * sqrt(nrow(plot.data))

plot.data.pc <- t(t(plot.data[,PC])/lam)

otd=rbind(summary(pca1)[[6]][2,],plot.data.pc)

logs=c(logs,'outping PCA')

write.table(cbind(Tag=row.names(otd),otd)

,file = paste0(outfolder,'/stat_PCA.mtx')

,quote = F,row.names = F,col.names = F,sep = '\t')

logs=c(logs,'outputed PCA')

logs=c(logs,paste0('runing tSNE',''))

floor((nrow(p_input)-1 )/3)->tpk

if(tpk>0){

if(tpk>50) tpk=50

tsne <- Rtsne::Rtsne(as.matrix(p_input), check_duplicates = FALSE, pca = T,

perplexity=tpk, theta=0.0, dims=2)

logs=c(logs,'outping tSNE')

write.table(cbind(Tag=row.names(p_input), tsne$Y)

,file = paste0(outfolder,'/stat_tSNE.mtx')

,quote = F,row.names = F,col.names = F,sep = '\t')

logs=c(logs,'outputed tSNE')

}

}

},error = function(e) {

print(conditionMessage(e))

logs=c(logs,paste0('error:',conditionMessage(e)))

}, finally = {

write.table(logs,file = paste0(opt$outfile,'/run.log'),quote = F,row.names = T,col.names = T,sep = '\t')

})

Figure 3

ESTIMATE score

#setwd('/pub1/data/mg_projects/projects/web_script/R/')

library(optparse)

option_list <- list(

make_option(c("-i", "--infile"), type = "character", default = '/pub1/data/mg_projects/projects/web_script/tool_runing/37082fba10fc297522cb3156cfe1113f/input.json',

action = "store", help = "Input a exp file path!"

),

make_option(c("-o", "--outfile"), type = "character", default = '/pub1/data/mg_projects/projects/web_script/web_file_catche/runing/37082fba10fc297522cb3156cfe1113f',

action = "store", help = "Input a outfolder path!"

)

)

logs=c()

tryCatch({

Args <- commandArgs()

opt = parse_args(OptionParser(option_list = option_list, usage = "GEO Data press"))

logs=c(logs,paste0('run immu_cal.R-',basename(opt$outfile)))

#library("rjson")

library(jsonlite)

data<-jsonlite::stream_in(file(opt$infile),pagesize = 100)

exp_path=unlist(data$exp_path)

cancerCode=unlist(data$cancerCode)

array=as.numeric(unlist(data$array))#0,1=array

method=unlist(data$method)

#method='timer'

#array=1

#cancerCode='STAD'

dat=data.table::fread(exp_path, sep = "\t",header = T,stringsAsFactors = F,check.names = F

,na.strings="NA",data.table = F)

row.names(dat)=dat[,1]

dat=dat[,-1]

library(hgu133plus2.db)

if(sum(row.names(dat)%in%mappedRkeys(hgu133plus2SYMBOL))<2000){

logs=c(logs,paste0('error:Too few genes'))

}else{

library(IOBR)

deconvolute_timer.default<-function (args)

{

cancers = check_cancer_types(args)

TimerINFO("Loading immune gene expression")

immune <- immuneCuratedData

immune.geneExpression <- immune$genes

immune.cellTypes <- immune$celltypes

outlier.genes <- sort(GetOutlierGenes(cancers))

print(paste("Outlier genes:", paste(outlier.genes, collapse = " ")))

dir.create(args$outdir, showWarnings = FALSE, recursive = TRUE)

if (!dir.exists(paste(args$outdir, "/results", sep = ""))) {

dir.create(paste(args$outdir, "/results", sep = ""))

}

abundance.score.matrix <- c()

#pdf(paste(args$outdir, "/results/output.pdf", sep = ""))

for (i in 1:nrow(cancers)) {

cancer.expFile <- cancers[i, 1]

cancer.category <- cancers[i, 2]

cancer.expression <- ParseInputExpression(cancer.expFile)

index <- !(row.names(cancer.expression) %in% outlier.genes)

cancer.expression <- cancer.expression[index, , drop = FALSE]

cancer.colnames <- colnames(cancer.expression)

TimerINFO(paste("Removing the batch effect of", cancer.expFile))

#for (j in 1:length(cancer.colnames)) {

# DrawQQPlot(cancer.expression[, j], immune.geneExpression[,

# 1], name = cancer.colnames[j])

#}

tmp <- RemoveBatchEffect(cancer.expression, immune.geneExpression,

immune.cellTypes)

cancer.expNorm <- tmp[[1]]

immune.expNormMedian <- tmp[[3]]

#for (j in 1:length(cancer.colnames)) {

# DrawQQPlot(cancer.expNorm[, j], immune.expNormMedian[,

# 1], name = paste("After batch removing and aggregating for",

# cancer.colnames[j]))

#}

gene.selected.marker <- cancer_type_genes[[which(names(cancer_type_genes) ==

cancer.category)]]

gene.selected.marker <- intersect(gene.selected.marker,

row.names(cancer.expNorm))

XX = immune.expNormMedian[gene.selected.marker, c(-4)]

YY = cancer.expNorm[gene.selected.marker, , drop = FALSE]

for (j in 1:length(cancer.colnames)) {

fractions <- GetFractions.Abbas(XX, YY[, j])

#barplot(fractions, cex.names = 0.8, names.arg = names(fractions),

# xlab = "cell type", ylab = "abundance", main = paste("Abundance estimation for",

# cancer.colnames[j]))

#box()

abundance.score.matrix <- cbind(abundance.score.matrix,

fractions)

colnames(abundance.score.matrix)[ncol(abundance.score.matrix)] <- cancer.colnames[j]

}

}

#dev.off()

write.table(abundance.score.matrix, paste(args$outdir, "/results/score_matrix.txt",

sep = ""), sep = "\t", quote = FALSE, row.names = TRUE,

col.names = NA)

return(abundance.score.matrix)

}

deconvo_timer<-function (eset, project = NULL, indications = NULL)

{

indications = tolower(indications)

checkmate::assert("indications fit to mixture matrix", length(indications) ==

ncol(eset))

args = new.env()

args$outdir = tempdir()

args$batch = tempfile()

lapply(unique(indications), function(ind) {

tmp_file = tempfile()

tmp_mat = eset[, indications == ind, drop = FALSE] %>%

as_tibble(rownames = "gene_symbol")

readr::write_tsv(tmp_mat, tmp_file)

cat(paste0(tmp_file, ",", ind, "\n"), file = args$batch, append = TRUE)

})

results <- deconvolute_timer.default(args)[, make.names(colnames(eset))]

colnames(results) <- colnames(eset)

results <- as.data.frame(t(results))

colnames(results) <- paste(colnames(results), "_TIMER", sep = "")

colnames(results) <- gsub(colnames(results), pattern = "\\.", replacement = "\\_")

colnames(results) <- gsub(colnames(results), pattern = "\\ ", replacement = "\\_")

if (!is.null(project)) {

results$project <- project

results <- results[, c(ncol(results), 1:ncol(results) - 1)]

}

results <- tibble::rownames_to_column(results, var = "ID")

return(results)

}

array=(array==1)

tp=cancerCode

tp=tolower(tp)

fst_exp=as.matrix(dat)

logs=c(logs,paste0('start run immu,method=',method))

if(method=='timer'|method=='T'){

logs=c(logs,paste0('run timer'))

imu<-deconvo_timer(eset=fst_exp,indications = rep(tp,ncol(fst_exp)))

logs=c(logs,paste0('run end'))

colnames(imu)=gsub(paste0('_TIMER$'),'',colnames(imu))

}else if(method=='quantiseq'|method=='Q'){

logs=c(logs,paste0('run quantiseq'))

imu<-deconvo_quantiseq(fst_exp,tumor=TRUE,arrays = array,scale_mrna=T)

logs=c(logs,paste0('run end'))

colnames(imu)=gsub(paste0('_quantiseq$'),'',colnames(imu))

}else if(method=='mcpcounter'|method=='M'){

logs=c(logs,paste0('run mcpcounter'))

imu<-deconvo_mcpcounter(fst_exp)

logs=c(logs,paste0('run end'))

colnames(imu)=gsub(paste0('_MCPcounter$'),'',colnames(imu))

}else if(method=='estimate'|method=='ES'){

library(estimate)

logs=c(logs,paste0('run estimate'))

ptf='illumina'

if(array) ptf='affymetrix'

imu<-deconvo_estimate(fst_exp,platform=ptf)

logs=c(logs,paste0('run end'))

colnames(imu)=gsub(paste0('_estimate$'),'',colnames(imu))

}else if(method=='ips'|method=='I'){

logs=c(logs,paste0('run ips'))

imu<-deconvo_ips(fst_exp,plot = F)

logs=c(logs,paste0('run end'))

colnames(imu)=gsub(paste0('_IPS$'),'',colnames(imu))

}else if(method=='epic'|method=='EP'){

logs=c(logs,paste0('run epic'))

imu<-deconvo_epic(eset = fst_exp,tumor = TRUE)

logs=c(logs,paste0('run end'))

colnames(imu)=gsub(paste0('_EPIC$'),'',colnames(imu))

}else if(method=='xcell'|method=='X'){

logs=c(logs,paste0('run xcell'))

imu<-deconvo_xcell(fst_exp,arrays = array)

logs=c(logs,paste0('run end'))

colnames(imu)=gsub(paste0('_xCell$'),'',colnames(imu))

}else if(method=='cibersort'|method=='C'){

logs=c(logs,paste0('run cibersort'))

fst_exp1=fst_exp[,which(apply(fst_exp[row.names(fst_exp)%in%row.names(lm22),], 2,sd)>0)]

cibersort_result<-deconvo_cibersort(eset = fst_exp1,arrays = FALSE,absolute = FALSE, perm = 1000)

logs=c(logs,paste0('run end'))

narn=setdiff(colnames(fst_exp),colnames(fst_exp1))

if(length(narn)>0){

nam<-cbind(narn,matrix(rep(NA,25*length(narn)),ncol = 25))

colnames(nam)=colnames(cibersort_result)

cibersort_result=rbind(cibersort_result,nam)

}

imu=cibersort_result

}

logs=c(logs,paste0('outputing immu result'))

colnames(imu)=gsub(paste0('_',toupper(method),'$'),'',colnames(imu))

write.table(imu,file = paste0(opt$outfile,'/immueScore.txt')

,row.names = F,col.names = T,quote = F,sep = '\t')

logs=c(logs,paste0('outputed'))

}

},error = function(e) {

print(conditionMessage(e))

logs=c(logs,paste0('error:',conditionMessage(e)))

}, finally = {

write.table(logs,file = paste0(opt$outfile,'/run.log'),quote = F,row.names = T,col.names = T,sep = '\t')

})

MCP counter

#setwd('/pub1/data/mg_projects/projects/web_script/R/')

library(optparse)

option_list <- list(

make_option(c("-i", "--infile"), type = "character", default = '/pub1/data/mg_projects/projects/web_script/tool_runing/37082fba10fc297522cb3156cfe1113f/input.json',

action = "store", help = "Input a exp file path!"

),

make_option(c("-o", "--outfile"), type = "character", default = '/pub1/data/mg_projects/projects/web_script/web_file_catche/runing/37082fba10fc297522cb3156cfe1113f',

action = "store", help = "Input a outfolder path!"

)

)

logs=c()

tryCatch({

Args <- commandArgs()

opt = parse_args(OptionParser(option_list = option_list, usage = "GEO Data press"))

logs=c(logs,paste0('run immu_cal.R-',basename(opt$outfile)))

#library("rjson")

library(jsonlite)

data<-jsonlite::stream_in(file(opt$infile),pagesize = 100)

exp_path=unlist(data$exp_path)

cancerCode=unlist(data$cancerCode)

array=as.numeric(unlist(data$array))#0,1=array

method=unlist(data$method)

#method='timer'

#array=1

#cancerCode='STAD'

dat=data.table::fread(exp_path, sep = "\t",header = T,stringsAsFactors = F,check.names = F

,na.strings="NA",data.table = F)

row.names(dat)=dat[,1]

dat=dat[,-1]

library(hgu133plus2.db)

if(sum(row.names(dat)%in%mappedRkeys(hgu133plus2SYMBOL))<2000){

logs=c(logs,paste0('error:Too few genes'))

}else{

library(IOBR)

deconvolute_timer.default<-function (args)

{

cancers = check_cancer_types(args)

TimerINFO("Loading immune gene expression")

immune <- immuneCuratedData

immune.geneExpression <- immune$genes

immune.cellTypes <- immune$celltypes

outlier.genes <- sort(GetOutlierGenes(cancers))

print(paste("Outlier genes:", paste(outlier.genes, collapse = " ")))

dir.create(args$outdir, showWarnings = FALSE, recursive = TRUE)

if (!dir.exists(paste(args$outdir, "/results", sep = ""))) {

dir.create(paste(args$outdir, "/results", sep = ""))

}

abundance.score.matrix <- c()

#pdf(paste(args$outdir, "/results/output.pdf", sep = ""))

for (i in 1:nrow(cancers)) {

cancer.expFile <- cancers[i, 1]

cancer.category <- cancers[i, 2]

cancer.expression <- ParseInputExpression(cancer.expFile)

index <- !(row.names(cancer.expression) %in% outlier.genes)

cancer.expression <- cancer.expression[index, , drop = FALSE]

cancer.colnames <- colnames(cancer.expression)

TimerINFO(paste("Removing the batch effect of", cancer.expFile))

#for (j in 1:length(cancer.colnames)) {

# DrawQQPlot(cancer.expression[, j], immune.geneExpression[,

# 1], name = cancer.colnames[j])

#}

tmp <- RemoveBatchEffect(cancer.expression, immune.geneExpression,

immune.cellTypes)

cancer.expNorm <- tmp[[1]]

immune.expNormMedian <- tmp[[3]]

#for (j in 1:length(cancer.colnames)) {

# DrawQQPlot(cancer.expNorm[, j], immune.expNormMedian[,

# 1], name = paste("After batch removing and aggregating for",

# cancer.colnames[j]))

#}

gene.selected.marker <- cancer_type_genes[[which(names(cancer_type_genes) ==

cancer.category)]]

gene.selected.marker <- intersect(gene.selected.marker,

row.names(cancer.expNorm))

XX = immune.expNormMedian[gene.selected.marker, c(-4)]

YY = cancer.expNorm[gene.selected.marker, , drop = FALSE]

for (j in 1:length(cancer.colnames)) {

fractions <- GetFractions.Abbas(XX, YY[, j])

#barplot(fractions, cex.names = 0.8, names.arg = names(fractions),

# xlab = "cell type", ylab = "abundance", main = paste("Abundance estimation for",

# cancer.colnames[j]))

#box()

abundance.score.matrix <- cbind(abundance.score.matrix,

fractions)

colnames(abundance.score.matrix)[ncol(abundance.score.matrix)] <- cancer.colnames[j]

}

}

#dev.off()

write.table(abundance.score.matrix, paste(args$outdir, "/results/score_matrix.txt",

sep = ""), sep = "\t", quote = FALSE, row.names = TRUE,

col.names = NA)

return(abundance.score.matrix)

}

deconvo_timer<-function (eset, project = NULL, indications = NULL)

{

indications = tolower(indications)

checkmate::assert("indications fit to mixture matrix", length(indications) ==

ncol(eset))

args = new.env()

args$outdir = tempdir()

args$batch = tempfile()

lapply(unique(indications), function(ind) {

tmp_file = tempfile()

tmp_mat = eset[, indications == ind, drop = FALSE] %>%

as_tibble(rownames = "gene_symbol")

readr::write_tsv(tmp_mat, tmp_file)

cat(paste0(tmp_file, ",", ind, "\n"), file = args$batch, append = TRUE)

})

results <- deconvolute_timer.default(args)[, make.names(colnames(eset))]

colnames(results) <- colnames(eset)

results <- as.data.frame(t(results))

colnames(results) <- paste(colnames(results), "_TIMER", sep = "")

colnames(results) <- gsub(colnames(results), pattern = "\\.", replacement = "\\_")

colnames(results) <- gsub(colnames(results), pattern = "\\ ", replacement = "\\_")

if (!is.null(project)) {

results$project <- project

results <- results[, c(ncol(results), 1:ncol(results) - 1)]

}

results <- tibble::rownames_to_column(results, var = "ID")

return(results)

}

array=(array==1)

tp=cancerCode

tp=tolower(tp)

fst_exp=as.matrix(dat)

logs=c(logs,paste0('start run immu,method=',method))

if(method=='timer'|method=='T'){

logs=c(logs,paste0('run timer'))

imu<-deconvo_timer(eset=fst_exp,indications = rep(tp,ncol(fst_exp)))

logs=c(logs,paste0('run end'))

colnames(imu)=gsub(paste0('_TIMER$'),'',colnames(imu))

}else if(method=='quantiseq'|method=='Q'){

logs=c(logs,paste0('run quantiseq'))

imu<-deconvo_quantiseq(fst_exp,tumor=TRUE,arrays = array,scale_mrna=T)

logs=c(logs,paste0('run end'))

colnames(imu)=gsub(paste0('_quantiseq$'),'',colnames(imu))

}else if(method=='mcpcounter'|method=='M'){

logs=c(logs,paste0('run mcpcounter'))

imu<-deconvo_mcpcounter(fst_exp)

logs=c(logs,paste0('run end'))

colnames(imu)=gsub(paste0('_MCPcounter$'),'',colnames(imu))

}else if(method=='estimate'|method=='ES'){

library(estimate)

logs=c(logs,paste0('run estimate'))

ptf='illumina'

if(array) ptf='affymetrix'

imu<-deconvo_estimate(fst_exp,platform=ptf)

logs=c(logs,paste0('run end'))

colnames(imu)=gsub(paste0('_estimate$'),'',colnames(imu))

}else if(method=='ips'|method=='I'){

logs=c(logs,paste0('run ips'))

imu<-deconvo_ips(fst_exp,plot = F)

logs=c(logs,paste0('run end'))

colnames(imu)=gsub(paste0('_IPS$'),'',colnames(imu))

}else if(method=='epic'|method=='EP'){

logs=c(logs,paste0('run epic'))

imu<-deconvo_epic(eset = fst_exp,tumor = TRUE)

logs=c(logs,paste0('run end'))

colnames(imu)=gsub(paste0('_EPIC$'),'',colnames(imu))

}else if(method=='xcell'|method=='X'){

logs=c(logs,paste0('run xcell'))

imu<-deconvo_xcell(fst_exp,arrays = array)

logs=c(logs,paste0('run end'))

colnames(imu)=gsub(paste0('_xCell$'),'',colnames(imu))

}else if(method=='cibersort'|method=='C'){

logs=c(logs,paste0('run cibersort'))

fst_exp1=fst_exp[,which(apply(fst_exp[row.names(fst_exp)%in%row.names(lm22),], 2,sd)>0)]

cibersort_result<-deconvo_cibersort(eset = fst_exp1,arrays = FALSE,absolute = FALSE, perm = 1000)

logs=c(logs,paste0('run end'))

narn=setdiff(colnames(fst_exp),colnames(fst_exp1))

if(length(narn)>0){

nam<-cbind(narn,matrix(rep(NA,25*length(narn)),ncol = 25))

colnames(nam)=colnames(cibersort_result)

cibersort_result=rbind(cibersort_result,nam)

}

imu=cibersort_result

}

logs=c(logs,paste0('outputing immu result'))

colnames(imu)=gsub(paste0('_',toupper(method),'$'),'',colnames(imu))

write.table(imu,file = paste0(opt$outfile,'/immueScore.txt')

,row.names = F,col.names = T,quote = F,sep = '\t')

logs=c(logs,paste0('outputed'))

}

},error = function(e) {

print(conditionMessage(e))

logs=c(logs,paste0('error:',conditionMessage(e)))

}, finally = {

write.table(logs,file = paste0(opt$outfile,'/run.log'),quote = F,row.names = T,col.names = T,sep = '\t')

})

CIBERSORT

#setwd('/pub1/data/mg_projects/projects/web_script/R/')

library(optparse)

option_list <- list(

make_option(c("-i", "--infile"), type = "character", default = '/pub1/data/mg_projects/projects/web_script/tool_runing/37082fba10fc297522cb3156cfe1113f/input.json',

action = "store", help = "Input a exp file path!"

),

make_option(c("-o", "--outfile"), type = "character", default = '/pub1/data/mg_projects/projects/web_script/web_file_catche/runing/37082fba10fc297522cb3156cfe1113f',

action = "store", help = "Input a outfolder path!"

)

)

logs=c()

tryCatch({

Args <- commandArgs()

opt = parse_args(OptionParser(option_list = option_list, usage = "GEO Data press"))

logs=c(logs,paste0('run immu_cal.R-',basename(opt$outfile)))

#library("rjson")

library(jsonlite)

data<-jsonlite::stream_in(file(opt$infile),pagesize = 100)

exp_path=unlist(data$exp_path)

cancerCode=unlist(data$cancerCode)

array=as.numeric(unlist(data$array))#0,1=array

method=unlist(data$method)

#method='timer'

#array=1

#cancerCode='STAD'

dat=data.table::fread(exp_path, sep = "\t",header = T,stringsAsFactors = F,check.names = F

,na.strings="NA",data.table = F)

row.names(dat)=dat[,1]

dat=dat[,-1]

library(hgu133plus2.db)

if(sum(row.names(dat)%in%mappedRkeys(hgu133plus2SYMBOL))<2000){

logs=c(logs,paste0('error:Too few genes'))

}else{

library(IOBR)

deconvolute_timer.default<-function (args)

{

cancers = check_cancer_types(args)

TimerINFO("Loading immune gene expression")

immune <- immuneCuratedData

immune.geneExpression <- immune$genes

immune.cellTypes <- immune$celltypes

outlier.genes <- sort(GetOutlierGenes(cancers))

print(paste("Outlier genes:", paste(outlier.genes, collapse = " ")))

dir.create(args$outdir, showWarnings = FALSE, recursive = TRUE)

if (!dir.exists(paste(args$outdir, "/results", sep = ""))) {

dir.create(paste(args$outdir, "/results", sep = ""))

}

abundance.score.matrix <- c()

#pdf(paste(args$outdir, "/results/output.pdf", sep = ""))

for (i in 1:nrow(cancers)) {

cancer.expFile <- cancers[i, 1]

cancer.category <- cancers[i, 2]

cancer.expression <- ParseInputExpression(cancer.expFile)

index <- !(row.names(cancer.expression) %in% outlier.genes)

cancer.expression <- cancer.expression[index, , drop = FALSE]

cancer.colnames <- colnames(cancer.expression)

TimerINFO(paste("Removing the batch effect of", cancer.expFile))

#for (j in 1:length(cancer.colnames)) {

# DrawQQPlot(cancer.expression[, j], immune.geneExpression[,

# 1], name = cancer.colnames[j])

#}

tmp <- RemoveBatchEffect(cancer.expression, immune.geneExpression,

immune.cellTypes)

cancer.expNorm <- tmp[[1]]

immune.expNormMedian <- tmp[[3]]

#for (j in 1:length(cancer.colnames)) {

# DrawQQPlot(cancer.expNorm[, j], immune.expNormMedian[,

# 1], name = paste("After batch removing and aggregating for",

# cancer.colnames[j]))

#}

gene.selected.marker <- cancer_type_genes[[which(names(cancer_type_genes) ==

cancer.category)]]

gene.selected.marker <- intersect(gene.selected.marker,

row.names(cancer.expNorm))

XX = immune.expNormMedian[gene.selected.marker, c(-4)]

YY = cancer.expNorm[gene.selected.marker, , drop = FALSE]

for (j in 1:length(cancer.colnames)) {

fractions <- GetFractions.Abbas(XX, YY[, j])

#barplot(fractions, cex.names = 0.8, names.arg = names(fractions),

# xlab = "cell type", ylab = "abundance", main = paste("Abundance estimation for",

# cancer.colnames[j]))

#box()

abundance.score.matrix <- cbind(abundance.score.matrix,

fractions)

colnames(abundance.score.matrix)[ncol(abundance.score.matrix)] <- cancer.colnames[j]

}

}

#dev.off()

write.table(abundance.score.matrix, paste(args$outdir, "/results/score_matrix.txt",

sep = ""), sep = "\t", quote = FALSE, row.names = TRUE,

col.names = NA)

return(abundance.score.matrix)

}

deconvo_timer<-function (eset, project = NULL, indications = NULL)

{

indications = tolower(indications)

checkmate::assert("indications fit to mixture matrix", length(indications) ==

ncol(eset))

args = new.env()

args$outdir = tempdir()

args$batch = tempfile()

lapply(unique(indications), function(ind) {

tmp_file = tempfile()

tmp_mat = eset[, indications == ind, drop = FALSE] %>%

as_tibble(rownames = "gene_symbol")

readr::write_tsv(tmp_mat, tmp_file)

cat(paste0(tmp_file, ",", ind, "\n"), file = args$batch, append = TRUE)

})

results <- deconvolute_timer.default(args)[, make.names(colnames(eset))]

colnames(results) <- colnames(eset)

results <- as.data.frame(t(results))

colnames(results) <- paste(colnames(results), "_TIMER", sep = "")

colnames(results) <- gsub(colnames(results), pattern = "\\.", replacement = "\\_")

colnames(results) <- gsub(colnames(results), pattern = "\\ ", replacement = "\\_")

if (!is.null(project)) {

results$project <- project

results <- results[, c(ncol(results), 1:ncol(results) - 1)]

}

results <- tibble::rownames_to_column(results, var = "ID")

return(results)

}

array=(array==1)

tp=cancerCode

tp=tolower(tp)

fst_exp=as.matrix(dat)

logs=c(logs,paste0('start run immu,method=',method))

if(method=='timer'|method=='T'){

logs=c(logs,paste0('run timer'))

imu<-deconvo_timer(eset=fst_exp,indications = rep(tp,ncol(fst_exp)))

logs=c(logs,paste0('run end'))

colnames(imu)=gsub(paste0('_TIMER$'),'',colnames(imu))

}else if(method=='quantiseq'|method=='Q'){

logs=c(logs,paste0('run quantiseq'))

imu<-deconvo_quantiseq(fst_exp,tumor=TRUE,arrays = array,scale_mrna=T)

logs=c(logs,paste0('run end'))

colnames(imu)=gsub(paste0('_quantiseq$'),'',colnames(imu))

}else if(method=='mcpcounter'|method=='M'){

logs=c(logs,paste0('run mcpcounter'))

imu<-deconvo_mcpcounter(fst_exp)

logs=c(logs,paste0('run end'))

colnames(imu)=gsub(paste0('_MCPcounter$'),'',colnames(imu))

}else if(method=='estimate'|method=='ES'){

library(estimate)

logs=c(logs,paste0('run estimate'))

ptf='illumina'

if(array) ptf='affymetrix'

imu<-deconvo_estimate(fst_exp,platform=ptf)

logs=c(logs,paste0('run end'))

colnames(imu)=gsub(paste0('_estimate$'),'',colnames(imu))

}else if(method=='ips'|method=='I'){

logs=c(logs,paste0('run ips'))

imu<-deconvo_ips(fst_exp,plot = F)

logs=c(logs,paste0('run end'))

colnames(imu)=gsub(paste0('_IPS$'),'',colnames(imu))

}else if(method=='epic'|method=='EP'){

logs=c(logs,paste0('run epic'))

imu<-deconvo_epic(eset = fst_exp,tumor = TRUE)

logs=c(logs,paste0('run end'))

colnames(imu)=gsub(paste0('_EPIC$'),'',colnames(imu))

}else if(method=='xcell'|method=='X'){

logs=c(logs,paste0('run xcell'))

imu<-deconvo_xcell(fst_exp,arrays = array)

logs=c(logs,paste0('run end'))

colnames(imu)=gsub(paste0('_xCell$'),'',colnames(imu))

}else if(method=='cibersort'|method=='C'){

logs=c(logs,paste0('run cibersort'))

fst_exp1=fst_exp[,which(apply(fst_exp[row.names(fst_exp)%in%row.names(lm22),], 2,sd)>0)]

cibersort_result<-deconvo_cibersort(eset = fst_exp1,arrays = FALSE,absolute = FALSE, perm = 1000)

logs=c(logs,paste0('run end'))

narn=setdiff(colnames(fst_exp),colnames(fst_exp1))

if(length(narn)>0){

nam<-cbind(narn,matrix(rep(NA,25*length(narn)),ncol = 25))

colnames(nam)=colnames(cibersort_result)

cibersort_result=rbind(cibersort_result,nam)

}

imu=cibersort_result

}

logs=c(logs,paste0('outputing immu result'))

colnames(imu)=gsub(paste0('_',toupper(method),'$'),'',colnames(imu))

write.table(imu,file = paste0(opt$outfile,'/immueScore.txt')

,row.names = F,col.names = T,quote = F,sep = '\t')

logs=c(logs,paste0('outputed'))

}

},error = function(e) {

print(conditionMessage(e))

logs=c(logs,paste0('error:',conditionMessage(e)))

}, finally = {

write.table(logs,file = paste0(opt$outfile,'/run.log'),quote = F,row.names = T,col.names = T,sep = '\t')

})

Figure 4

#setwd('/pub1/data/mg_projects/projects/web_script/R/')

library(optparse)

option_list <- list(

make_option(c("-i", "--infile"), type = "character", default = '/pub1/data/mg_projects/projects/web_file_catche/runing/tool_runing/c055430ed9b4f109b0ae12591bd48044/input.json',

action = "store", help = "Input a exp file path!"

),

make_option(c("-o", "--outfile"), type = "character", default = '/pub1/data/mg_projects/projects/web_script/web_file_catche/runing/dae42f7681eca28355c6ba9105a3b5ff',

action = "store", help = "Input a outfolder path!"

)

)

logs=c()

tryCatch({

Args <- commandArgs()

opt = parse_args(OptionParser(option_list = option_list, usage = "GEO Data press"))

logs=c(logs,paste0('run deg_base.R-',basename(opt$outfile)))

#library("rjson")

library(jsonlite)

data<-jsonlite::stream_in(file(opt$infile),pagesize = 100)

if(unlist(data$method)=='filter'){

outpath=unlist(data$outpath)

pvalue=as.numeric(as.character(unlist(data$pvalue)))

lfc=as.numeric(as.character(unlist(data$lfc)))

isFDR=as.numeric(as.character(unlist(data$isFDR)))

data1<-jsonlite::stream_in(file(paste0(outpath,'/input.json')),pagesize = 100)

compare=unlist(data1$compare)

groups=unlist(data1$groups)

types=unlist(data1$types)

method=data1$method

cmp=unique(compare)

file_dat=rbind()

for(u in cmp){

#u=cmp[1]

#paste0(opt$outfile,'/',u,'_exp.mtx')

deg_path=paste0(outpath,'/',u,'_deg.mtx')

inds=which(compare==u)

group=groups[inds]

tp=types[inds]

ulab=(group[which(tp==1)][1])

dlab=(group[which(tp==0)][1])

dat=data.table::fread(deg_path, sep = "\t",header = T,stringsAsFactors = F,check.names = F,na.strings="NA",data.table = F)

lfcs=as.numeric(as.character(dat[,2]))

if(isFDR==1){

if(method=='limma'|method=='limmaVoom'){

ps=as.numeric(as.character(dat[,6]))

}else if(method=='rankTest'|method=='TTest'|method=='deseq2'|method=='edgR'){

ps=as.numeric(as.character(dat[,4]))

}

}else{

if(method=='limma'|method=='limmaVoom'){

ps=as.numeric(as.character(dat[,5]))

}else if(method=='rankTest'|method=='TTest'|method=='deseq2'|method=='edgR'){

ps=as.numeric(as.character(dat[,3]))

}

}

t.inds=which(ps<pvalue&abs(lfcs)>lfc)

uCnt=length(which(ps<pvalue&lfcs>lfc))

dCnt=length(which(ps<pvalue&lfcs<lfc*-1))

write.table(dat[t.inds,],file = paste0(opt$outfile,'/',u,'_filter.mtx'),row.names = F,col.names = T,quote = F,sep = '\t')

file_dat=rbind(file_dat,c(paste0(ulab,'-vs-',dlab)

,paste0(opt$outfile,'/',u,'_filter.mtx'),uCnt,dCnt))

}

write.table(file_dat

,file = paste0(opt$outfile,'/stat.mtx'),row.names = F,col.names = F,quote = F,sep = '\t')

}else{

exp_path=unlist(data$exp_path)

samples=unlist(data$samples)

groups=unlist(data$groups)

types=unlist(data$types)

compare=unlist(data$compare)

#compare=rep(0,length(types))

dat=data.table::fread(exp_path, sep = "\t",header = T,stringsAsFactors = F,check.names = F,na.strings="NA",data.table = F)

#head(dat)

logs=c(logs,paste0('read data nrow=',nrow(dat),',ncol=',ncol(dat)))

unm=unique(dat[,1])

dat=dat[match(unm,dat[,1]),]

row.names(dat)=dat[,1]

dat=dat[,-1]

limmaDEG=function(exp,group,ulab,dlab){

library(limma)

ind1=which(group==ulab)

ind2=which(group==dlab)

sml <- c(rep('G1',length(ind1)),rep('G0',length(ind2)))

eset=exp[,c(ind1,ind2)]

fl <- as.factor(sml)

design <- model.matrix(~fl+0)

colnames(design) <- levels(fl)

cont.matrix<-makeContrasts(contrasts='G1-G0',levels=design)

fit<-lmFit (eset,design)

fit2 <- contrasts.fit(fit, cont.matrix)

fit2 <- eBayes(fit2)

tT <- topTable(fit2, adjust="fdr", sort.by="B", number=nrow(eset))

eset=eset[match(row.names(tT),row.names(eset)),]

regulated=ifelse(tT$logFC>0,'Up','Down')

lfcs=c(log2(1.2),log2(1.3),log2(1.5),1)

all.deg.cnt=cbind()

for(lfc in lfcs){

deg1=regulated[which(abs(tT$logFC)>lfc&tT$P.Value<0.05)]

deg2=regulated[which(abs(tT$logFC)>lfc&tT$P.Value<0.01)]

deg3=regulated[which(abs(tT$logFC)>lfc&tT$adj.P.Val<0.05)]

deg4=regulated[which(abs(tT$logFC)>lfc&tT$adj.P.Val<0.01)]

all.deg.cnt=cbind(all.deg.cnt,c(paste0(sum(deg1=='Up'),'|',sum(deg1=='Down'))

,paste0(sum(deg2=='Up'),'|',sum(deg2=='Down'))

,paste0(sum(deg3=='Up'),'|',sum(deg3=='Down'))

,paste0(sum(deg4=='Up'),'|',sum(deg4=='Down'))))

}

row.names(all.deg.cnt)=c('p<0.05','p<0.01','FDR<0.05','FDR<0.01')

colnames(all.deg.cnt)=paste0(c('1.2','1.3','1.5','2'),'-fold')

return(list(Exp=eset,Group=group[c(ind1,ind2)],DEG=tT,Summary=all.deg.cnt))

}

testDEG=function(exp,group,ulab,dlab,rank=1){

ind1=which(group==ulab)

ind2=which(group==dlab)

gmn=min(exp,na.rm = T)

if(gmn<0){

cv_exp=apply(exp, 2, function(x){

#print((x-min(x,na.rm = T))/(max(x,na.rm = T)-min(x,na.rm = T)))

return ((x-min(x,na.rm = T))/(max(x,na.rm = T)-min(x,na.rm = T)))

})

fc=apply(cv_exp, 1, function(x){

return(mean(x[ind1],na.rm = T)/mean(x[ind2],na.rm = T))

})

}else{

fc=apply(exp, 1, function(x){

return(mean(x[ind1],na.rm = T)/mean(x[ind2],na.rm = T))

})

}

pv=apply(exp, 1, function(x){

#x=as.numeric(exp[1,])

x[ind1]->x1

x[ind2]->x2

x1=x1[!is.na(x1)]

x2=x2[!is.na(x2)]

p=1

if(length(x1)>2&length(x2)>2){

if(rank==1){

wilcox.test(x1,x2)->tt

p=tt$p.value

}else{

t.test(x1,x2)->tt

p=tt$p.value

}

}

return(p)

})

fdr=p.adjust(pv)

fc=log2(fc)

eset=exp[,c(ind1,ind2)]

#sum(fc>0.1)

regulated=ifelse(fc>0,'Up','Down')

lfcs=c(log2(1.2),log2(1.3),log2(1.5),1)

all.deg.cnt=cbind()

for(lfc in lfcs){

deg1=regulated[which(abs(fc)>lfc&pv<0.05)]

deg2=regulated[which(abs(fc)>lfc&pv<0.01)]

deg3=regulated[which(abs(fc)>lfc&fdr<0.05)]

deg4=regulated[which(abs(fc)>lfc&fdr<0.01)]

all.deg.cnt=cbind(all.deg.cnt,c(paste0(sum(deg1=='Up'),'|',sum(deg1=='Down'))

,paste0(sum(deg2=='Up'),'|',sum(deg2=='Down'))

,paste0(sum(deg3=='Up'),'|',sum(deg3=='Down'))

,paste0(sum(deg4=='Up'),'|',sum(deg4=='Down'))))

}

row.names(all.deg.cnt)=c('p<0.05','p<0.01','FDR<0.05','FDR<0.01')

colnames(all.deg.cnt)=paste0(c('1.2','1.3','1.5','2'),'-fold')

tT=cbind(lfc=fc,pvalue=pv,FDR=fdr)

row.names(tT)=row.names(eset)

return(list(Exp=eset[order(pv),],Group=group[c(ind1,ind2)],DEG=tT[order(pv),],Summary=all.deg.cnt))

}

countDEG=function(lfcValues,pvalue,fdr){

lfcs=c(log2(1.2),log2(1.3),log2(1.5),1)

regulated=ifelse(lfcValues>0,'Up','Down')

all.deg.cnt=cbind()

for(lfc in lfcs){

deg1=regulated[which(abs(lfcValues)>lfc&pvalue<0.05)]

deg2=regulated[which(abs(lfcValues)>lfc&pvalue<0.01)]

deg3=regulated[which(abs(lfcValues)>lfc&fdr<0.05)]

deg4=regulated[which(abs(lfcValues)>lfc&fdr<0.01)]

all.deg.cnt=cbind(all.deg.cnt,c(paste0(sum(deg1=='Up'),'|',sum(deg1=='Down'))

,paste0(sum(deg2=='Up'),'|',sum(deg2=='Down'))

,paste0(sum(deg3=='Up'),'|',sum(deg3=='Down'))

,paste0(sum(deg4=='Up'),'|',sum(deg4=='Down'))))

}

row.names(all.deg.cnt)=c('p<0.05','p<0.01','FDR<0.05','FDR<0.01')

colnames(all.deg.cnt)=paste0(c('1.2','1.3','1.5','2'),'-fold')

return(all.deg.cnt)

}

edgRDEG=function(exp,group,ulab,dlab){

library(edgeR)

ind1=which(group==ulab)

ind2=which(group==dlab)

eset=exp[,c(ind1,ind2)]

group_list <- c(rep('G1',length(ind1)),rep('G0',length(ind2)))

y <- DGEList(counts=eset,group=group_list)

y <- calcNormFactors(y)

y <- estimateCommonDisp(y)

y <- estimateTagwiseDisp(y)

et <- exactTest(y)

tT=data.frame(logFC=et$table$logFC,pvalue=et$table$PValue,FDR=p.adjust(et$table$PValue),logCPM=et$table$logCPM)

row.names(tT)=row.names(et$table)

tT=tT[order(tT$FDR),]

eset <- cpm(y, log=F, prior.count=2)

eset=eset[match(row.names(tT),row.names(eset)),]

return(list(Exp=eset,Group=group[c(ind1,ind2)],DEG=tT,Summary=countDEG(tT$logFC,tT$pvalue,tT$FDR)))

}

DESeq2DEG=function(exp,group,ulab,dlab){

library(DESeq2)

ind1=which(group==ulab)

ind2=which(group==dlab)

eset=exp[,c(ind1,ind2)]

group_list <- c(rep('G1',length(ind1)),rep('G0',length(ind2)))

dds <- DESeqDataSetFromMatrix(countData = eset,

colData = data.frame(row.names=factor(colnames(eset)), group_list=group_list),

design = ~ group_list)

dds2 <- DESeq(dds)

resultsNames(dds2)

res <- results(dds2, contrast=c("group_list","G1","G0"))

resOrdered <- res[order(res$padj),]

tT=data.frame(logFC=res$log2FoldChange,pvalue=res$pvalue,FDR=res$padj)

row.names(tT)=row.names(res)

tT=tT[order(tT$FDR),]

eset=counts(dds2, normalized=TRUE)

eset=eset[match(row.names(tT),row.names(eset)),]

return(list(Exp=eset,Group=group[c(ind1,ind2)],DEG=tT,Summary=countDEG(tT$logFC,tT$pvalue,tT$FDR)))

}

limmaVoom=function(exp,group,ulab,dlab){

library(limma)

ind1=which(group==ulab)

ind2=which(group==dlab)

eset=exp[,c(ind1,ind2)]

sml <- c(rep('G1',length(ind1)),rep('G0',length(ind2)))

fl <- as.factor(sml)

design <- model.matrix(~fl+0)

colnames(design) <- levels(fl)

v <- voom(eset, design, plot=F, normalize="quantile")

fit <- lmFit(v, design)

cont.matrix<-makeContrasts(contrasts='G1-G0',levels=design)

fit2 <- contrasts.fit(fit, cont.matrix)

fit2 <- eBayes(fit2)

tT <- topTable(fit2, adjust="fdr", sort.by="B", number=nrow(eset))

tT=tT[order(tT$adj.P.Val),]

eset=v$E

eset=eset[match(row.names(tT),row.names(eset)),]

return(list(Exp=eset,Group=group[c(ind1,ind2)],DEG=tT,Summary=countDEG(tT$logFC,tT$P.Value,tT$adj.P.Val)))

}

logs=c(logs,paste0('run ',data$method,' nrow=',nrow(dat),',ncol=',ncol(dat)))

if(unlist(data$method)=='limma'){

if(data$log==1){

dat=log2(dat)

}

cmp=unique(compare)

all_stat=rbind()

for(u in cmp){

#u=cmp[1]

inds=which(compare==u)

group=groups[inds]

exp=dat[,match(samples[inds],colnames(dat))]

tp=types[inds]

ulab=(group[which(tp==1)][1])

dlab=(group[which(tp==0)][1])

deg=limmaDEG(exp,group,ulab,dlab)

all_stat=rbind(all_stat,

cbind(Group=paste0(ulab,'-vs-',dlab),deg$Summary))

write.table(cbind(Tag=row.names(deg$Exp),deg$Exp)

,file = paste0(opt$outfile,'/',u,'_exp.mtx'),row.names = F,col.names = T,quote = F,sep = '\t')

write.table(cbind(Tag=row.names(deg$DEG),deg$DEG)

,file = paste0(opt$outfile,'/',u,'_deg.mtx'),row.names = F,col.names = T,quote = F,sep = '\t')

logs=c(logs,paste0('output deg:',ulab,'-vs-',dlab))

}

write.table(cbind(Tag=row.names(all_stat),all_stat)

,file = paste0(opt$outfile,'/stat.mtx'),row.names = F,col.names = T,quote = F,sep = '\t')

logs=c(logs,paste0('succ'))

}else if(unlist(data$method)=='rankTest'|unlist(data$method)=='TTest'){

if(unlist(data$method)=='rankTest'){

rank=1

}else{

rank=0

}

if(data$log==1){

dat=log2(dat)

}

cmp=unique(compare)

all_stat=rbind()

for(u in cmp){

#u=cmp[1]

inds=which(compare==u)

group=groups[inds]

exp=dat[,match(samples[inds],colnames(dat))]

tp=types[inds]

ulab=(group[which(tp==1)][1])

dlab=(group[which(tp==0)][1])

deg=testDEG(exp,group,ulab,dlab,rank)

all_stat=rbind(all_stat,cbind(Group=paste0(ulab,'-vs-',dlab),deg$Summary))

write.table(cbind(Tag=row.names(deg$Exp),deg$Exp)

,file = paste0(opt$outfile,'/',u,'_exp.mtx'),row.names = F,col.names = T,quote = F,sep = '\t')

write.table(cbind(Tag=row.names(deg$DEG),deg$DEG)

,file = paste0(opt$outfile,'/',u,'_deg.mtx'),row.names = F,col.names = T,quote = F,sep = '\t')

logs=c(logs,paste0('output deg:',ulab,'-vs-',dlab))

}

write.table(cbind(Tag=row.names(all_stat),all_stat)

,file = paste0(opt$outfile,'/stat.mtx'),row.names = F,col.names = T,quote = F,sep = '\t')

logs=c(logs,paste0('succ'))

}else if(unlist(data$method)%in%c('limmaVoom','deseq2','edgR')){

ctr=as.numeric(unlist(data$cutRowN))/100

#head(dat)

dat=floor(dat)

ctr.ps=apply(dat, 1, function(x){

return(sum(x>0,na.rm = T))

})/ncol(dat)

t.inds=which(ctr.ps>ctr)

if(length(t.inds)>2){

dat=dat[t.inds,]

cmp=unique(compare)

all_stat=rbind()

for(u in cmp){

inds=which(compare==u)

group=groups[inds]

exp=dat[,match(samples[inds],colnames(dat))]

tp=types[inds]

ulab=(group[which(tp==1)][1])

dlab=(group[which(tp==0)][1])

deg=NULL

if(unlist(data$method)=='limmaVoom'){

deg=limmaVoom(exp,group,ulab,dlab)

}else if(unlist(data$method)=='deseq2'){

deg=DESeq2DEG(exp,group,ulab,dlab)

}else if(unlist(data$method)=='edgR'){

deg=edgRDEG(exp,group,ulab,dlab)

}

all_stat=rbind(all_stat,cbind(Group=paste0(ulab,'-vs-',dlab),deg$Summary))

write.table(cbind(Tag=row.names(deg$Exp),deg$Exp)

,file = paste0(opt$outfile,'/',u,'_exp.mtx'),row.names = F,col.names = T,quote = F,sep = '\t')

write.table(cbind(Tag=row.names(deg$DEG),deg$DEG)

,file = paste0(opt$outfile,'/',u,'_deg.mtx'),row.names = F,col.names = T,quote = F,sep = '\t')

logs=c(logs,paste0('output deg:',ulab,'-vs-',dlab))

}

write.table(cbind(Tag=row.names(all_stat),all_stat)

,file = paste0(opt$outfile,'/stat.mtx'),row.names = F,col.names = T,quote = F,sep = '\t')

logs=c(logs,paste0('succ'))

}else{

logs=c(logs,paste0('error:','filter count last<3'))

}

}

}

},error = function(e) {

print(conditionMessage(e))

logs=c(logs,paste0('error:',conditionMessage(e)))

}, finally = {

write.table(logs,file = paste0(opt$outfile,'/run.log'),quote = F,row.names = T,col.names = T,sep = '\t')

})

KEGG analysis

library(optparse)

option_list <- list(

make_option(c("-i", "--infile"), type = "character", default = '/pub1/data/mg_projects/projects/web_script/tool_runing/48d62680c879ba86522cfdfa2c641491/input.json',

action = "store", help = "Input a exp file path!"

),

make_option(c("-o", "--outfile"), type = "character", default = '/pub1/data/mg_projects/projects/web_script/tool_runing/b5d509f2ddaf74dd2ca07303a86e46a3',

action = "store", help = "Input a outfolder path!"

)

)

logs=c()

#stx=rbind()

tryCatch({

Args <- commandArgs()

opt = parse_args(OptionParser(option_list = option_list, usage = "Data press"))

#logs=c(logs,paste0('geting data:',paste0(paste0(names(opt),'=',opt),collapse = ',')))

logs=c(logs,paste0('run clusterprofiler.R-',basename(opt$outfile)))

#library("rjson")

library(jsonlite)

data<-jsonlite::stream_in(file(opt$infile),pagesize = 1000)

dbName=unlist(data$dbMode)

genes=unlist(data$genes)

genefc=unlist(data$genefc)

gmtNames=unlist(data$gmtNames)

gmtGenes=unlist(data$gmtGenes)

pAdjustMethod=unlist(data$pAdjustMethod)#c("holm", "hochberg", "hommel", "bonferroni", "BH", "BY", "fdr")

outFolder=opt$outfile

dbPath='/pub1/data/mg_projects/projects/web_script/source'

logs=c(logs,paste0('input gene length=',length(genes)))

library(clusterProfiler)

gmt2tab<-function(dbName){

gmt_path=paste0(dbPath,'/',dbName)

prex='';

if(dbName=='c2.cp.biocarta.v7.4.symbols.gmt') prex='BIOCARTA_';

if(dbName=='c2.cp.kegg.v7.4.symbols.gmt') prex='KEGG_';

if(dbName=='c2.cp.pid.v7.4.symbols.gmt') prex='PID_';

if(dbName=='c2.cp.reactome.v7.4.symbols.gmt') prex='REACTOME_';

if(dbName=='c2.cp.wikipathways.v7.4.symbols.gmt') prex='WP_';

if(dbName=='c4.cm.v7.4.symbols.gmt') prex='MODULE_';

if(dbName=='c5.go.bp.v7.4.symbols.gmt') prex='GOBP_';

if(dbName=='c5.go.cc.v7.4.symbols.gmt') prex='GOCC_';

if(dbName=='c5.go.mf.v7.4.symbols.gmt') prex='GOMF_';

if(dbName=='c5.hpo.v7.4.symbols.gmt') prex='HP_';

if(dbName=='h.all.v7.4.symbols.gmt') prex='HALLMARK_';

if(prex!=''){

cgeneset=GSEABase::getGmt(gmt_path)

all.tb=rbind()

for(i in 1:length(cgeneset)){

all.tb=rbind(all.tb,cbind(cgeneset[[i]]@setName,cgeneset[[i]]@geneIds))

}

all.tb[,1]=gsub(paste0('^',prex),'',all.tb[,1])

TERM2NAME=data.frame(term =paste0('ID',1:length(unique(all.tb[,1]))),name=unique(all.tb[,1]))

TERM2GENE=data.frame(term =as.character(TERM2NAME[match(all.tb[,1],as.character(TERM2NAME[,2])),1]),gene=all.tb[,2])

return(list(TERM2NAME=TERM2NAME,TERM2GENE=TERM2GENE,subMap=NULL))

}else{

if(dbName=='KEGG'){

ft = tidyfst::import_fst(paste0(dbPath,'/KEGG_map.fst'),as.data.table = F)

TERM2NAME=unique(data.frame(term =ft[,2],name=ft[,3]))

TERM2GENE=data.frame(term =ft[,2],gene=ft[,1])

subMap=unique(data.frame(term =ft[,2],name=ft[,3],ft[,4:5]))

return(list(TERM2NAME=TERM2NAME,TERM2GENE=TERM2GENE,subMap=subMap))

}else if(dbName=='GO'){

ft = tidyfst::import_fst(paste0(dbPath,'/GO_map.fst'),as.data.table = F)

TERM2NAME=unique(data.frame(term =ft[,2],name=ft[,3]))

TERM2GENE=data.frame(term =ft[,2],gene=ft[,1])

subMap=unique(data.frame(term =ft[,2],name=ft[,3],ft[,4]))

return(list(TERM2NAME=TERM2NAME,TERM2GENE=TERM2GENE,subMap=subMap))

}else if(dbName=='Custom'){

TERM2NAME=data.frame(term =paste0('ID',1:length(unique(gmtNames))),name=unique(gmtNames))

TERM2GENE=data.frame(term =as.character(TERM2NAME[match(gmtNames,as.character(TERM2NAME[,2])),1]),gene=gmtGenes)

return(list(TERM2NAME=TERM2NAME,TERM2GENE=TERM2GENE,subMap=NULL))

}

}

return(NULL)

}

logs=c(logs,paste0('geting ',dbName,' DB map'))

#dbName='c2.cp.kegg.v7.4.symbols.gmt'

# dbTab=gmt2tab('KEGG')

#genes=as.character(gene2kegg[,1][1:100])

#pAdjustMethod=c("holm", "hochberg", "hommel", "bonferroni", "BH", "BY", "fdr")[1]

logs=c(logs,paste0('geting ',dbName,' DB map'))

dbTab=gmt2tab(dbName)

cmp=intersect(dbTab$TERM2GENE[,2],genes)

logs=c(logs,paste0('intersect DB Gene length=',length(cmp)))

if(length(cmp)>3){

genes=unique(genes)

enrich.tab=rbind()

if(dbName=='GO'){

#dbTab=gmt2tab("GO")

enrich1=clusterProfiler::enricher(genes, pvalueCutoff = 1

, pAdjustMethod = pAdjustMethod

,minGSSize = 3, maxGSSize = 5000

, qvalueCutoff = 1

,TERM2GENE=dbTab$TERM2GENE[dbTab$TERM2GENE[,1]%in%dbTab$subMap[which(dbTab$subMap[,3]=='BP'),1],],

TERM2NAME = dbTab$TERM2NAME)

logs=c(logs,paste0('succ enriched GO_BP length=',nrow(enrich1)))

if(!is.null(enrich1)){

enrich.tab=rbind(enrich.tab,cbind(enrich1@result,ONT='BP'))

}

enrich2=clusterProfiler::enricher(genes, pvalueCutoff = 1

, pAdjustMethod = pAdjustMethod

,minGSSize = 3, maxGSSize = 5000

, qvalueCutoff = 1

,TERM2GENE=dbTab$TERM2GENE[dbTab$TERM2GENE[,1]%in%dbTab$subMap[which(dbTab$subMap[,3]=='CC'),1],],

TERM2NAME = dbTab$TERM2NAME)

logs=c(logs,paste0('succ enriched GO_CC length=',nrow(enrich2)))

if(!is.null(enrich2)){

enrich.tab=rbind(enrich.tab,cbind(enrich2@result,ONT='CC'))

}

enrich3=clusterProfiler::enricher(genes, pvalueCutoff = 1

, pAdjustMethod = pAdjustMethod

,minGSSize = 3, maxGSSize = 5000

, qvalueCutoff = 1

,TERM2GENE=dbTab$TERM2GENE[dbTab$TERM2GENE[,1]%in%dbTab$subMap[which(dbTab$subMap[,3]=='MF'),1],],

TERM2NAME = dbTab$TERM2NAME)

logs=c(logs,paste0('succ enriched GO_MF length=',nrow(enrich3)))

if(!is.null(enrich3)){

enrich.tab=rbind(enrich.tab,cbind(enrich3@result,ONT='MF'))

}

}else{

enrich=clusterProfiler::enricher(genes, pvalueCutoff = 1

, pAdjustMethod = pAdjustMethod

,minGSSize = 3, maxGSSize = 5000

, qvalueCutoff = 1

,TERM2GENE=dbTab$TERM2GENE,

TERM2NAME = dbTab$TERM2NAME)

logs=c(logs,paste0('succ enriched length=',nrow(enrich)))

if(!is.null(enrich)){

enrich.tab=enrich@result

if(dbName!='KEGG'){

enrich.tab=enrich.tab[,-1]

}else{

enrich.tab=cbind(enrich.tab,dbTab$subMap[match(enrich.tab[,1],dbTab$subMap[,1]),3:4])

}

}

}

if(!is.null(enrich.tab)){

logs=c(logs,'outputing enrich!')

enrich.tab=enrich.tab[order(enrich.tab$p.adjust),]

write.table(enrich.tab,file = paste0(opt$outfile,'/enrichResult.txt'),quote = F,row.names = F,col.names = T,sep = '\t')

logs=c(logs,'output succed enrich!')

#if(dbName=='GO'){

# logs=c(logs,'GOSemSim starting!')

#}

}else{

logs=c(logs,paste0('intersect DB Gene length<3'))

}

#colnames(enrich.tab)

}else{

#go1 <- c("GO:0004022", "GO:0004024", "GO:0004023","GO:0009055", "GO:0020037")

#d <- new("GOSemSimDATA",ont = 'CC',

# metadata = metadata(org.Hs.eg.db))

#bps=c('GO:0001775','GO:0002252')

#orangeCorr=GOSemSim::termSim(bps, bps, d, method = c("Wang", "Resnik", "Rel", "Jiang", "Lin")[1])

#orangeClust <- hclust(dist(orangeCorr, method="euclidean"), method="complete")

#dynamicCut <- dynamicTreeCut::cutreeDynamic(orangeClust, minClusterSize=1, method="hybrid"

# , distM=as.matrix(dist(orangeCorr, method="euclidean"))

# , deepSplit=4, maxCoreScatter=NULL, minGap=NULL

# , maxAbsCoreScatter=NULL, minAbsGap=NULL)

logs=c(logs,paste0('intersect DB Gene length<3'))

}

},error = function(e) {

print(conditionMessage(e))

logs=c(logs,paste0('error:',conditionMessage(e)))

}, finally = {

write.table(logs,file = paste0(opt$outfile,'/run.log'),quote = F,row.names = T,col.names = T,sep = '\t')

})

#library(ggstatsplot)

GO analysis

library(optparse)

option_list <- list(

make_option(c("-i", "--infile"), type = "character", default = '/pub1/data/mg_projects/projects/web_script/tool_runing/48d62680c879ba86522cfdfa2c641491/input.json',

action = "store", help = "Input a exp file path!"

),

make_option(c("-o", "--outfile"), type = "character", default = '/pub1/data/mg_projects/projects/web_script/tool_runing/b5d509f2ddaf74dd2ca07303a86e46a3',

action = "store", help = "Input a outfolder path!"

)

)

logs=c()

#stx=rbind()

tryCatch({

Args <- commandArgs()

opt = parse_args(OptionParser(option_list = option_list, usage = "Data press"))

#logs=c(logs,paste0('geting data:',paste0(paste0(names(opt),'=',opt),collapse = ',')))

logs=c(logs,paste0('run clusterprofiler.R-',basename(opt$outfile)))

#library("rjson")

library(jsonlite)

data<-jsonlite::stream_in(file(opt$infile),pagesize = 1000)

dbName=unlist(data$dbMode)

genes=unlist(data$genes)

genefc=unlist(data$genefc)

gmtNames=unlist(data$gmtNames)

gmtGenes=unlist(data$gmtGenes)

pAdjustMethod=unlist(data$pAdjustMethod)#c("holm", "hochberg", "hommel", "bonferroni", "BH", "BY", "fdr")

outFolder=opt$outfile

dbPath='/pub1/data/mg_projects/projects/web_script/source'

logs=c(logs,paste0('input gene length=',length(genes)))

library(clusterProfiler)

gmt2tab<-function(dbName){

gmt_path=paste0(dbPath,'/',dbName)

prex='';

if(dbName=='c2.cp.biocarta.v7.4.symbols.gmt') prex='BIOCARTA_';

if(dbName=='c2.cp.kegg.v7.4.symbols.gmt') prex='KEGG_';

if(dbName=='c2.cp.pid.v7.4.symbols.gmt') prex='PID_';

if(dbName=='c2.cp.reactome.v7.4.symbols.gmt') prex='REACTOME_';

if(dbName=='c2.cp.wikipathways.v7.4.symbols.gmt') prex='WP_';

if(dbName=='c4.cm.v7.4.symbols.gmt') prex='MODULE_';

if(dbName=='c5.go.bp.v7.4.symbols.gmt') prex='GOBP_';

if(dbName=='c5.go.cc.v7.4.symbols.gmt') prex='GOCC_';

if(dbName=='c5.go.mf.v7.4.symbols.gmt') prex='GOMF_';

if(dbName=='c5.hpo.v7.4.symbols.gmt') prex='HP_';

if(dbName=='h.all.v7.4.symbols.gmt') prex='HALLMARK_';

if(prex!=''){

cgeneset=GSEABase::getGmt(gmt_path)

all.tb=rbind()

for(i in 1:length(cgeneset)){

all.tb=rbind(all.tb,cbind(cgeneset[[i]]@setName,cgeneset[[i]]@geneIds))

}

all.tb[,1]=gsub(paste0('^',prex),'',all.tb[,1])

TERM2NAME=data.frame(term =paste0('ID',1:length(unique(all.tb[,1]))),name=unique(all.tb[,1]))

TERM2GENE=data.frame(term =as.character(TERM2NAME[match(all.tb[,1],as.character(TERM2NAME[,2])),1]),gene=all.tb[,2])

return(list(TERM2NAME=TERM2NAME,TERM2GENE=TERM2GENE,subMap=NULL))

}else{

if(dbName=='KEGG'){

ft = tidyfst::import_fst(paste0(dbPath,'/KEGG_map.fst'),as.data.table = F)

TERM2NAME=unique(data.frame(term =ft[,2],name=ft[,3]))

TERM2GENE=data.frame(term =ft[,2],gene=ft[,1])

subMap=unique(data.frame(term =ft[,2],name=ft[,3],ft[,4:5]))

return(list(TERM2NAME=TERM2NAME,TERM2GENE=TERM2GENE,subMap=subMap))

}else if(dbName=='GO'){

ft = tidyfst::import_fst(paste0(dbPath,'/GO_map.fst'),as.data.table = F)

TERM2NAME=unique(data.frame(term =ft[,2],name=ft[,3]))

TERM2GENE=data.frame(term =ft[,2],gene=ft[,1])

subMap=unique(data.frame(term =ft[,2],name=ft[,3],ft[,4]))

return(list(TERM2NAME=TERM2NAME,TERM2GENE=TERM2GENE,subMap=subMap))

}else if(dbName=='Custom'){

TERM2NAME=data.frame(term =paste0('ID',1:length(unique(gmtNames))),name=unique(gmtNames))

TERM2GENE=data.frame(term =as.character(TERM2NAME[match(gmtNames,as.character(TERM2NAME[,2])),1]),gene=gmtGenes)

return(list(TERM2NAME=TERM2NAME,TERM2GENE=TERM2GENE,subMap=NULL))

}

}

return(NULL)

}

logs=c(logs,paste0('geting ',dbName,' DB map'))

#dbName='c2.cp.kegg.v7.4.symbols.gmt'

# dbTab=gmt2tab('KEGG')

#genes=as.character(gene2kegg[,1][1:100])

#pAdjustMethod=c("holm", "hochberg", "hommel", "bonferroni", "BH", "BY", "fdr")[1]

logs=c(logs,paste0('geting ',dbName,' DB map'))

dbTab=gmt2tab(dbName)

cmp=intersect(dbTab$TERM2GENE[,2],genes)

logs=c(logs,paste0('intersect DB Gene length=',length(cmp)))

if(length(cmp)>3){

genes=unique(genes)

enrich.tab=rbind()

if(dbName=='GO'){

#dbTab=gmt2tab("GO")

enrich1=clusterProfiler::enricher(genes, pvalueCutoff = 1

, pAdjustMethod = pAdjustMethod

,minGSSize = 3, maxGSSize = 5000

, qvalueCutoff = 1

,TERM2GENE=dbTab$TERM2GENE[dbTab$TERM2GENE[,1]%in%dbTab$subMap[which(dbTab$subMap[,3]=='BP'),1],],

TERM2NAME = dbTab$TERM2NAME)

logs=c(logs,paste0('succ enriched GO_BP length=',nrow(enrich1)))

if(!is.null(enrich1)){

enrich.tab=rbind(enrich.tab,cbind(enrich1@result,ONT='BP'))

}

enrich2=clusterProfiler::enricher(genes, pvalueCutoff = 1

, pAdjustMethod = pAdjustMethod

,minGSSize = 3, maxGSSize = 5000

, qvalueCutoff = 1

,TERM2GENE=dbTab$TERM2GENE[dbTab$TERM2GENE[,1]%in%dbTab$subMap[which(dbTab$subMap[,3]=='CC'),1],],

TERM2NAME = dbTab$TERM2NAME)

logs=c(logs,paste0('succ enriched GO_CC length=',nrow(enrich2)))

if(!is.null(enrich2)){

enrich.tab=rbind(enrich.tab,cbind(enrich2@result,ONT='CC'))

}

enrich3=clusterProfiler::enricher(genes, pvalueCutoff = 1

, pAdjustMethod = pAdjustMethod

,minGSSize = 3, maxGSSize = 5000

, qvalueCutoff = 1

,TERM2GENE=dbTab$TERM2GENE[dbTab$TERM2GENE[,1]%in%dbTab$subMap[which(dbTab$subMap[,3]=='MF'),1],],

TERM2NAME = dbTab$TERM2NAME)

logs=c(logs,paste0('succ enriched GO_MF length=',nrow(enrich3)))

if(!is.null(enrich3)){

enrich.tab=rbind(enrich.tab,cbind(enrich3@result,ONT='MF'))

}

}else{

enrich=clusterProfiler::enricher(genes, pvalueCutoff = 1

, pAdjustMethod = pAdjustMethod

,minGSSize = 3, maxGSSize = 5000

, qvalueCutoff = 1

,TERM2GENE=dbTab$TERM2GENE,

TERM2NAME = dbTab$TERM2NAME)

logs=c(logs,paste0('succ enriched length=',nrow(enrich)))

if(!is.null(enrich)){

enrich.tab=enrich@result

if(dbName!='KEGG'){

enrich.tab=enrich.tab[,-1]

}else{

enrich.tab=cbind(enrich.tab,dbTab$subMap[match(enrich.tab[,1],dbTab$subMap[,1]),3:4])

}

}

}

if(!is.null(enrich.tab)){

logs=c(logs,'outputing enrich!')

enrich.tab=enrich.tab[order(enrich.tab$p.adjust),]

write.table(enrich.tab,file = paste0(opt$outfile,'/enrichResult.txt'),quote = F,row.names = F,col.names = T,sep = '\t')

logs=c(logs,'output succed enrich!')

#if(dbName=='GO'){

# logs=c(logs,'GOSemSim starting!')

#}

}else{

logs=c(logs,paste0('intersect DB Gene length<3'))

}

#colnames(enrich.tab)

}else{

#go1 <- c("GO:0004022", "GO:0004024", "GO:0004023","GO:0009055", "GO:0020037")

#d <- new("GOSemSimDATA",ont = 'CC',

# metadata = metadata(org.Hs.eg.db))

#bps=c('GO:0001775','GO:0002252')

#orangeCorr=GOSemSim::termSim(bps, bps, d, method = c("Wang", "Resnik", "Rel", "Jiang", "Lin")[1])

#orangeClust <- hclust(dist(orangeCorr, method="euclidean"), method="complete")

#dynamicCut <- dynamicTreeCut::cutreeDynamic(orangeClust, minClusterSize=1, method="hybrid"

# , distM=as.matrix(dist(orangeCorr, method="euclidean"))

# , deepSplit=4, maxCoreScatter=NULL, minGap=NULL

# , maxAbsCoreScatter=NULL, minAbsGap=NULL)

logs=c(logs,paste0('intersect DB Gene length<3'))

}

},error = function(e) {

print(conditionMessage(e))

logs=c(logs,paste0('error:',conditionMessage(e)))

}, finally = {

write.table(logs,file = paste0(opt$outfile,'/run.log'),quote = F,row.names = T,col.names = T,sep = '\t')

})

GSEA

library(optparse)

option_list <- list(

make_option(c("-i", "--infile"), type = "character", default = '/pub1/data/mg_projects/projects/web_script/tool_runing/689a26b32a93e8cc5345b11f59bfdef9/input.json',

action = "store", help = "Input a exp file path!"

),

make_option(c("-o", "--outfile"), type = "character", default = '/pub1/data/mg_projects/projects/web_script/tool_runing/b5d509f2ddaf74dd2ca07303a86e46a3',

action = "store", help = "Input a outfolder path!"

)

)

logs=c()

#stx=rbind()

tryCatch({

Args <- commandArgs()

opt = parse_args(OptionParser(option_list = option_list, usage = "Data press"))

#logs=c(logs,paste0('geting data:',paste0(paste0(names(opt),'=',opt),collapse = ',')))

logs=c(logs,paste0('run gsea.R-',basename(opt$outfile)))

#library("rjson")

library(jsonlite)

data<-jsonlite::stream_in(file(opt$infile),pagesize = 1000)

exp_path=unlist(data$exp_path)

#exp_path='/pub1/data/mg_projects/projects/web_script/tool_runing/test_data/GSE73452.txt'

#dbPath='/pub1/data/mg_projects/projects/web_script/source'

#dbName='c2.cp.kegg.v7.4.symbols.gmt'

#####Example 1######

#exp_path='/pub1/data/mg_projects/projects/web_script/tool_runing/test_data/GSE73452.txt'

#dbPath='/pub1/data/mg_projects/projects/web_script/source'

#dbName='c2.cp.kegg.v7.4.symbols.gmt'

#outFolder='/pub1/data/mg_projects/projects/web_script/tool_runing/test_data/GSEA'

###########

samples=unlist(data$samples)

groups=unlist(data$groups)

dbName=unlist(data$dbMode)

dbPath=unlist(data$dbPath)

gmtNames=unlist(data$gmtNames)

gmtGenes=unlist(data$gmtGenes)

method=unlist(data$method)#

gene=unlist(data$gene)

geneSplit=unlist(data$geneSplit)

outFolder=opt$outfile

command=NULL

#paste0(round(groups,2),collapse = "','")

#paste0(samples,collapse = "','")

#samples=row.names(dat)

#groups=apply(dat, 1,function(x){

# return (median(x[which(egfr.exp>4.970198)])/median(x[which(egfr.exp<=4.970198)]))

#})

#method='rank'

#samples=samples[1:1000]

#groups=groups[1:1000]

#samples1=samples[order(groups)]

#gmtGenes=c(samples1[sample(1:100,30)],

# samples1[sample(1:200,40)],

# samples1[sample(900:1000,50)])

#gmtNames=c(rep('Signature 1',30),rep('Signature 2',40),rep('Signature 3',50))

#dbName='Custom'

#paste0(gmtNames,collapse = "','")

nxt=TRUE

if(method!='rank'){

dat=data.table::fread(exp_path, sep = "\t",header = T,stringsAsFactors = F,check.names = F

,na.strings="NA",data.table = F,skip = 0,fill=T)

#head(dat)

#dim(dat)

uName=unique(dat[,1])

dat=dat[match(uName,dat[,1]),]

row.names(dat)=dat[,1]

dat=dat[,-1]

all.genes=row.names(dat)

if(method=='gene'){

geneSplit=ceiling(as.numeric(geneSplit))

g_ind=which(row.names(dat)==gene)

if(length(g_ind)==1){

g_exp=as.numeric(dat[g_ind,])

g_q=quantile(g_exp,seq(0,1,0.01))

groups=ifelse(g_exp>g_q[geneSplit+1],'H','L')

samples=colnames(dat)

}else{

nxt=FALSE

logs=c(logs,paste0('#Stop:not found gene ',gene,'!'))

}

}

if(nxt){

smp.cm=intersect(samples,colnames(dat))

if(length(smp.cm)<3){

nxt=FALSE

logs=c(logs,'#Stop:intersect Sample <3!')

}else{

t_ind=which(samples%in%smp.cm)

samples=samples[t_ind]

groups=groups[t_ind]

if(length(table(groups))!=2){

nxt=FALSE

logs=c(logs,'#Stop:intersect Sample Group !=2 !')

}else{

if(sum(table(groups)>2)!=2){

nxt=FALSE

logs=c(logs,'#Stop:intersect Sample Group <2 !')

}

}

}

}

}else{

logs=c(logs,'output rank file!')

dat=cbind(Gene=samples,Value=groups)

write.table(dat,file = paste0(outFolder,'/exp_data.txt')

,quote = F,row.names = F,sep = '\t')

logs=c(logs,'outputed rank file!')

all.genes=unique(samples)

}

#egfr.exp=as.numeric(dat[which(row.names(dat)=='EGFR') ,])

#groups=ifelse(egfr.exp>median(egfr.exp),'H','L')

#samples=colnames(dat)

jar_path=paste0(dbPath,'/MG_GSEA.jar')

gmt_path=paste0(dbPath,'/',dbName)

#outFolder='/pub1/data/mg_projects/projects/web_script/tool_runing/test_data/GSEA'

if(nxt){

if(dbName=='Custom'){

logs=c(logs,'outputing custom gmt')

all.list=list()

gmt_path=paste0(outFolder,'/',dbName,'.gmt')

for(gn in unique(gmtNames)){

t_inds=which(gmtNames==gn)

gens=intersect(unique(gmtGenes[t_inds]),all.genes)

if(length(gens)>5){

gs=GSEABase::GeneSet(setName=gn, setIdentifier=paste0("101")

,geneIds=gens

,GSEABase::SymbolIdentifier())

all.list=c(all.list,list(gs))

}else{

logs=c(logs,paste0('remove ',gn,' in gmt,gene<5'))

}

}

if(length(all.list)>0){

gsc <- GSEABase::GeneSetCollection(all.list)

GSEABase::toGmt(gsc, gmt_path)

logs=c(logs,'outputed Custom.gmt')

}else{

nxt=FALSE

logs=c(logs,'#Gene not found!')

logs=c(logs,'#Stop:remove all Custom GMT!')

}

}else{

library(cli)

gmt=clusterProfiler::read.gmt(gmt_path)

gmt=gmt[gmt[,2]%in%all.genes,]

if(sum(table(gmt[,1])>5)==0){

nxt=FALSE

logs=c(logs,'#Gene not found!')

logs=c(logs,'#Stop:remove all GMT!')

}

}

}

if(nxt){

if(method!='rank'){

logs=c(logs,'maping group!')

exp_dat=dat[,match(samples,colnames(dat))]

sample_dat=cbind(Sample=samples,Group=groups)

logs=c(logs,paste0('run compare[',unique(groups)[1],'#-vs-#',unique(groups)[2],']'));

write.table(sample_dat,file = paste0(outFolder,'/exp_data_sample.txt'),quote = F,row.names = F,sep = '\t')

write.table(cbind(Tag=row.names(exp_dat),exp_dat),file = paste0(outFolder,'/exp_data.txt')

,quote = F,row.names = F,sep = '\t')

logs=c(logs,'outputed group!')

command=paste0('java -jar ',jar_path,' exp_group '

,paste0(outFolder,'/exp_data.txt'),' ',paste0(outFolder,'/exp_data_sample.txt'),' '

,outFolder,' ',gmt_path,' ','false',' ',3,' ',5,' ',5000)

}else{

command=paste0('java -jar ',jar_path,' rank '

,paste0(outFolder,'/exp_data.txt'),' ',1,' ',outFolder,' ',gmt_path,' ','false',' ','3',' ',5,' ',5000)

}

logs=c(logs,'runing GSEA!')

glogs=system(command, intern = T,

ignore.stdout = FALSE, ignore.stderr = FALSE,

wait = TRUE, input = NULL, show.output.on.console = TRUE,

minimized = FALSE, invisible = TRUE)

logs=c(logs,'runed GSEA!')

logs=c(logs,glogs)

getFile=function(fod){

fl=c()

for(d in dir(fod)){

if(fs::is_dir(paste0(fod,'/',d))){

fl1=getFile(paste0(fod,'/',d))

fl=c(fl,paste0(d,'/',fl1))

}else{

fl=c(fl,d)

}

}

return(fl)

}

logs=c(logs,'select file!')

for(d in dir(outFolder)){

#print(d)

#d='7364ec348ae3428c9d9058df1a224ba9.cls'

#logs=c(logs,d)

if(fs::is_dir(paste0(outFolder,'/',d))){

logs=c(logs,paste0('#GSEAed# ',d))

logs=c(logs,paste0('GF:',getFile(paste0(outFolder,'/',d))))

}

}

logs=c(logs,'selected file!')

}

},error = function(e) {

print(conditionMessage(e))

logs=c(logs,paste0('error:',conditionMessage(e)))

}, finally = {

write.table(logs,file = paste0(opt$outfile,'/run.log'),quote = F,row.names = T,col.names = T,sep = '\t')

})

Figure 5

Lasso analysis

library("glmnet")

library('survival')

if(oldFolder!=''){

file.copy(opt$infile,paste0(oldFolder,'/clini.json'),overwrite = T)

load(file = paste0(oldFolder,'/fit1_cv.RData'))

logs=c(logs,'load fit1_cv.RData')

load(file = paste0(oldFolder,'/fit.RData'))

logs=c(logs,'load fit.RData')

load(file = paste0(oldFolder,'/mgdata.RData'))

logs=c(logs,'load mgdata.RData')

logs=c(logs,paste0('reset lambda=',lambda.min))

}else{

oldFolder=opt$outfile

logs=c(logs,paste0('run init',oldFolder))

if(!file.exists(paste0(oldFolder,'/clini.json'))){

write.table('',file = paste0(oldFolder,'/clini.json'),quote = F,row.names = F,sep = '\t')

}

logs=c(logs,paste0('run readdata'))

dat=data.table::fread(exp_path, sep = "\t",header = T,stringsAsFactors = F,check.names = F

,na.strings="NA",data.table = F)

rNames=unique(dat[,1])

rNames=rNames[which(rNames!='')]

dat=dat[match(rNames,dat[,1]),]

row.names(dat)=dat[,1]

dat=dat[,-1]

logs=c(logs,paste0('read data,row=',nrow(dat),',col=',ncol(dat)))

uSamples=unique(samples)

t_inds=match(uSamples,samples)

dat=dat[,match(uSamples,colnames(dat))]

times=times[t_inds]

events=events[t_inds]

logs=c(logs,paste0('clean data,row=',nrow(dat),',col=',ncol(dat)))

#sum(apply(dat1, 2,sd)==0)

dat1=t(dat)

colnames(dat1)=paste0('C',1:ncol(dat1))

time=as.numeric(times)

event=as.numeric(events)

#t.ind=which(time>0)

y=Surv(time,event)

#nfolds=1

#nfolds=3

#dim(dat1)

#sum(time>0)

logs=c(logs,'starting fit cv')

fit1_cv = cv.glmnet(as.matrix(dat1), y, family = "cox", nfolds=nfolds)

logs=c(logs,'end fit cv,start fit')

fit<-glmnet(dat1, y, family = "cox")

logs=c(logs,'end fit')

logs=c(logs,'output PLD data')

tgc=data.frame(lambda=fit1_cv$lambda,cvm=fit1_cv$cvm,cvup=fit1_cv$cvup,cvlo=fit1_cv$cvlo,cvsd=fit1_cv$cvsd)

write.table(tgc,file = paste0(opt$outfile,'/batchPLD.txt')

,row.names = F,col.names = T,quote = F,sep = '\t')

#cv_fit$lambda.min

#cv_fit$lambda.1se

#ifelse(cv_fit$lambda>=cv_fit$lambda.min&cv_fit$lambda<=cv_fit$lambda.1se,ifelse(cv_fit$lambda==lmda,'A','C'),'B')

logs=c(logs,'output Coef data')

fit.coef=fit$beta[(apply(fit$beta,1,function(x){

return(sum(x!=0))

})>0),]

fit.coef=as.matrix(fit.coef)

row.names(fit.coef)=row.names(dat)[match(row.names(fit.coef),colnames(dat1))]

mtx1=rbind(fit$lambda,fit.coef)

write.table(cbind(Tag=row.names(mtx1),mtx1),file = paste0(opt$outfile,'/batchCoef.txt')

,row.names = F,col.names = F,quote = F,sep = '\t')

logs=c(logs,'save RData')

#head(mtx1)

#dim(mtx1)

save(fit1_cv,file = paste0(opt$outfile,'/fit1_cv.RData'))

save(fit,file = paste0(opt$outfile,'/fit.RData'))

mgdata<-cbind(times,events,t(dat))

save(mgdata,file = paste0(opt$outfile,'/mgdata.RData'))

}

#fit.coef[1:10,1:10]

logs=c(logs,paste0('lambda.min=',fit1_cv$lambda.min))

logs=c(logs,paste0('lambda.1se=',fit1_cv$lambda.1se))

logs=c(logs,'set lambda.min')

if(lambda.min==1){

lambda=fit1_cv$lambda.min

}else{

lambda=lambda.min

}

coefficients<-coef(fit,s=lambda)

Active.Index<-which(coefficients[,1]!=0)

if(length(Active.Index)==0){

logs=c(logs,paste0('not found active genes'))

#write.table(cbind('',''),file = paste0(oldFolder,'/coef.txt')

# ,row.names = F,col.names = T,quote = F,sep = '\t')

}else{

genes=row.names(coefficients)[Active.Index]

Active.coefficients<-coefficients[Active.Index]

lst.genes.exp=mgdata[,as.numeric(gsub('C','',genes))+2]

logs=c(logs,paste0('found genes:',paste0(colnames(lst.genes.exp),collapse = '#,#')))

logs=c(logs,paste0('gene coefficients:',paste0(Active.coefficients,collapse = '#,#')))

coef_dat=cbind(Tag=colnames(lst.genes.exp),Coef=Active.coefficients)

write.table(coef_dat,file = paste0(oldFolder,'/coef.txt')

,row.names = F,col.names = T,quote = F,sep = '\t')

#dim(lst.genes.exp)

#head(lst.genes.exp)

riskscore=lst.genes.exp%*%Active.coefficients

risk_OS=cbind(Time=mgdata[,1],Status=mgdata[,2],RiskScore=riskscore[,1])

logs=c(logs,paste0('output riskcore'))

write.table(cbind(Tag=row.names(risk_OS),risk_OS),file = paste0(oldFolder,'/riskscore.txt')

,row.names = F,col.names = T,quote = F,sep = '\t')

logs=c(logs,paste0('output filter gene exp'))

write.table(cbind(Tag=row.names(lst.genes.exp),lst.genes.exp),file = paste0(oldFolder,'/filterGeneExp.txt')

,row.names = F,col.names = T,quote = F,sep = '\t')

logs=c(logs,paste0('all runed'))

#rel=cbind(Time=mgdata[,1],Status=mgdata[,2],RiskScore=riskscore[,1],lst.genes.exp)

#rel=rbind(c(NA,NA,NA,Active.coefficients),rel)

#head(rel)

#row.names(dat)[match(genes,colnames(dat1))]

}

},error = function(e) {

print(conditionMessage(e))

logs=c(logs,paste0('error:',conditionMessage(e)))

}, finally = {

if(oldFolder!=opt$outfile) write.table(logs,file = paste0(oldFolder,'/run.log'),quote = F,row.names = T,col.names = T,sep = '\t')

write.table(logs,file = paste0(opt$outfile,'/run.log'),quote = F,row.names = T,col.names = T,sep = '\t')

})

Random forest analysis

library(randomForest)

mydata<-read.table(" ",header=TRUE,sep=",",row.names = 1)#读取数据

mydata$AVD<-factor(mydata$AVD) #选择结局事件

set.seed(123)

mydata.rf<-randomForest(AVD~变量.,data=mydata,importance=TRUE,proximity=TRUE)

importance_values <- importance(mydata.rf)

mda <- importance_values[, "MeanDecreaseAccuracy"]

mdg <- importance_values[, "MeanDecreaseGini"]

print(mda)

print(mdg)

varImpPlot(mydata.rf, main="Variable Importance")

pre_ran <- predict(mydata.rf,newdata=mydata)

mean_decrease_gini <- mydata.rf$importance[,"MeanDecreaseGini"]

result <- data.frame(Gene = names(mean_decrease_gini), MeanDecreaseGini = mean_decrease_gini)

# Save the result to an Excel file

write.xlsx(result, file = "MeanDecreaseGini.xlsx", sheetName = "Sheet1", row.names = FALSE)

Cox analysis

library('survival')

dat1=matrix(as.numeric(values),ncol = length(colNames),byrow = T)

times=as.numeric(times)

events=as.numeric(events)

logs=c(logs,paste0('clean data,row=',nrow(dat1),',col=',ncol(dat1)))

colnames(dat1)=paste0('C',1:ncol(dat1))

cxDat=cbind(time=times,status=events,dat1)

fmla <- as.formula(paste0("Surv(time, status) ~",paste0(colnames(dat1),collapse = '+')))

logs=c(logs,paste0('start cox'))

cox <- survival::coxph(fmla, data = as.data.frame(cxDat))

#cox$coefficients

#cox$wald.test

res1=cbind(summary(cox)[[7]][,c(2,5)],summary(cox)[[8]][,c(3,4)],summary(cox)[[7]][,1])

row.names(res1)=colNames[match(row.names(res1),colnames(dat1))]

colnames(res1)[5]='coef'

logs=c(logs,paste0('output cox result'))

write.table(cbind(Tag=row.names(res1),res1),file = paste0(opt$outfile,'/mutiCox.txt')

,row.names = F,col.names = T,quote = F,sep = '\t')

if(isStep==1){

logs=c(logs,paste0('step cox'))

cox2=step(cox)

#cox2$anova

logs=c(logs,paste0('steped cox,output'))

res2=cbind(summary(cox2)[[7]][,c(2,5)],summary(cox2)[[8]][,c(3,4)])

row.names(res2)=colNames[match(row.names(res2),colnames(dat1))]

write.table(cbind(Tag=row.names(res2),res2),file = paste0(opt$outfile,'/mutiCoxStep.txt')

,row.names = F,col.names = T,quote = F,sep = '\t')

riskcore=dat1[,match(names(cox2$coefficients),colnames(dat1))]%*%cox2$coefficients

lst.exp=dat1[,match(names(cox2$coefficients),colnames(dat1))]

cxs=summary(cox2)

logs=c(logs,paste0('logtest=',cxs$logtest[3]))

logs=c(logs,paste0('sctest=',cxs$sctest[3]))

logs=c(logs,paste0('waldtest=',cxs$waldtest[3]))

logs=c(logs,paste0('concordance=',cxs$concordance[1]))

}else{

riskcore=dat1[,match(names(cox$coefficients),colnames(dat1))]%*%cox$coefficients

lst.exp=dat1[,match(names(cox$coefficients),colnames(dat1))]

cxs=summary(cox)

logs=c(logs,paste0('logtest=',cxs$logtest[3]))

logs=c(logs,paste0('sctest=',cxs$sctest[3]))

logs=c(logs,paste0('waldtest=',cxs$waldtest[3]))

logs=c(logs,paste0('concordance=',cxs$concordance[1]))

}

risk_OS=cbind(Time=times,Status=events,RiskScore=riskcore[,1])

logs=c(logs,paste0('output riskcore'))

write.table(cbind(Tag=paste0('R',1:length(times)),risk_OS),file = paste0(opt$outfile,'/riskscore.txt')

,row.names = F,col.names = T,quote = F,sep = '\t')

colnames(lst.exp)=colNames[match(colnames(lst.exp),colnames(dat1))]

logs=c(logs,paste0('output lst gene exp'))

write.table(cbind(Tag=paste0('R',1:length(times)),lst.exp),file = paste0(opt$outfile,'/filterGeneExp.txt')

,row.names = F,col.names = T,quote = F,sep = '\t')

logs=c(logs,paste0('runed all'))

},error = function(e) {

print(conditionMessage(e))

logs=c(logs,paste0('error:',conditionMessage(e)))

}, finally = {

write.table(logs,file = paste0(opt$outfile,'/run.log'),quote = F,row.names = T,col.names = T,sep = '\t')

})

Figure 6

Nomogram

library(rms)

getFunList=function(){

all_fun=list()

s1=function(x) return(sur1(cut.time[1],x))

s2=function(x) return(sur1(cut.time[2],x))

s3=function(x) return(sur1(cut.time[3],x))

s4=function(x) return(sur1(cut.time[4],x))

s5=function(x) return(sur1(cut.time[5],x))

s6=function(x) return(sur1(cut.time[6],x))

s7=function(x) return(sur1(cut.time[7],x))

s8=function(x) return(sur1(cut.time[8],x))

s9=function(x) return(sur1(cut.time[9],x))

s10=function(x) return(sur1(cut.time[10],x))

all_fun2=list(s1,s2,s3,s4,s5,s6,s7,s8,s9,s10)

return(all_fun2[1:length(cut.time)])

}

getNomogramData=function(nom){

all_point_data1=rbind()

all_point_data2=rbind()

all_point_data=rbind()

for(i in 1:length(nom)){

if(names(nom)[i]=='total.points'){

#all_point_data=rbind(all_point_data

# ,c('TOL',)

}else if(names(nom)[i]=='lp'){

# all_point_data=rbind(all_point_data

# ,cbind('LP',nom[[i]][[1]],nom[[i]][[2]]))

}else if(substr(names(nom)[i],1,1)=='C'){

all_point_data1=rbind(all_point_data1

,cbind(names(nom)[i]

,nom[[i]][[1]],nom[[i]][[3]]))

}else if(substr(names(nom)[i],1,1)=='P'){

all_point_data2=rbind(all_point_data2

,cbind(names(nom)[i],nom[[i]][[1]],nom[[i]][[2]]))

}

}

return(list(Col=all_point_data1,Row=all_point_data2,LP=cbind(nom$lp$x,nom$lp$x.real),TP=c( min(nom$total.points$x),max(nom$total.points$x))))

}

set.seed(17)

#oldfolder=data$oldFolder

#if(is.null(oldfolder)|oldfolder==''){

cut.time=as.numeric(unlist(data$cutTime))

#inputData=lung[,c(-3,-2)]

tm=as.numeric(unlist(data$times))

ev=as.numeric(unlist(data$events))

vals=unlist(data$values)

valType=as.numeric(unlist(data$colType))

inputData=(matrix(vals,nrow=length(tm),byrow = F))

#class(inputData[,3])='numeric'

#inputData[,3]=as.numeric(inputData[,3])

inputData=as.data.frame(inputData,stringsAsFactors =F)

for(i in 1:ncol(inputData)){

if(valType[i]==1){

inputData[,i]=as.numeric(inputData[,i])

}else{

inputData[which(inputData[,i]==''),i]=NA

}

}

logs=c(logs,paste0('获得矩阵，行：',nrow(inputData),',列：',ncol(inputData)))

colnames(inputData)=paste0('C',1:ncol(inputData))

old_names=colnames(inputData)

colnames(inputData)=paste0('C',1:ncol(inputData))

fum=as.formula(paste0('Surv(time,status)~',paste0(colnames(inputData),collapse = '+')))

ndat=cbind(time=as.numeric(tm),status=as.numeric(ev),inputData)

dd <- datadist(ndat)

options(datadist="dd")

logs=c(logs,'开始进行多因素生存回归！')

md1=cph(fum,data=ndat,x=T,y=T,surv=T)

md2=survival::coxph(fum,data=ndat,x=T,y=T)

ps=summary(md2)[[7]][,5]

if(!md1$fail){

logs=c(logs,'多因素生存回归成功！开始进行nomogram计算')

sur1=Survival(md1)

nom=nomogram(md1,fun=getFunList()

,fun.at = seq(0.05,0.95,by = 0.05)

,funlabel = paste0('P',1:length(cut.time))

)

#plot(nom)

logs=c(logs,'nomogram计算完成')

nom.data=getNomogramData(nom)

logs=c(logs,'获得nomogram数据')

old_names[match(nom.data$Col[,1],colnames(inputData))]

n.res.dat=cbind(old_names[match(nom.data$Col[,1],colnames(inputData))],

nom.data$Col[,2:3])

nom.data$Row[,1]=gsub('P','',nom.data$Row[,1])

result<-rbind(cbind(0,t(nom.data$TP),''),

cbind(1,nom.data$LP,''),

cbind(2,n.res.dat),

cbind(3,nom.data$Row),

cbind(4,1:length(ps),ps,''))

#plot(nom)

logs=c(logs,'保存nomogram数据')

save(md1,cut.time,file = paste0(opt$outfile,'/stat.RData'))

write.table(result,file = paste0(opt$outfile,'/stat.mtx')

,row.names = F,col.names = F,quote = F,sep = '\t')

#dim(ndat)

logs=c(logs,'保存风险得分数据')

predict(md1)->riskscore

cindx=survcomp::concordance.index(x=riskscore[which(!is.na(riskscore))], surv.time=ndat$time[which(!is.na(riskscore))]

, surv.event=ndat$status[which(!is.na(riskscore))],method="noether")

logs=c(logs,paste0('C-index:',paste0(c(cindx$c.index,cindx$lower,cindx$upper,cindx$p.value),collapse = ',')))

riskscore=cbind(as.character(ndat$time),as.character(ndat$status),riskscore)

write.table(riskscore,file = paste0(opt$outfile,'/riskscore.txt')

,row.names = F,col.names = F,quote = F,sep = '\t')

#}else{

# load(file = paste0(oldfolder,'/stat.RData'))

logs=c(logs,'开始计算校准曲线')

get_calibrate=function(cox_result,u_time){

set.seed(123)

all_cal=c()

points=c(2,3,4,5,6,7)

for(m1 in floor(sum(cox_result$n)/points)){

tryCatch({

cal <- calibrate(cox_result, u=u_time, cmethod='KM', m=m1, B=1000)

#plot(cal)

se <- cal[, "std.err"]

ciupper <- function(surv, d) ifelse(surv == 0, 0, pmin(1,surv * exp(d)))

cilower <- function(surv, d) ifelse(surv == 0, 0, surv * exp(-d))

cal1 <- cal[, "KM"]

lw=cilower(cal1,1.959964 * se)

up=ciupper(cal1,1.959964 * se)

pred <- cal[, "mean.predicted"]

cal.corrected <- cal[, "KM.corrected"]

cal.dat=cbind(m1,pred,cal.corrected,cal1,lw,up)

all_cal=rbind(all_cal,cal.dat)

}, error = function(e) {

}, finally = {})

}

return(all_cal)

}

all.calib=rbind()

for(ut in cut.time){

al=get_calibrate(md1,ut)

if(!is.null(al)){

al=al[which(!is.na(al[,3])),]

all.calib=rbind(all.calib,cbind(ut,al))

}

}

logs=c(logs,'获得校准曲线数据')

write.table(all.calib,file = paste0(opt$outfile,'/calibrate.mtx')

,row.names = F,col.names = F,quote = F,sep = '\t')

#}

logs=c(logs,'succ')

#get_calibrate(md1,365)

}else{

capture.output(str(cph(fum,data=ndat,x=T,y=T,surv=T)))->putTxt

logs=c(logs,'在多因素回归是计算失败，数据存在过拟合！')

logs=c(logs,paste0('ERCPH:',putTxt))

}

},error = function(e) {

print(conditionMessage(e))

logs=c(logs,paste0('error:',conditionMessage(e)))

}, finally = {

write.table(logs,file = paste0(opt$outfile,'/run.log'),quote = F,row.names = T,col.names = T,sep = '\t')

})

ROC curve

install.packages("pROC")

library(pROC)

roc_curve <- roc(data$true_labels, data$predicted_probabilities)

plot(roc_curve, main="ROC Curve", col="blue", lwd=2)

auc_value <- auc(roc_curve)

legend("bottomright", legend=paste("AUC =", round(auc_value, 2)), col="blue", lwd=2)

Calibration curve

install.packages("ggplot2")

install.packages("dplyr")

library(ggplot2)

library(dplyr)

calibration_data <- data %>%

mutate(predicted_class = ifelse(predicted_probabilities > 0.5, 1, 0)) %>%

group_by(predicted_class) %>%

summarise(mean_predicted = mean(predicted_probabilities),

actual_rate = mean(true_labels))

ggplot(calibration_data, aes(x = mean_predicted, y = actual_rate)) +

geom_line() +

geom_abline(slope = 1, intercept = 0, linetype = "dashed", color = "red") +

labs(x = "Mean Predicted Probability", y = "Actual Probability", title = "Calibration Curve") +

theme_minimal()

Figure 7

#install.packages("oncoPredict")

#BiocManager::install("sva")

library(oncoPredict)

library(dplyr)

library(tidyverse)

library(vroom)

library(reshape2)

library(ggplot2)

library(ggpubr)

rm(list=ls())

options(stringsAsFactors = F, warn = -1)

GDSC2_Expr <- readRDS("H:/02 _oncoPredict/GDSC2_Expr.rds")

GDSC2_Res <- readRDS("H:/02 _oncoPredict/GDSC2_Res.rds")

GDSC2_Res <- exp(GDSC2_Res)

exp <- read.csv("H:/02 _oncoPredict/exp.csv",row.names=1)

g <- read.table("g.txt")

library(readr)

group <- read_csv("group.csv")

testExpr <- as.matrix(exp[rownames(exp) %in% g$V1,])

calcPhenotype(trainingExprData = GDSC2_Expr,

trainingPtype = GDSC2_Res,

testExprData = testExpr,

batchCorrect = 'eb',

powerTransformPhenotype = TRUE,

removeLowVaryingGenes = 0.2,

minNumSamples = 10,

printOutput = TRUE,

removeLowVaringGenesFrom = 'rawData')

res <- read.csv("H:/02 _oncoPredict/calcPhenotype_Output/DrugPredictions.csv",row.names=1)

##res <- fread("calcPhenotype_Output/DrugPredictions.csv")

#res <- as.data.frame(res)

#rownames(res) <- res$V1

#res <- res[,-1]

dat_GDSC2_res <- cbind(group,res[group$case_submitter_id,])

colnames(dat_GDSC2_res)

library(ggsci)

dat_GDSC2_res$group <- factor(dat_GDSC2_res$group,

levels = c("low","high"))

# Assuming "group" and "dat_GDSC2_res" are already loaded in your environment

# Loop through columns in res, starting from the 2nd column

for (i in 2:ncol(dat_GDSC2_res)) {

# Create a data frame for the current variable

Cam <- data.frame(Sensitivity = dat_GDSC2_res[, i], Group = dat_GDSC2_res$group)

# Create a boxplot with Wilcoxon rank sum test

boxplot <- ggboxplot(Cam, x = "Group", y = "Sensitivity", fill = "Group",

xlab = "Group",

ylab = paste0(colnames(dat_GDSC2_res)[i], " Sensitivity(IC50)"),

legend.title = "Group",

palette = c("#6666CC", "#CC0033")) +

stat_compare_means(aes(label = ..p.signif..),

comparisons = list(c("low", "high")),

method = "wilcox.test") # Change the method to "wilcox.test"

# Save the boxplot as a PDF file

pdf(file = paste0(colnames(dat_GDSC2_res)[i], "_boxplot.pdf"), width = 5, height = 5)

print(boxplot)

dev.off()

}

wilcox_results <- data.frame()

# Loop through columns in res, starting from the 2nd column

for (i in 2:ncol(dat_GDSC2_res)) {

sensitivity_values <- as.numeric(dat_GDSC2_res[, i])

wilcox_res <- wilcox.test(sensitivity_values ~ dat_GDSC2_res$group)

wilcox_results <- rbind(wilcox_results,

data.frame(

Drug = colnames(dat_GDSC2_res)[i],

W_statistic = wilcox_res$statistic,

p_value = wilcox_res$p.value

)

)

}

write.csv(wilcox_results, file = "wilcox_results.csv", row.names = FALSE)
