# Supplementary material for: Molecular biomarkers for the prognosis of breast cancer: role of amino acid metabolism genes
Source: J Physiol Biochem. 2025 Jun 10;81(2):441–57. doi: 10.1007/s13105-025-01088-5 (PMC12279611; doi:10.1007/s13105-025-01088-5)
Supplement: Supplementary file 3 — Supplementary Material 3 [file 13105_2025_1088_MOESM3_ESM.pdf]

AJE®

Editing Certificate

This document certifies that the manuscript

Molecular Biomarkers for Prognosis of Breast Cancer: Role of Amino Acid Metabolism Genes

prepared by the authors

Yudong Zhou, Shibo Yu, Lizhe Zhu, Yalong Wang, Chenglong Duan, Danni Li, Jinsui Du, Jiaqi Zhang, Jianing Zhang, Ruichao Ma, Jianjun He, Yu Ren, Bin Wang

was edited for proper English language, grammar, punctuation, spelling, and overall style by one or more of the highly qualified English speaking editors at AJE.

This certificate was issued on **January 27, 2025** and may be verified on the AJE website using the verification code **8608-59B4-AFA7-D347-7072**.

Neither the research content nor the authors' intentions were altered in any way during the editing process. Documents receiving this certification should be English-ready for publication; however, the author has the ability to accept or reject our suggestions and changes. To verify the final AJE edited version, please visit our verification page at aje.com/certificate. If you have any questions or concerns about this edited document, please contact AJE at support@aje.com.

AJE provides a range of editing, translation, and manuscript services for researchers and publishers around the world.
For more information about our company, services, and partner discounts, please visit aje.com.
